# Maximizing the Performance of Similarity-Based Virtual Screening Methods by Generating Synergy from the Integration of 2D and 3D Approaches

**DOI:** 10.3390/ijms23147747

**Published:** 2022-07-13

**Authors:** Ningning Fan, Steffen Hirte, Johannes Kirchmair

**Affiliations:** 1 Center for Bioinformatics (ZBH), Department of Informatics, Faculty of Mathematics, Informatics and Natural Sciences, Universität Hamburg, 20146 Hamburg, Germany; fan@zbh.uni-hamburg.de; 2 Division of Pharmaceutical Chemistry, Department of Pharmaceutical Sciences, Faculty of Life Sciences, University of Vienna, 1090 Vienna, Austria; steffen.hirte@univie.ac.at; 3 Vienna Doctoral School of Pharmaceutical, Nutritional and Sport Sciences (PhaNuSpo), University of Vienna, 1090 Vienna, Austria

**Keywords:** virtual screening, virtual screening strategies, shape-based virtual screening, similarity-based virtual screening, molecular fingerprints, benchmarking

## Abstract

Methods for the pairwise comparison of 2D and 3D molecular structures are established approaches in virtual screening. In this work, we explored three strategies for maximizing the virtual screening performance of these methods: (i) the merging of hit lists obtained from multi-compound screening using a single screening method, (ii) the merging of the hit lists obtained from 2D and 3D screening by parallel selection, and (iii) the combination of both of these strategies in an integrated approach. We found that any of these strategies led to a boost in virtual screening performance, with the clearest advantages observed for the integrated approach. On test sets for virtual screening, covering 50 pharmaceutically relevant proteins, the integrated approach, using sets of five query molecules, yielded, on average, an area under the receiver operating characteristic curve (AUC) of 0.84, an early enrichment among the top 1% of ranked compounds (EF1%) of 53.82 and a scaffold recovery rate among the top 1% of ranked compounds (SRR1%) of 0.50. In comparison, the 2D and 3D methods on their own (when using a single query molecule) yielded AUC values of 0.68 and 0.54, EF1% values of 19.96 and 17.52, and SRR1% values of 0.20 and 0.17, respectively. In conclusion, based on these results, the integration of 2D and 3D methods, via a (balanced) parallel selection strategy, is recommended, and, in particular, when combined with multi-query screening.

## 1. Introduction

Compounds which are structurally similar are likely to have similar physicochemical and biological properties. A wide range of methods in cheminformatics are successfully exploiting this *Similar Property Principle* [1,2] for the identification of bioactive compounds (virtual screening [3,4]), the assessment of a compound’s bioactivity profile (target prediction [5]), the optimization of biological and physicochemical properties (quantitative structure–activity relationship, QSAR modeling [6,7] and quantitative structure–property relationship, QSPR modeling [8]), and toxicity prediction (e.g., read-across approaches [9]), among many other applications.

In the context of virtual screening, examples of popular approaches include 2D methods, ranking the compounds of a screening database according to the similarity of their molecular fingerprints (e.g., ECFP fingerprints and derivatives thereof) and those of a compound of interest, and 3D methods, comparing pairs of compounds based on the similarity of their molecular shapes (often, chemical properties projected onto these shape representations are also considered in the similarity assessment).

In recent years, a large number of studies have been published that seek to identify the best method and technical setup for similarity-based virtual screening. For example, Krüger et al. [10] compared the virtual screening performance of 2D fingerprint-based and feature tree-based methods with that of ROCS, a 3D screening engine that analyzes the aligned molecular shapes (and chemical features, often referred to as “color”) of small molecules (and also with that of a variety of docking approaches). They investigated these methods by the example of angiotensin-converting enzyme (ACE), cyclooxygenase 2 (COX2), thrombin and human immunodeficiency virus 1 (HIV-1) protease. For each of the four targets, the researchers compiled a set of 50 known active compounds and 950 presumed inactive compounds from the MDL Drug Data Report (MDDR) [11]. They found that in three out of four cases, the 3D screening method ROCS yielded substantially higher early enrichment factors (i.e., among the top 1% of the ranked molecules) than the 2D screening methods and docking. These findings were in contrast to those of Venkatraman et al. [12], who reported that methods based on 2D molecular fingerprints in general outperform 3D shape-based methods. The observations of Venkatraman et al. were based on tests of five 2D fingerprint-based methods, including the Daylight Fingerprints [13], MACCS keys [14], and MOLPRINT 2D fingerprints [15], as well as five 3D shape-based screening engines (i.e., ESHAPE3D [16], PARAFIT [17], ROCS, SHAEP [18] and USR [19]) on the Directory of Useful Decoys (DUD) [20]. In this setup, the fingerprint-based methods obtained area under the receiver operating characteristic curve (AUC) values between 0.70 (MOLPRINT 2D fingerprint) and 0.76 (Daylight fingerprint and others), and the 3D shape-based method obtained AUC values between 0.44 (ESHAPE3D) and 0.70 (ROCS; considering molecular shape and chemical properties). The authors postulate that the inferior performance of the 3D methods is related to the fact that only a single conformation was used to represent each query molecule. However, an earlier study indicates that the use of multiple conformations to represent a query molecule may not substantially improve the performance of 3D methods (as long as the single query conformation is of good quality and consistent with the conformer ensembles generated for each of the molecules in the screening set) [21]. It is important to note that the DUD (and many other benchmark data sets) contains significant biases which have been shown to skew the results of performance tests [20,22,23,24,25].

Hu et al. [26] compared the virtual screening performance of 14 2D fingerprints (including MDL Keys, atom pair-based fingerprints and various types of ECFP, ECFC, FCFP and FCFC fingerprints) and four 3D screening methods implemented in Phase [27] on the DUD_LIB_VS_1.0 [28], a subset of the DUD that is designed for ligand-based virtual screening. They found that 2D fingerprints (e.g., ECFP_2) yielded, on average, slightly better performance than the 3D shape-based methods implemented in Phase (e.g., shape_ele) in terms of mean AUC (AUC of 0.85 for ECFP_2 compared to 0.83 for shape_ele) and early enrichment (the receiver operating characteristics enrichment (ROCE) value at 0.5% of the rank-ordered hit list was 103.74 for ECFP_2 in comparison to 95.9 for shape_ele).

While there is no virtual screening method in existence that consistently outperforms the others, an established key strategy for maximizing the success rates in virtual screening is the fusion of results obtained with different methods and queries [29,30,31]. Shang et al. [32], for example, combined a method based on 2D molecular fingerprints (FP2, which is a Daylight-type path-based fingerprint, or MACCS keys) with a 3D shape-based approach (WEGA [33]) using a hybrid score that is the square root of the product of the individual scores. On 40 selected targets of the DUD-E data set utilized as benchmark data set in their work, the integrated approach (with the hybrid score) yielded an average EF1% value of 22.98 while the average EF1% values for the 2D and the 3D approaches were 20.79 and 16.64, respectively.

Pavadai et al. [34] searched an in-house database of steroid-type natural products for compounds with anti-plasmodial activity. They used fusidic acid, an established antibiotic and known anti-plasmodial compound, as query for similarity searches with different 2D molecular fingerprints (e.g., FCFP_2, ECFC_4 and FCFC_4) and 3D shape-based methods (e.g., shape-based screening with Phase). From the hit lists obtained with the individual screening methods, the authors selected a total of 27 compounds for testing in an anti-plasmodial assay with *Plasmodium falciparum*. Among these 27 compounds, four were found to exhibit activity, with IC_50_ values between 1 and 4 μM.

Today, substantial information on the performance of 2D and 3D similarity methods in virtual screening is available. Studies have shown that the combination of 2D and 3D methods can yield better results. However, the scope of these studies is limited to a few targets. No systematic analysis of the capacity of different strategies to create synergy from the combination of 2D and 3D methods has been reported, as of yet.

In this work, we systematically explored the capacity of a variety of strategies to maximize the virtual screening success rates of similarity-based approaches. More specifically, we explored one of the most popular 2D molecular representations, Morgan fingerprints, and a leading 3D screening engine comparing the shape (and chemistry) of aligned molecular structures, ROCS. The methods and strategies were tested on bioactivity data sets compiled for 50 prominent therapeutic targets (Table 1): 23 enzymes, 21 membrane receptors, three ion channel receptors, two transcription receptors and one transporter receptor. This list of 50 targets was elaborated previously, by Heikamp and Bajorath, as part of a large-scale benchmarking study of similarity-based approaches for virtual screening [35]. For our study, we collected bioactive compounds for these 50 targets from the ChEMBL database. In order to obtain realistic data sets for virtual screening, we joined, individually for each target, the confirmed bioactive and confirmed inactive compounds from the ChEMBL database (excluding any compounds used for testing the screening methods) with one and the same set of 94,685 presumed inactive compounds, selected by random sampling from the Enamine HTS Collection [36] (one of the leading libraries of purchasable screening compounds). We note that a small fraction of the presumed inactive compounds from the Enamine HTS Collection (usually in the range of 0.1%) would in fact show activity on the target when tested in vitro. This fact should be taken into account when interpreting the results presented in this work.

The virtual screening experiments were performed using, for each of the 50 investigated targets, 30 randomly selected, active compounds as queries (which were removed from the screening data sets). For the purpose of 3D virtual screening, these query molecules were represented by a single, low-energy 3D conformation whereas the molecules of the individual screening data sets were represented by ensembles of a maximum of 200 conformers.

**Figure 1:**
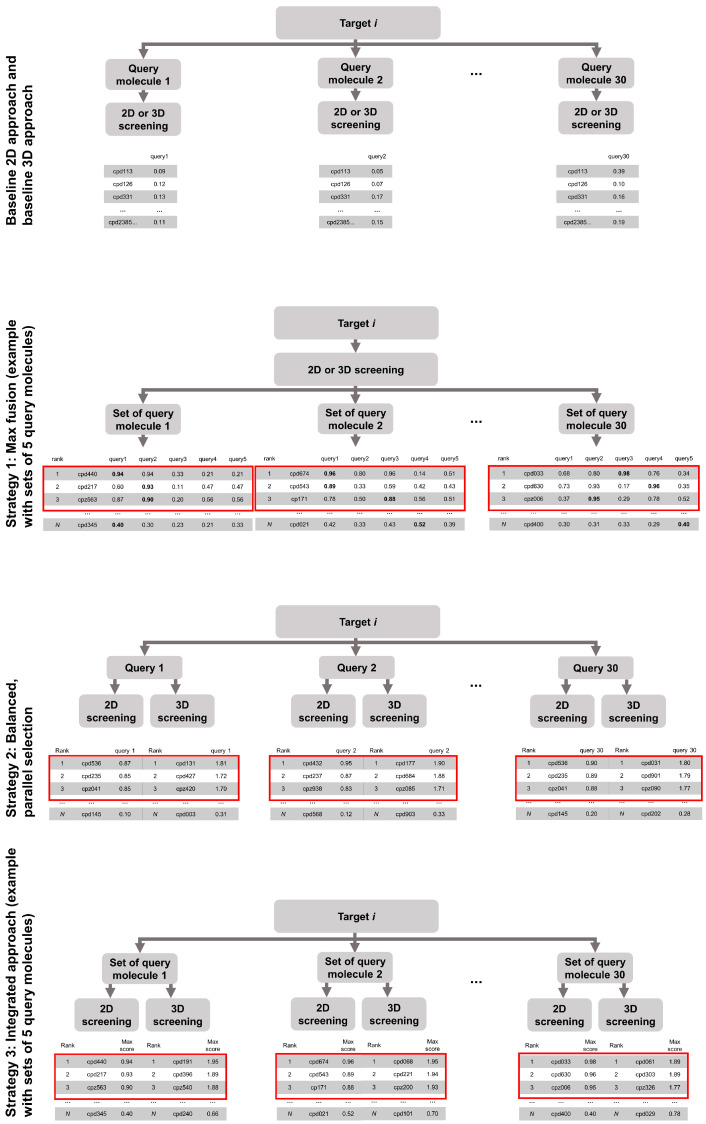
Overview of the investigated virtual screening approaches and strategies. The red boxes highlight the compounds selected by the individual hit selection strategies.

We start this report with the assessment of the performance of the 2D fingerprint-based approach and the 3D molecular shape-based method when used on their own, hence, defining the baseline performance of the methods. We then evaluate the capacity of three different strategies to improve virtual screening performance (Figure 1):

- Strategy 1: the merging of hit lists obtained from screening with a set of query molecules
- Strategy 2: the merging of the hit lists obtained from 2D and 3D screening, and
- Strategy 3: the combination of Strategy 1 and Strategy 2.

## 2. Results

The performance of the 2D fingerprint-based approach and the 3D molecular shape-based approach, in combination with different strategies aiming to improve virtual screening performance, was investigated from three perspectives:

1. Overall virtual screening performance. The overall virtual screening performance reflects the ability of a method to discriminate active from inactive compounds. We quantified the overall screening performance using the AUC as metric.
2. Early enrichment. Early enrichment describes the ability of a method to rank active compounds early in the hit list. We quantified early enrichment using enrichment factors calculated for the *k*% top-ranked molecules, where *k* = 1, 3, 5 or 10.
3. Scaffold recovery rate. The scaffold recovery rate (SRR) describes the ability of a method to identify active compounds of diverse molecular structures. The SRR was calculated as the proportion of Murcko scaffolds [37] of known active compounds that were ranked early in the hit list (i.e., among the *k*% top-ranked molecules, where *k* = 1, 3, 5 or 10).

### 2.1. Baseline Performance of the 2D and 3D Virtual Screening Methods

**Figure 2:**
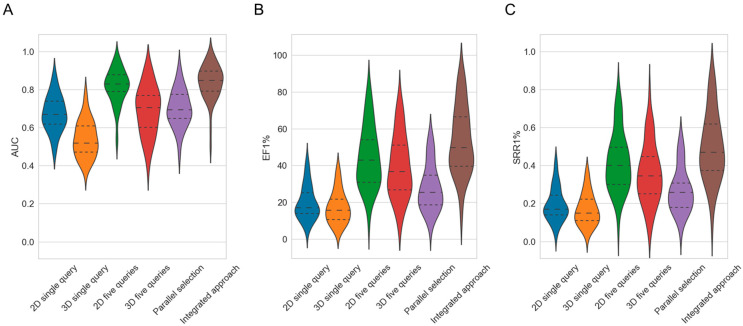
Violin plots showing the distributions of (**A**) AUC, (**B**) EF1% and (**C**) SRR1% values obtained by the different approaches across the 50 investigated targets, with “2D single query” representing the baseline performance of the 2D fingerprint-based screening method, “3D single query” representing the baseline performance of the 3D shape-based method, “2D five queries” representing the performance of the 2D method using five query molecules (Strategy 1), “3D five queries” representing the performance of the 3D method using five query molecules (Strategy 1), “parallel selection” representing the performance of the 2D and 3D approach when combined using the balanced, parallel selection method (Strategy 2), and “integrated approach” representing the combination of Strategy 1 and Strategy 2 (a combined hit list was generated by selecting an equal number of hits from the 2D and 3D methods which were each run with sets of five query molecules). The dashed lines mark the quartiles of the distributions.

Averaged across the 50 investigated targets, the 2D approach showed a better screening performance than the 3D approach (Figure 2A and Table 2): The average AUC (to which each target is contributing a single AUC value that itself is an averaged AUC across the 30 screening runs with different molecule queries) obtained by the 2D approach was 0.68, whereas for the 3D approach the average AUC was just 0.54 (the difference in the average AUC obtained by the two methods was statistically significant, as confirmed by the paired Wilcoxon signed-rank test; *p*-value 2.31 × 10^−9^). However, even a mean AUC of 0.68 was an indication of only mediocre virtual screening performance.

For the individual targets, the AUC values (which are averages across the 30 query molecules of a target) ranged from 0.47 (for the neuropeptide Y receptor type 1) to 0.88 (for the β_2_-adrenergic receptor) for the 2D approach, and from 0.36 (cytochrome P450 2C9) to 0.78 (leukotriene A4 hydrolase) for the 3D approach. For information on the AUC values obtained for the individual targets the reader is referred to Table S1.

With respect to the early enrichment, the 2D approach also fared, on average, better than the 3D approach (Figure 2B and Table 2). The EF1%, averaged across the 50 targets, was 19.96 for the 2D approach compared to 17.52 for the 3D approach (*p*-value 4.34 × 10^−4^). Thereby, the EF1% obtained by the 2D method ranged from 3.86 (cytochrome P450 2C9) to 43.84 (β_2_-adrenergic receptor), and that of the 3D method from 3.08 (cytochrome P450 2C9) to 39.00 (melatonin receptor 1B; the enrichment factors for the individual target are provided in Table S2). 

Likewise, the averaged EF10% values were 3.69 for the 2D similarity method and 2.87 for the 3D similarity method (*p*-value 4.73 × 10^−8^). For the 2D method, EF10% values ranged from 1.23 (cytochrome P450 2C9) to 7.33 (β_2_-adrenergic receptor), whereas for the 3D method they ranged from 0.83 (cytochrome P450 2C9) to 5.89 (melatonin receptor 1B). The prevalence of cytochrome P450 2C9 as a challenging target was not surprising, given the broad range of substrates this enzyme can transform (cytochrome P450 2C9 is the only cytochrome P450 enzyme among the 50 investigated targets).

Looking more closely at the individual targets, the 2D method yielded better EF1% values than the 3D method for 37 out of the 50 proteins (Table S2). The biggest advantage of the 2D approach over the 3D approach was observed for the β_2_-adrenergic receptor data set (EF1% 43.84 vs. 22.13). Among the cases in which the 3D approach outperformed the 2D approach, the maximum difference in early enrichment performance was observed for the orexin receptor 2 (EF1% 19.99 vs. 16.29).

Unsurprisingly, the observations of superior EF1% and EF10% performance of the 2D approach were consistent with the trends observed for EF3% and EF5% (Table S3). It is worth mentioning though that the EFs obtained by one and the same virtual screening method differed, in part, substantially across the individual targets and even across the individual query molecules for the same target (Tables S2, S4 and S5). This observation corroborated the importance of the query molecule to virtual screening success.

With respect to SRRs, the observed trends were consistent with those observed for AUC and EF: The 2D method retrieved, on average, across the 50 targets, 20% of the known bioactive molecular scaffolds among the top 1% of the ranked molecules (i.e., SRR1% = 0.20). In comparison, the 3D method recovered only 17% on average (SRR1% = 0.17; *p*-value 2.25 × 10^−4^; Figure 2C). For the 2D method, the SRR1% varied between 0.03 (cytochrome P450 2C9) and 0.42 (β_2_-adrenergic receptor), whereas for the 3D method, the rates ranged from 0.02 (cytochrome P450 2C9) to 0.37 (melatonin receptor 1B). For the top 10% of the ranked molecules, the average SRR was 0.38 for the 2D method and 0.29 for the 3D method (*p*-value 1.62 × 10^−8^). Additional information on SRRs is provided in Tables S3–S6.

Looking at the individual targets, for 34 out of 50 targets the SRR1% was better for the 2D method than for the 3D method (Table S6). The biggest advantage of the 2D method over the 3D method was observed for the β_2_-adrenergic receptor (SRR1% 0.42 vs. 0.23). Among the minority of cases in which the 3D approach achieved superior SRR1% values, the largest difference was observed for the sphingosine 1-phosphate receptor Edg-1 (SRR1% 0.23 vs. 0.17).

Based on the observations discussed above, we concluded that the baseline virtual screening performance of the 2D approach was at least as good as that of the computationally more expensive 3D approach. We learned that the trends observed for the individual metrics were consistent across the different rank-cutoffs explored (i.e., top 1%, top 3%, top 5% and top 10%). Moreover, no major differences between average values and median values of the performance metrics were observed (Tables S3 and S7). For these reasons, further discussions focused on averages and the top 1% of the hit list.

### 2.2. Exploration of Strategies for Maximizing the Success Rates of Similarity-Based Methods for Virtual Screening

#### 2.2.1. Strategy 1: Use of Multiple Compounds as Queries

A promising strategy for maximizing the success rates of similarity-based methods for virtual screening is the use of multiple query molecules, rather than just a single one. In this scenario, a rank-ordered list of compounds (hit list) is obtained for each query. The lists can then be merged into a single list in different ways. In the context of virtual screening, “MAX fusion” is likely the best strategy for merging hit lists [30,38,39,40]. MAX fusion assigns, to each compound in the hit list, the best score obtained for that compound with any of the query molecules.

Considering the fact that in a real-world scenario the number of known bioactive compounds for a target of interest is usually scarce, we explored multi-query screening with one, two, three, four and five query molecules per target (note that if a single query molecule is used, the method is identical to the baseline method). The sets of query molecules were assembled by random selection using a defined seed that ensured that the smaller query sets were always a subset of the set composed of five queries (the five queries were removed from the screening data sets prior to the conduction of the screening experiments). Importantly, the sets of query molecules used for screening with the 2D approach and the 3D approach were identical, hence, enabling the direct comparison of the two methods. The process of query selection and virtual screening was repeated 30 times (with unique random seeds between 0 and 29) to allow the assessment of the variance in the experiment. In total 300 virtual screens were conducted for each of the 50 targets, resulting from the use of five different sets of query molecules (composed of one, two, three, four and five query molecules) during each of the 30 repetitions, with two virtual screening methods (2D and 3D).

**Figure 3:**
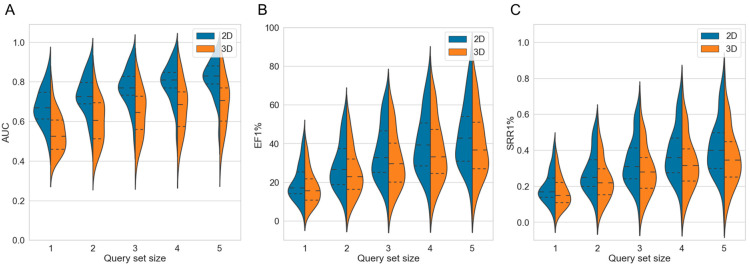
Violin plots showing the distributions of (**A**) AUC, (**B**) EF1% and (**C**) SRR1% values obtained by the 2D fingerprint-based method and the 3D shape-based method over the 50 investigated targets, with different sizes of the query sets. The dashed lines mark the quartiles of the distribution.

Averaged across the 50 targets, both the 2D method and the 3D method showed clear gains in AUC values, EF values and SRRs (Table S8): For the 2D approach, the mean AUC across 50 targets improved gradually from 0.68 in single-query mode (which is identical to the baseline approach) to 0.82 when using a set of five query molecules (Table 2 and Figure 3A). For the 3D approach, the mean AUC increased from 0.54 to 0.69, respectively. For the individual targets, the AUC values (using five query molecules) ranged from 0.52 (cytochrome P450 2C9) to 0.97 (melatonin receptor 1B) for the 2D approach, and from 0.39 (cytochrome P450 2C9) to 0.92 (nicotinic acid receptor 1) for the 3D approach (performance on the individual targets reported in Tables S9 and S10).

As observed for the AUC values, the EF1% and SRR1% both also increased with either approach as more query molecules were used. For the 2D approach, the averaged EF1% increased from 19.96 in single-query mode to 44.59 when using sets of five molecule queries (Figure 3B; additional data provided in Table S8). For the 3D approach, the averaged EF1% increased from 17.52 to 39.48, respectively. For the individual targets, the EF1% values with five molecule queries ranged from 9.89 (cytochrome P450 2C9) to 82.24 (C5a anaphylatoxin chemotactic receptor) with the 2D approach, and from 9.50 (carbonic anhydrase XII) to 76.58 (nicotinic acid receptor 1) with the 3D approach.

The 2D approach obtained an SRR1% of 0.20 when using a single query molecule while the same approach obtained an SRR1% of 0.42 when using sets of five query molecules (Figure 3C and Table S8). Likewise, the 3D approach, using a single query molecule, obtained an SRR1% of 0.17 whereas the same approach using sets of five query molecules obtained an SRR1% of 0.36. When five query molecules were used for the individual targets, the SRR1% values ranged from 0.07 (cytochrome P450 2C9) to 0.80 (C5a anaphylatoxin chemotactic receptor) for the 2D approach, and from 0.06 (cytochrome P450 2C9) to 0.79 (nicotinic acid receptor 1) for the 3D approach.

The early EF and SRR values of both the 2D and the 3D approach increased consistently as more query molecules were added to the screen (Table 2), and the variance in performance decreased. The reader is referred to Tables S11–S18 for data on the performance of the two approaches on the individual targets.

Averaged across the 50 investigated targets, the 2D approach outperformed the 3D approach in most cases with respect to the overall performance (AUC), early enrichment and early recovery rates, regardless of the number of query molecules used for screening (all *p*-values < 0.05). Only for a minority of targets (10% to 28% of the investigated targets; Table 3), did the 3D approach outperform the 2D method (Tables S9–S18).

#### 2.2.2. Strategy 2: Parallel Selection of Compounds Ranked at Top Positions by the 2D and/or the 3D Virtual Screening Approach

**Figure 4:**
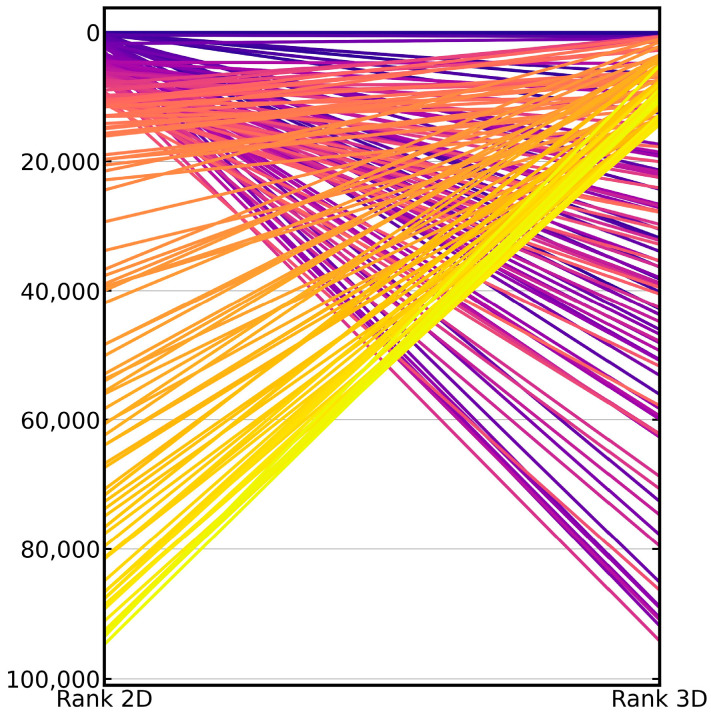
Parallel coordinate plot visualizing the differences in the ranks assigned to the known active compounds by the 2D approach (left) and the 3D approach (right). The plot shows the 100 top-ranked, active compounds reported by either method. From the plot, it is obvious that many of the active compounds that were rightly ranked at top positions by the 2D approach were missed by the 3D approach (in the top-ranked positions) and vice versa. The color spectrum applied to the lines visualizes the ranks assigned to the known active compounds by the 2D approach (dark blue: top ranks; yellow: lowest ranks).

When comparing the results obtained with the 2D fingerprint-based method and the 3D shape-based method, we found that the overlap between the active compounds in the top ranks was low (Figure 4). When looking at Murcko scaffolds, we found that 14% of the scaffolds of the active compounds ranked with the 2D approach among the top 1% ranks (average across the 50 investigated targets) were missed by the 3D approach. Likewise, on average, 12% of the scaffolds underlying the actives ranked among the top 1% of the ranked molecules with the 3D approach were missed by the 2D approach (Table 4). Only 5% of the scaffolds of known actives were ranked, on average, by both methods among the top 1% of the ranked molecules.

Taken together, the observations described above indicate that a well-designed combination of the 2D approach and the 3D approach might well yield better performance, in particular, with respect to the recovery of (structurally diverse) bioactive compounds. The first strategy that we explored in this regard was parallel selection. In parallel selection, an empty hit list is created and filled by sequential, iterative transfer of the hits obtained by the 2D and the 3D approach.

In the case of balanced, parallel selection, equal numbers of hits were selected from both approaches. Any duplicates were removed (i.e., replaced by the next, unique hit molecule). In the case of imbalanced, parallel selection, distinct numbers of hits were transferred from the individual hit lists. For example, when applying a ratio of 9:1 during parallel selection, the empty hit list would be filled with 90% of the compounds resulting from the 2D screen and 10% of the compounds resulting from the 3D screen. Any duplicates were removed and the procedure was repeated until all the slots of the new hit list were filled.

**Figure 5:**
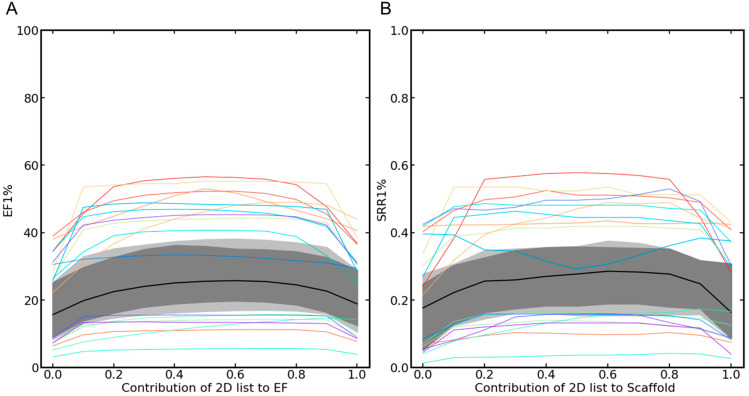
Development of the (**A**) EF1% and (**B**) SRR1% as the proportion of hits selected from the 2D approach was increased. The values on the left side of the two plots reflect the performance of the baseline 3D approach (as no hits from the 2D approach were included in the combined hit list) and the values on the right side show the performance of the baseline 2D approach (as no hits, obtained with the 3D approach, were included in the combined hit list). In the center of the graphs, the performance of the balanced, parallel selection approach is shown. In most cases, the balanced selection was the most favorable setup with respect to all performance measures considered. The median curve is shown as a black line; the envelope of the 50% central region and the maximum non-outlying envelope are visualized in gray, and the outlier curves are shown as colored lines.

Starting from a (combined) hit list that was, at the beginning, composed exclusively of the hits obtained with the 3D approach, Figure 5A and Table S19 show how the early enrichment factors evolved as the hits were increasingly replaced by compounds assigned top ranks by the 2D approach. The graphs show that, in most cases, the best early enrichment rates were obtained when balanced weighing of the 2D and the 3D approach was applied (in other words, in cases where identical numbers of compounds were selected from the two rank-ordered hit lists; see also Table S19). Consistent observations were made also for the SRRs (Figure 5B and Table S19).

With respect to global screening performance, the mean AUC (averaged across the 50 targets) obtained with the balanced, parallel selection approach (applying equal weights to the 2D and the 3D approach) was higher (0.70) than that obtained with the baseline 2D method (0.68; *p*-value 4.03 × 10^−5^) and the baseline 3D method (0.54; *p*-value 7.49 × 10^−10^). Regarding the performance of the methods on the individual targets, the AUC for the parallel approach ranged from 0.45 (cytochrome P450 2C9) to 0.92 (melatonin receptor 1B; see Table S1 for performance data for the individual targets).

With respect to early enrichment, the balanced, parallel selection approach fared substantially better (EF1% 28.16) than the baseline 2D approach (EF1% 19.96; *p*-value of 8.53 × 10^−10^) and the baseline 3D approach (EF1% = 17.52; *p*-value of 7.56 × 10^−10^). For the individual targets, the EF1% ranged from 5.53 (cytochrome P450 2C9) to 56.52 (somatostatin receptor 5; see Table S2).

Balanced, parallel selection identified, on average, 27% of the scaffolds of the active compounds among the top 1% of the ranked compounds, whereas the baseline 2D method identified just 20% (*p*-value 9.85 × 10^−10^) and the baseline 3D method identified only 17% (*p*-value 7.47 × 10^−10^). The SRR1% for the individual targets ranged from 0.04 (cytochrome P450 2C9) to 0.55 (C5a anaphylatoxin chemotactic receptor; Table S6).

#### 2.2.3. Strategy 3: Integration of Multi-Query Screening and Balanced, Parallel Selection

The findings described in the previous sections suggest that the integration of multi-query screening (Strategy 1) with balanced, parallel selection (Strategy 2) could further increase virtual screening success rates. Therefore, we explored Strategy 3, in which the final hit list was generated by selecting an equal number of top-ranked compounds from the rank-ordered lists of compounds obtained from multi-query screening (with the 2D approach and the 3D approach), using MAX fusion.

**Figure 6:**
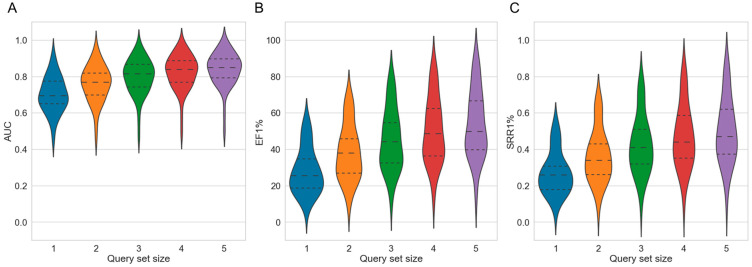
Violin plots showing the distributions of (**A**) AUC, (**B**) EF1% and (**C**) SRR1% values obtained by the integrated method over the 50 investigated targets as a function of the size of the sets of query molecules. The dashed lines mark the quartiles of the distributions.

As reported in Figure 6A and Table S20, the integrated approach did indeed yield better results. The mean AUC (i.e., the average across the 50 investigated targets) for the integrated approach increased from 0.70 when using a single query molecule (in this case, the integrated approach is identical to the balanced, parallel selection mode) to 0.84 when using sets of five query molecules. The AUC values were significantly higher than the AUC values obtained by the corresponding multi-query 2D approach (AUC 0.82 when using sets of five query molecules; *p*-value 2.82 × 10^−6^) and multi-query 3D approach (AUC 0.69 when using sets of five query molecules; *p*-value = 1.09 × 10^−9^). The overall performance of the individual targets varied from 0.50 (cytochrome P450 2C9) to 0.99 (melatonin receptor 1B; see Table S1). The target-specific values are reported in Table S21.

With respect to early enrichment, the integrated method also outperformed all other setups (Figure 6B). When using sets of five query molecules, the mean EF1% obtained by the integrated approach was 53.82 compared to 44.59 for the second-best setup, which is the multi-query 2D approach using sets of five query molecules (*p*-value 9.07 × 10^−10^; Table S2). Among the 50 investigated targets, the integrated approach (using sets of five query molecules) obtained higher EF1% values for 48 and 50 targets compared to the multi-query 2D approach (using sets of five query molecules) and the baseline 2D approach (using a single query molecule). The EF1% for the individual targets ranged from 13.94 (cytochrome P450 2C9) to 89.90 (c5a anaphylatoxin chemotactic receptor). The target-specific values are reported in Tables S2, S22 and S23).

For the mean SRR1%, the trends were consistent with the previous observations (Figure 6C). With sets of five query molecules, the SRR1% of the integrated approach was 0.50, which was significantly higher than 0.42 (*p*-value 2.50 × 10^−9^), the value obtained for the second-best setup, which was again the multi-query 2D approach using sets of five query molecules. Across the 50 investigated targets, the SRR1% of the integrated approach (using sets of five query molecules) was higher for 46 and 50 targets, compared to the multi-query 2D approach (using sets of five query molecules) and the baseline 2D approach. The SRR1% for each target was in the range of 0.09 (cytochrome P450 2C9) to 0.88 (c5a anaphylatoxin chemotactic receptor). Target-specific values are reported in Tables S6, S24 and S25.

When comparing the integrated approach with the multi-query 3D screening approach (Strategy 1), all 50 targets (100% of the targets) performed at least equally well in terms of the EF1% (target-specific information is provided in Tables S2 and S22) and SRR1% of the ranking list (Tables S6 and S24).

## 3. Materials and Methods

### 3.1. Data Sets and Data Processing

The full set of bioactivity records for the 50 protein targets (Table 1), investigated by Heikamp and Bajorath in their study of the performance of similarity-based methods utilizing fingerprints [35], was retrieved from the ChEMBL database version 27 [41,42] via its web interface [43]. The bioactivity records were processed with a data preparation protocol published by some of us previously [44]. In brief, this protocol ensures that only high-quality bioactivity and chemical information is used, and it includes a labeling procedure that labels any compound with a “standard_value” (i.e., bioactivity value) of less than 10,000 nanomolar in ‘Kd’, ‘AC50’, ‘IC50’, ‘Ki’, ‘EC50’ or ‘Potency’ as active and any compound with a standard_value greater than 20,000 as inactive.

The rates of active compounds in the ChEMBL-derived bioactivity data sets are substantially higher than those found in typical screening libraries. To simulate virtual screening in a scenario that resembles real-world applications more closely, the inactive compounds were supplemented with 100,000 presumed inactive compounds sourced from the Enamine HTS Collection (data accessed: July 2020) by random selection (unless stated otherwise, all random sampling procedures in this study utilized a random seed of 2506).

All compounds (including those from the ChEMBL database, as well as the 100,000 compounds from the Enamine subset) were filtered, based on molecular weight (any compounds with a molecular weight outside the range of 250 to 1500 Da were discarded) and element types (any compounds composed of atoms other than H, B, C, N, O, F, Si, P, S, Cl, Se, Br, and I were discarded). The salt filter implemented in RDKit [45] was applied to remove the minor components of the salts. In addition, any active compounds without a complete definition of the stereochemical configuration of tetrahedral atoms were discarded. This step was performed to ensure the full definition of any compounds potentially used as queries in 3D virtual screening.

For each of the 50 assembled target data sets, 30 active compounds were selected randomly to serve as queries for virtual screening. In preparation for 3D virtual screening, for all query molecules, a single, low-energy 3D conformation was generated with OMEGA (all settings kept default, except for “-maxconfs” set to “1”) [46,47]. For all confirmed and for all presumed inactive compounds, ensembles of a maximum of 200 conformers were calculated with OMEGA (all settings default, except for the flipper option, which was enabled to enumerate all possible stereochemical configurations for compounds with up to six undefined stereocenters; for any compound with more than six undefined stereocenters, 64 isomers were picked randomly from a larger set of stereoisomers). The composition of the prepared data set is reported in Table 1.

### 3.2. Virtual Screening

Virtual screening based on 2D molecular similarity was performed with the RDKit using Morgan fingerprints (radius = 2; number of bits = 1024). The 3D virtual screening was performed with ROCS [48,49] using default settings, with the TanimotoCombo score [48] used for the ranking of pairs of compounds. The TanimotoCombo score ranges from 0 to 2, with higher values indicating a higher degree of molecular similarity. There are two components to this score that each contributes a value between 0 and 1: The Shape Tanimoto score, which quantifies the similarity of molecules with respect to their molecular shape, and the Color Tanimoto score, which quantifies the similarity of molecules with respect to their pharmacophoric properties.

## 4. Conclusions

In this work, we first benchmarked the virtual screening performance of 2D similarity-based approaches, represented by an approach utilizing Morgan2 fingerprints, and 3D similarity-based approaches, represented by the screening engine ROCS (which takes chemical features into account) on data sets covering 50 pharmaceutically relevant proteins. We then explored three strategies for maximizing the virtual screening performance of these approaches:

- Strategy 1: Screening with one method using multiple query molecules and merging of the hit lists using MAX fusion
- Strategy 2: Screening with both methods and merging of the hit lists by parallel selection, and
- Strategy 3: Combination of strategies (1) and (2).

The performance of individual methods and strategies was quantified and compared with respect to the overall ranking performance (AUC), early enrichment (EF1% to EF10%) and the early recovery of the molecular scaffolds (SRR1% to SRR10%).

As a first important observation, we found that, in general, the 2D approach outperformed the computationally more expensive 3D approach in virtual screening. While the AUC values (averaged across the 50 investigated targets) obtained by the baseline 2D approach and the baseline 3D approach were only moderate (0.68 and 0.54, respectively), early enrichment was high (EF1% 19.96 and 17.52, respectively). Among the top 1% of the ranked molecules, the 2D method recovered 20% of the scaffolds of known active compounds whereas the 3D method recovered 17%.

As a second important observation, we found that virtual screening success rates improved consistently as more query molecules were used (Strategy 1). While the baseline 2D method, using a single query molecule, obtained an average AUC of 0.68, EF1% of 19.96 and SRR1% of 0.20, the 2D method, using a set of five query molecules, obtained an average AUC of 0.82, EF1% of 44.59 and SRR1% of 0.42. Therefore, the use of multiple query molecules for screening is clearly recommended (the more the merrier).

A third key observation is that the overlap between the active scaffolds ranked early in the hit lists (top 1%) obtained with the 2D method and the 3D method was as low as 5%. Given the fact that early enrichment was good but the overlap of active compounds among the top ranks was low, it is reasonable to expect that the merging of the hit lists obtained with the 2D method and the 3D method by (balanced) parallel selection (Strategy 2) would improve virtual screening performance. This was indeed the case: balanced, parallel selection yielded an average AUC of 0.70, EF1% of 28.16 and SRR1% of 0.27, while the 2D method yielded (as mentioned above) an average AUC of 0.68, EF1% of 19.96 and SRR1% of 0.20. By merging multi-query screening (Strategy 1) and (balanced), parallel selection (Strategy 2), further improvement of virtual screening was achieved: this integrated approach (Strategy 3) yielded an average AUC of 0.84, EF1% of 53.82 and SRR1% of 0.50.

In summary, this study found that, when considering only performance, virtual screening methods based on 3D molecular shape representations seem to have no edge over rapid methods based on 2D molecular fingerprints. That said, a number of features of (some) 3D methods can prove extremely valuable in virtual screening, such as the capacity to produce intuitive alignments for visual inspection and the option to customize molecular shape and pharmacophoric features. However, the main point to make here is not on the advantages and disadvantages of the 2D and 3D methods for similarity-based screening, but on the boost in virtual screening performance that is yielded consistently by the combination of both methods. Based on the results obtained in this work, we give a clear recommendation for the integration of 2D and 3D methods, by use of a (balanced) parallel selection strategy, ideally combined with multi-query screening.

## Figures and Tables

**Table 1:** Composition of the Prepared Data Set for Virtual Screening.

ChEMBL ID	Target Name	No. Active Compounds ^1^	No. Confirmed Inactive Compounds ^1^
CHEMBL1792	Somatostatin receptor 5	387	6
CHEMBL1844	Macrophage colony stimulating factor receptor	662	5
CHEMBL1862	Tyrosine-protein kinase ABL	901	115
CHEMBL1889	Vasopressin V1a receptor	515	28
CHEMBL1946	Melatonin receptor 1B	452	0
CHEMBL1952	Thymidylate synthase	165	54
CHEMBL1957	Insulin-like growth factor I receptor	1767	92
CHEMBL1974	Tyrosine-protein kinase receptor FLT3	1139	56
CHEMBL1983	Serotonin 1d (5-HT1d) receptor	475	3
CHEMBL1991	Inhibitor of nuclear factor κ B kinase β subunit	893	134
CHEMBL2034	Glucocorticoid receptor	1216	40
CHEMBL2049	Oxytocin receptor	378	36
CHEMBL208	Progesterone receptor	859	26
CHEMBL210	β_2_-adrenergic receptor	633	202
CHEMBL2276	c-Jun N-terminal kinase I	753	44
CHEMBL230	Cyclooxygenase-2	1108	407
CHEMBL2337	Glycine transporter 1	462	32
CHEMBL234	Dopamine receptor D3	2556	20
CHEMBL2373	C5a anaphylatoxin chemotactic receptor	74	23
CHEMBL2414	C-C chemokine receptor type 4	254	28
CHEMBL2434	Interleukin-8 receptor B	614	15
CHEMBL245	Muscarinic acetylcholine receptor M3	963	60
CHEMBL254	Phosphodiesterase 4A	369	20
CHEMBL2568	Liver glycogen phosphorylase	283	36
CHEMBL267	Tyrosine-protein kinase SRC	829	120
CHEMBL286	Renin	1028	44
CHEMBL288	Phosphodiesterase 4D	475	93
CHEMBL2954	Cathepsin S	1202	74
CHEMBL3242	Carbonic anhydrase XII	2109	57
CHEMBL3397	Cytochrome P450 2C9	1763	1159
CHEMBL3764	Urotensin II receptor	248	0
CHEMBL3772	Metabotropic glutamate receptor 1	582	30
CHEMBL3785	Nicotinic acid receptor 1	259	13
CHEMBL3837	Cathepsin L	947	395
CHEMBL4015	C-C chemokine receptor type 2	960	65
CHEMBL4072	Cathepsin B	573	295
CHEMBL4234	Estradiol 17-β-dehydrogenase 3	133	2
CHEMBL4296	Sodium channel protein type IX α subunit	1852	106
CHEMBL4306	Voltage-gated potassium channel subunit Kv1.5	431	43
CHEMBL4333	Sphingosine 1-phosphate receptor Edg-1	1627	118
CHEMBL4561	Neuropeptide Y receptor type 5	577	1
CHEMBL4616	Ghrelin receptor	906	17
CHEMBL4618	Leukotriene A4 hydrolase	230	42
CHEMBL4722	Serine/threonine-protein kinase Aurora-A	1247	145
CHEMBL4777	Neuropeptide Y receptor type 1	271	136
CHEMBL4792	Orexin receptor 2	1150	0
CHEMBL4805	P2X purinoceptor 7	1456	12
CHEMBL4822	β-secretase 1	2559	299
CHEMBL5071	G protein-coupled receptor 44	1799	53
CHEMBL5145	Serine/threonine-protein kinase B-raf	1579	166

^1^ To this number of confirmed inactive compounds, 94,685 presumed inactive compounds were added (these 94,685 compounds resulted from a random sample of 100,000 presumed inactive compounds that were extracted from the Enamine HTS Collection and preprocessed according to the protocol reported in Section 3.1).

**Table 2:** Performance of the Investigated Virtual Screening Approaches and Strategies.

Method	Mean AUC ^1^	Mean Stdev ^2^	EF1%	Stdev EF1%	EF10%	Stdev EF10%	SRR 1%	Stdev SRR1%	SRR 10%	Stdev SRR10%
2D single query	0.68	0.10	19.96	10.87	3.69	1.43	0.20	0.10	0.38	0.14
3D single query	0.54	0.13	17.52	10.54	2.87	1.38	0.17	0.10	0.29	0.13
2D five queries	0.82	0.05	44.59	8.48	6.23	0.88	0.42	0.08	0.61	0.08
3D five queries	0.69	0.07	39.48	8.39	5.20	0.93	0.36	0.08	0.50	0.09
Balanced, parallel selection	0.70	0.10	28.16	11.14	4.44	1.35	0.27	0.10	0.45	0.13
Integrated approach	0.84	0.04	53.82	6.57	6.97	0.69	0.50	0.07	0.67	0.07

^1^ AUC averaged across all targets, where each target is represented by a single AUC value that itself is an average obtained from 30 screening runs with different query molecules. ^2^ Standard deviation was averaged across the 50 target-specific standard deviations (where the standard deviations for the individual targets were derived from 30 screening runs with different query molecules).

**Table 3:** Number of Targets on which the 2D Method Outperformed the 3D Method.

Query Set Size	No. Targets on Which the 2D Approach Outperformed the 3D Approach with Respect to the	
	EF1%	SRR1%
1	37	34
2	36	37
3	39	39
4	38	39
5	39	40

**Table 4:** Scaffold Recovery Rates for the Top 1% and Top 10% Ranks, averaged across the 50 Targets.

SRR	2D Approach	3D Approach	Proportion of Scaffolds of Active Compounds Identified Exclusively by the		Proportion of Scaffolds of Active Compounds Identified by	
			2D Approach	3D Approach	Both Approaches	at Least one of the Two Approaches
SRR1%	0.20	0.17	0.14	0.12	0.05	0.31
SRR10%	0.38	0.29	0.24	0.15	0.14	0.53

## Data Availability

This work is based exclusively on public data from the ChEMBL database [43] and the Enamine HTS Collection [36].

## Supplementary Materials

The following supporting information can be downloaded at: https://www.mdpi.com/article/10.3390/ijms23147747/s1.
