# Supplementary material for: Maximizing the Performance of Similarity-Based Virtual Screening Methods by Generating Synergy from the Integration of 2D and 3D Approaches
Source: Int J Mol Sci. 2022 Jul 13;23(14):7747. doi: 10.3390/ijms23147747 (PMC9322642; doi:10.3390/ijms23147747)
Supplement: Supplementary file 1 [file ijms-23-07747-s001.zip › ijms-1795436-supplementary.pdf]

Supporting Information

Maximizing the performance of similarity-based virtual screening methods by generating synergy from the integration of 2D and 3D approaches

Ningning Fan [1], Steffen Hirte [2,3] and Johannes Kirchmair [1,2*]

[1] Center for Bioinformatics (ZBH), Department of Informatics, Faculty of Mathematics, Informatics and Natural Sciences, Universität Hamburg, 20146 Hamburg, Germany; fan@zbh.uni-hamburg.de

[2] Division of Pharmaceutical Chemistry, Department of Pharmaceutical Sciences, Faculty of Life Sciences, University of Vienna, 1090 Vienna, Austria; steffen.hirte@univie.ac.at and johannes.kirchmair@univie.ac.at

[3] Vienna Doctoral School of Pharmaceutical, Nutritional and Sport Sciences (PhaNuSpo), University of Vienna, 1090 Vienna, Austria

* Correspondence: johannes.kirchmair@univie.ac.at; Tel.: +43-1-4277-55104

Table of contents

Table S1. Mean AUC Values and Standard Deviations Obtained with Different Methods for the Individual Targets. 3

Table S2. Mean EF1% Values and Standard Deviations Obtained with Different Methods for the Individual Targets. 5

Table S3. Overall EFs and SRRs Obtained with Different Methods Using A Single Active Compound as Query. 7

Table S4. Mean EFs, SRRs and Standard Deviations Obtained with the 2D Fingerprint-based Approach for the Individual Targets. 8

Table S5. Mean EFs, SRRs and Standard Deviations Obtained with the 3D Shape-based Approach for the Individual Targets. 10

Table S6. Mean SRR1% Values and Their Standard Deviations Obtained with Different Methods for the Individual Targets. 12

Table S7. Median EFs, SRRs and Standard Deviations Obtained by the Different Approaches for the Individual Targets. 15

Table S8. Overall AUC values, EFs, SRRs and Standard Deviations Obtained with Different Methods Using Query Sets of Different Size. 17

Table S9. Mean AUC Values and Standard Deviations Obtained by the 2D Fingerprint-based Approach for the Individual Targets Using Query Sets of Different Size. 18

Table S10. Mean AUC Values and Standard Deviations Obtained by the 3D Shape-based Approach for the Individual Targets Using Query Sets of Different Size. 20

Table S11. Mean EF1% values and Standard Deviations Obtained by the 2D Fingerprint-based Approach for the Individual Targets Using Query Sets of Different Size. 22

Table S12. Mean EF10% Values and Standard Deviations Obtained with the 2D Fingerprint-based Approach for the Individual Targets Using Query Sets of Different Size. 25

Table S13. Mean EF1% Values and Standard Deviations Obtained with the 3D Shape-based Approach for the Individual Targets Using Query Sets of Different Size. 27

Table S14. Mean EF10% Values and Standard Deviations Obtained with the 3D Shape-based Approach for the Individual Targets Using Query Sets of Different Size. 29

Table S15. Mean SRR1% values and Standard Deviations Obtained with the 2D Fingerprint-based Approach for the Individual Targets Using Query Sets of Different Size. 31

Table S16. Mean SRR10% values and Standard Deviations Obtained with the 2D Fingerprint-based Approach for the Individual Targets Using Query Sets of Different Size. 34

Table S17. Mean SRR1% values and Standard Deviations Obtained with the 3D Shape-based Approach for the Individual Targets Using Query Sets of Different Size. 36

Table S18. Mean SRR10% Values and Standard Deviations Obtained by the 3D Shape-based Approach for the Individual Targets Using Query Sets of Different Size. 38

Table S19. Overall EFs and SRRs Obtained with the Parallel Selection Approach at Different Ratios of Hits Selected from the Hit Lists. 41

Table S20. Overall AUC Values, EFs and SRRs Obtained with the Integrated Approach. 41

Table S21. Mean AUC Values and Standard Deviations Obtained by the Integrated Approach for the Individual Targets Using Query Sets of Different Size. 42

Table S22. Mean EF1% Values and Standard Deviations Obtained with the Integrated Approach for the Individual Targets Using Query Sets of Different Size...............................44

Table S23. Mean EF10% Values and Standard Deviations Obtained with the Integrated Approach for the Individual Targets Using Query Sets of Different Size...............................46

Table S24. Mean SRR1% Values and Standard Deviations Obtained with the Integrated Approach for the Individual Targets Using Query Sets of Different Size...............................49

Table S25. Mean SRR10% Values and Standard Deviations Values Obtained with the Integrated Approach for the Individual Targets Using Query Sets of Different Size. 51

Table S1. Mean AUC Values and Standard Deviations Obtained with Different Methods for the Individual Targets.

Target name	2D single query[1]	Stdev	3D single query[1]	Stdev	2D five queries[1]	Stdev	3D five queries[1]	Stdev	Balanced, parallel selection[1]	Stdev	Integrated approach[2]	Stdev
Somatostatin receptor 5	0.77	0.11	0.71	0.18	0.92	0.05	0.88	0.03	0.84	0.09	0.94	0.04
Macrophage colony stimulating factor receptor	0.65	0.07	0.48	0.15	0.80	0.05	0.66	0.10	0.66	0.09	0.85	0.06
Tyrosine-protein kinase ABL	0.74	0.11	0.57	0.12	0.83	0.03	0.74	0.06	0.76	0.09	0.85	0.02
Vasopressin V1a receptor	0.62	0.07	0.51	0.13	0.75	0.06	0.60	0.06	0.64	0.09	0.78	0.05
Melatonin receptor 1B	0.87	0.08	0.76	0.09	0.97	0.01	0.88	0.03	0.92	0.06	0.99	0.01
Thymidylate synthase	0.65	0.15	0.58	0.13	0.85	0.05	0.78	0.07	0.70	0.11	0.89	0.04
Insulin-like growth factor I receptor	0.83	0.07	0.65	0.12	0.90	0.02	0.72	0.08	0.84	0.05	0.89	0.03
Tyrosine-protein kinase receptor FLT3	0.58	0.10	0.43	0.14	0.68	0.07	0.48	0.07	0.57	0.09	0.70	0.07
Serotonin 1d (5-HT1d) receptor	0.74	0.11	0.61	0.18	0.88	0.02	0.76	0.07	0.78	0.08	0.89	0.02
Inhibitor of nuclear factor κ B kinase β subunit	0.74	0.13	0.59	0.21	0.84	0.03	0.81	0.03	0.79	0.08	0.85	0.03
Glucocorticoid receptor	0.57	0.14	0.44	0.12	0.72	0.06	0.57	0.07	0.59	0.13	0.77	0.05
Oxytocin receptor	0.58	0.09	0.50	0.09	0.80	0.09	0.71	0.10	0.65	0.10	0.87	0.06
Progesterone receptor	0.57	0.12	0.54	0.12	0.71	0.09	0.69	0.08	0.62	0.11	0.78	0.09
β_2-adrenergic receptor	0.88	0.09	0.59	0.13	0.94	0.01	0.70	0.07	0.88	0.08	0.93	0.01
c-Jun N-terminal kinase I	0.71	0.07	0.50	0.11	0.85	0.06	0.72	0.06	0.70	0.09	0.85	0.06
Cyclooxygenase-2	0.63	0.16	0.50	0.15	0.75	0.04	0.68	0.08	0.66	0.13	0.78	0.03
Glycine transporter 1	0.64	0.07	0.52	0.09	0.83	0.06	0.75	0.06	0.66	0.06	0.90	0.04
Dopamine receptor D3	0.74	0.11	0.64	0.22	0.84	0.04	0.75	0.09	0.79	0.08	0.85	0.03
C5a anaphylatoxin chemotactic receptor	0.71	0.13	0.51	0.15	0.95	0.03	0.66	0.09	0.78	0.18	0.97	0.02
C-C chemokine receptor type 4	0.66	0.16	0.51	0.12	0.84	0.07	0.76	0.07	0.68	0.14	0.88	0.04
Interleukin-8 receptor B	0.62	0.11	0.54	0.08	0.83	0.06	0.77	0.06	0.67	0.09	0.87	0.06
Muscarinic acetylcholine receptor M3	0.65	0.10	0.52	0.14	0.76	0.04	0.62	0.07	0.67	0.08	0.80	0.03

Phosphodiesterase 4A	0.52	0.10	0.51	0.08	0.70	0.06	0.65	0.06	0.58	0.09	0.76	0.07
Liver glycogen phosphorylase	0.81	0.06	0.62	0.11	0.94	0.02	0.84	0.10	0.82	0.06	0.94	0.02
Tyrosine-protein kinase SRC	0.68	0.11	0.55	0.14	0.79	0.04	0.64	0.06	0.71	0.12	0.82	0.04
Renin	0.76	0.08	0.51	0.16	0.88	0.04	0.63	0.06	0.76	0.07	0.88	0.04
Phosphodiesterase 4D	0.57	0.09	0.51	0.12	0.71	0.07	0.61	0.10	0.61	0.09	0.75	0.06
Cathepsin S	0.68	0.07	0.43	0.13	0.81	0.05	0.51	0.09	0.65	0.08	0.80	0.06
Carbonic anhydrase XII	0.71	0.14	0.44	0.10	0.82	0.03	0.55	0.07	0.68	0.14	0.80	0.04
Cytochrome P450 2C9	0.48	0.08	0.36	0.06	0.52	0.05	0.39	0.04	0.45	0.07	0.50	0.04
Urotensin II receptor	0.75	0.07	0.45	0.12	0.85	0.03	0.69	0.09	0.72	0.07	0.86	0.03
Metabotropic glutamate receptor 1	0.62	0.13	0.69	0.14	0.82	0.09	0.92	0.03	0.74	0.13	0.92	0.02
Nicotinic acid receptor 1	0.60	0.09	0.61	0.06	0.90	0.11	0.92	0.09	0.69	0.11	0.97	0.06
Cathepsin L	0.64	0.09	0.41	0.14	0.80	0.04	0.50	0.10	0.62	0.11	0.80	0.05
C-C chemokine receptor type 2	0.79	0.13	0.58	0.19	0.91	0.02	0.75	0.07	0.79	0.12	0.91	0.02
Cathepsin B	0.67	0.12	0.42	0.12	0.81	0.04	0.55	0.08	0.65	0.12	0.80	0.05
Estradiol 17-β-dehydrogenase 3	0.63	0.12	0.59	0.09	0.89	0.07	0.87	0.08	0.72	0.11	0.94	0.01
Sodium channel protein type IX α subunit	0.75	0.19	0.64	0.20	0.88	0.02	0.81	0.04	0.81	0.11	0.88	0.02
Voltage-gated potassium channel subunit Kv1.5	0.52	0.09	0.45	0.12	0.67	0.08	0.58	0.08	0.55	0.11	0.74	0.09
Sphingosine 1-phosphate receptor Edg-1	0.67	0.11	0.67	0.22	0.82	0.04	0.76	0.10	0.75	0.12	0.86	0.04
Neuropeptide Y receptor type 5	0.63	0.12	0.56	0.20	0.83	0.10	0.81	0.08	0.69	0.13	0.90	0.06
Ghrelin receptor	0.60	0.09	0.42	0.14	0.75	0.07	0.51	0.15	0.58	0.11	0.75	0.09
Leukotriene A4 hydrolase	0.83	0.13	0.78	0.15	0.95	0.03	0.90	0.04	0.89	0.09	0.97	0.03
Serine/threonine-protein kinase Aurora-A	0.68	0.09	0.47	0.15	0.80	0.04	0.56	0.08	0.66	0.11	0.79	0.04
Neuropeptide Y receptor type 1	0.47	0.08	0.43	0.16	0.56	0.07	0.45	0.08	0.50	0.11	0.60	0.07
Orexin receptor 2	0.67	0.09	0.64	0.10	0.80	0.05	0.76	0.03	0.70	0.08	0.84	0.03
P2X purinoceptor 7	0.73	0.06	0.62	0.05	0.84	0.03	0.77	0.03	0.75	0.05	0.85	0.02
β-secretase 1	0.64	0.12	0.42	0.07	0.79	0.07	0.56	0.05	0.62	0.08	0.76	0.06

G protein-coupled receptor 44	0.79	0.08	0.64	0.13	0.89	0.03	0.81	0.06	0.80	0.08	0.92	0.02
Serine/threonine-protein kinase B-raf	0.72	0.09	0.49	0.11	0.86	0.03	0.63	0.08	0.71	0.08	0.85	0.04
Average	0.68	0.10	0.54	0.13	0.82	0.05	0.69	0.07	0.70	0.10	0.84	0.04

[1] Reported values are averages obtained from 30 screening runs with different (sets of) query molecules.

[2] Using sets of five query molecules in combination with balanced, parallel selection.

Table S2. Mean EF1% Values and Standard Deviations Obtained with Different Methods for the Individual Targets.

Target name	2D single query[1]	Stdev	3D single query[1]	Stdev	2D five queries[1]	Stdev	3D five queries[1]	Stdev	Balanced, parallel selection[1]	Stdev	Integrated approach[2]	Stdev
Somatostatin receptor 5	36.86	21.21	34.50	25.23	74.01	13.49	72.34	10.57	56.52	22.98	83.61	3.94
Macrophage colony stimulating factor receptor	14.49	8.80	14.47	8.65	43.57	11.60	45.30	12.06	23.33	8.01	61.15	9.37
Tyrosine-protein kinase ABL	23.34	16.02	25.15	18.19	51.57	7.91	51.42	6.21	35.86	16.36	60.09	4.42
Vasopressin V1a receptor	10.57	4.66	11.04	5.76	29.50	6.00	26.76	6.16	17.11	5.06	41.71	5.02
Melatonin receptor 1B	36.60	16.42	39.00	16.08	62.94	5.22	64.52	5.47	52.21	15.74	79.67	4.22
Thymidylate synthase	20.57	16.38	19.13	16.87	53.93	9.51	51.64	13.06	32.08	17.72	70.44	9.82
Insulin-like growth factor I receptor	25.13	8.93	22.53	9.35	47.11	6.31	37.83	7.17	33.87	9.57	49.54	4.16
Tyrosine-protein kinase receptor FLT3	7.74	4.63	6.41	3.98	23.09	7.09	17.14	5.57	11.09	5.77	31.03	6.45
Serotonin 1d (5-HT1d) receptor	25.34	19.85	20.05	15.01	55.66	4.87	42.95	12.22	35.09	17.13	61.63	5.82
Inhibitor of nuclear factor κ B kinase β subunit	40.57	24.80	38.04	26.29	62.34	3.06	64.21	4.72	52.96	13.92	67.35	5.04
Glucocorticoid receptor	14.86	9.09	9.13	4.34	33.79	8.06	26.71	5.69	19.56	8.55	43.98	5.39
Oxytocin receptor	17.42	10.18	17.29	9.96	52.69	11.95	52.12	12.94	29.68	13.55	67.23	9.04
Progesterone receptor	11.83	7.16	11.55	9.17	30.71	10.29	25.40	9.77	18.38	8.70	39.19	11.32
β_2-adrenergic receptor	43.84	18.69	22.13	11.34	67.28	9.91	36.86	9.17	46.18	14.73	66.39	6.90
c-Jun N-terminal kinase I	19.54	9.80	19.43	11.99	44.68	9.33	42.42	6.57	29.63	11.87	52.15	8.95
Cyclooxygenase-2	14.98	11.73	13.15	13.94	25.52	7.37	33.20	9.65	19.76	11.69	38.06	6.64
Glycine transporter 1	16.51	7.15	15.58	7.64	48.28	10.25	46.47	7.72	27.14	7.80	66.68	7.79

Dopamine receptor D3	15.46	7.33	16.83	11.23	31.03	4.13	26.35	7.10	21.56	7.79	32.69	3.33
C5a anaphylatoxin chemotactic receptor	38.73	14.09	28.31	15.31	82.24	13.28	55.46	9.78	55.14	21.96	89.90	6.33
C-C chemokine receptor type 4	27.13	19.99	21.00	15.80	60.47	12.18	55.52	10.66	37.85	17.32	74.51	8.87
Interleukin-8 receptor B	17.20	7.20	19.12	8.73	44.48	8.89	50.22	11.46	28.31	7.99	63.29	10.74
Muscarinic acetylcholine receptor M3	12.38	9.28	14.58	12.48	30.79	8.82	29.10	9.99	20.36	10.67	42.06	7.72
Phosphodiesterase 4A	10.69	5.68	9.80	4.21	34.27	8.13	28.94	7.46	17.28	6.65	45.50	10.43
Liver glycogen phosphorylase	31.39	9.85	26.67	9.01	74.29	11.90	65.31	14.71	44.03	13.62	85.95	5.06
Tyrosine-protein kinase SRC	16.18	9.20	14.02	8.92	36.03	8.07	28.95	6.96	23.76	11.17	44.72	6.76
Renin	20.32	6.55	12.24	4.86	45.67	9.51	30.36	5.47	25.45	6.25	52.77	6.57
Phosphodiesterase 4D	9.53	7.39	7.30	6.72	27.28	10.25	22.22	11.06	14.08	9.69	39.15	10.58
Cathepsin S	12.29	5.29	7.41	3.95	29.49	5.85	18.67	3.90	15.22	6.02	35.67	5.04
Carbonic anhydrase XII	14.17	8.90	5.17	4.20	19.92	4.82	9.50	4.15	12.07	5.82	18.46	3.61
Cytochrome P450 2C9	3.86	3.17	3.08	3.36	9.89	4.42	9.67	4.05	5.53	3.47	13.94	3.12
Urotensin II receptor	22.19	11.59	14.70	6.98	53.42	10.44	42.47	9.01	29.76	10.85	64.88	6.47
Metabotropic glutamate receptor 1	25.07	12.69	26.38	12.84	62.08	12.78	68.24	11.35	40.65	16.72	76.04	5.35
Nicotinic acid receptor 1	31.13	10.99	34.37	13.30	70.37	13.51	76.58	12.02	46.81	14.60	86.18	8.91
Cathepsin L	13.62	8.09	8.37	5.85	33.46	7.03	20.40	5.76	16.73	7.04	38.29	6.05
C-C chemokine receptor type 2	28.75	15.67	15.79	10.80	54.25	6.42	35.10	7.95	32.09	13.30	55.98	5.51
Cathepsin B	16.62	8.24	10.61	6.14	37.48	7.27	28.07	7.82	20.05	8.33	44.54	6.17
Estradiol 17-β-dehydrogenase 3	28.53	11.70	25.67	11.17	79.70	14.30	70.17	12.06	48.35	14.52	88.41	1.69
Sodium channel protein type IX α subunit	29.39	17.82	30.55	20.98	47.92	2.93	49.75	1.66	33.24	13.24	50.22	0.60
Voltage-gated potassium channel subunit Kv1.5	8.85	4.64	8.68	4.69	30.04	6.95	28.68	7.10	15.51	6.42	43.36	8.71
Sphingosine 1-phosphate receptor Edg-1	17.17	11.37	20.52	13.34	34.97	8.28	29.83	13.88	25.45	11.62	41.79	9.03
Neuropeptide Y receptor type 5	17.64	10.34	17.42	9.71	46.98	11.15	47.58	9.00	27.57	11.17	63.76	8.64
Ghrelin receptor	12.22	11.85	10.14	8.93	40.20	10.94	27.51	8.55	18.42	11.15	46.57	10.54
Leukotriene A4 hydrolase	30.66	11.99	31.24	15.95	66.88	10.17	60.37	17.65	45.28	15.51	79.75	12.59

Serine/threonine-protein kinase Aurora-A	14.06	12.73	11.43	11.54	34.13	9.81	26.61	6.94	18.02	12.63	38.99	5.14
Neuropeptide Y receptor type 1	8.54	7.83	7.28	6.79	26.73	9.40	16.19	6.76	13.35	8.68	34.54	8.19
Orexin receptor 2	16.29	11.37	19.99	13.17	41.89	5.88	44.86	8.11	27.16	12.42	55.07	4.93
P2X purinoceptor 7	18.87	8.31	15.63	6.11	42.36	6.31	39.31	4.75	25.54	8.74	49.22	5.18
β-secretase 1	18.09	10.16	14.82	11.26	33.37	4.63	32.60	5.50	20.98	6.77	35.06	2.32
G protein-coupled receptor 44	15.57	8.34	17.92	9.30	29.93	5.38	36.61	6.60	22.55	7.87	41.52	4.80
Serine/threonine-protein kinase B-raf	15.46	8.24	10.28	5.31	30.86	7.93	25.62	5.79	19.52	8.02	38.12	5.13
Average	19.96	10.87	17.52	10.53	44.59	8.48	39.48	8.39	28.16	11.14	53.82	6.57

[1] Reported values are averages obtained from 30 screening runs with different (sets of) query molecules.
[2] Using sets of five query molecules in combination with balanced, parallel selection.

Table S3. Overall EFs and SRRs Obtained with Different Methods Using A Single Active Compound as Query.

Method	Aggregate function	Enrichment factor (EF)				Scaffold recovery rate (SRR)			
		1%	3%	5%	10%	1%	3%	5%	10%
2D single query	mean	19.96	8.64	5.95	3.69	0.20	0.26	0.30	0.38
	median	19.57	8.75	6.13	3.89	0.20	0.27	0.32	0.40
3D single query	mean	17.52	7.20	4.82	2.87	0.17	0.21	0.24	0.29
	median	17.37	7.38	4.98	2.99	0.17	0.22	0.25	0.31

Table S4. Mean EFs, SRRs and Standard Deviations Obtained with the 2D Fingerprint-based Approach for the Individual Targets.

Target Name	Enrichment factor						Scaffold recovery rate					
	top-3%	Stdev	top-5%	Stdev	top-10%	Stdev	top-3%	Stdev	top-5%	Stdev	top-10%	Stdev
Somatostatin receptor 5	14.45	7.16	9.37	4.33	5.28	2.21	0.38	0.15	0.42	0.15	0.48	0.15
Macrophage colony stimulating factor receptor	5.70	2.94	3.94	1.82	2.63	1.06	0.17	0.10	0.21	0.10	0.28	0.12
Tyrosine-protein kinase ABL	9.56	5.41	6.56	3.36	4.20	1.79	0.26	0.12	0.30	0.13	0.39	0.15
Vasopressin V1a receptor	4.74	2.30	3.37	1.51	2.33	0.89	0.15	0.08	0.18	0.09	0.26	0.10
Melatonin receptor 1B	16.75	6.13	11.25	3.87	6.57	1.97	0.56	0.18	0.61	0.17	0.71	0.17
Thymidylate synthase	8.28	5.90	5.63	3.72	3.45	1.92	0.23	0.11	0.27	0.13	0.35	0.14
Insulin-like growth factor I receptor	12.18	3.65	8.67	2.37	5.52	1.29	0.40	0.12	0.46	0.13	0.58	0.13
Tyrosine-protein kinase receptor FLT3	4.02	2.17	3.03	1.49	2.20	0.92	0.14	0.08	0.17	0.09	0.25	0.11
Serotonin 1d (5-HT1d) receptor	10.60	6.79	7.20	4.20	4.29	2.13	0.30	0.16	0.34	0.16	0.42	0.17
Inhibitor of nuclear factor κ B kinase β subunit	14.74	8.07	9.24	4.77	5.05	2.25	0.36	0.17	0.39	0.17	0.45	0.16
Glucocorticoid receptor	6.56	3.73	4.49	2.50	2.76	1.42	0.22	0.14	0.25	0.15	0.31	0.17
Oxytocin receptor	6.57	3.30	4.34	1.92	2.64	1.03	0.17	0.06	0.19	0.06	0.26	0.08
Progesterone receptor	5.07	3.03	3.57	1.99	2.36	1.17	0.18	0.11	0.21	0.11	0.28	0.13
β_2-adrenergic receptor	19.53	6.51	12.99	3.74	7.33	1.75	0.55	0.19	0.61	0.17	0.70	0.16
c-Jun N-terminal kinase I	8.40	3.62	5.83	2.34	3.67	1.27	0.27	0.12	0.32	0.13	0.39	0.14
Cyclooxygenase-2	7.27	5.35	5.12	3.54	3.30	2.02	0.24	0.16	0.28	0.17	0.36	0.18
Glycine transporter 1	6.54	2.55	4.45	1.56	2.84	0.82	0.20	0.08	0.24	0.08	0.31	0.08
Dopamine receptor D3	7.90	3.38	5.93	2.30	4.10	1.32	0.26	0.10	0.32	0.12	0.44	0.14
C5a anaphylatoxin chemotactic receptor	14.12	4.54	8.88	2.76	4.82	1.48	0.42	0.15	0.45	0.16	0.49	0.16
C-C chemokine receptor type 4	10.08	7.15	6.47	4.43	3.66	2.29	0.23	0.11	0.26	0.13	0.32	0.15

Interleukin-8 receptor B	7.38	3.22	5.05	2.21	3.12	1.26	0.22	0.09	0.26	0.10	0.32	0.12
Muscarinic acetylcholine receptor M3	5.71	3.81	4.13	2.49	2.79	1.36	0.18	0.13	0.22	0.14	0.29	0.15
Phosphodiesterase 4A	4.41	2.13	3.05	1.39	2.01	0.79	0.15	0.08	0.18	0.09	0.24	0.10
Liver glycogen phosphorylase	12.25	3.44	8.24	2.09	5.05	1.24	0.35	0.12	0.39	0.12	0.48	0.13
Tyrosine-protein kinase SRC	7.58	3.73	5.50	2.45	3.65	1.42	0.25	0.13	0.30	0.14	0.39	0.16
Renin	9.31	2.67	6.65	1.79	4.35	1.04	0.34	0.10	0.40	0.11	0.51	0.12
Phosphodiesterase 4D	4.42	2.89	3.20	1.80	2.26	0.99	0.14	0.08	0.18	0.09	0.25	0.11
Cathepsin S	6.37	2.29	4.77	1.56	3.22	0.92	0.21	0.08	0.27	0.09	0.36	0.10
Carbonic anhydrase XII	9.08	5.51	6.82	3.84	4.44	2.12	0.30	0.17	0.36	0.20	0.47	0.21
Cytochrome P450 2C9	1.99	1.36	1.56	0.95	1.23	0.63	0.05	0.03	0.07	0.04	0.12	0.05
Urotensin II receptor	9.39	3.79	6.48	2.34	4.19	1.21	0.29	0.11	0.34	0.11	0.45	0.12
Metabotropic glutamate receptor 1	8.89	4.23	5.60	2.48	3.14	1.28	0.26	0.09	0.27	0.09	0.33	0.10
Nicotinic acid receptor 1	11.47	4.01	7.19	2.46	3.93	1.25	0.37	0.14	0.39	0.14	0.43	0.15
Cathepsin L	6.70	3.61	4.91	2.40	3.25	1.38	0.24	0.11	0.29	0.13	0.38	0.15
C-C chemokine receptor type 2	13.22	6.51	9.28	4.29	5.63	2.21	0.39	0.18	0.46	0.20	0.56	0.20
Cathepsin B	8.28	3.80	5.93	2.56	3.74	1.41	0.27	0.12	0.32	0.13	0.41	0.14
Estradiol 17-β-dehydrogenase 3	9.92	4.10	6.25	2.57	3.49	1.32	0.29	0.11	0.32	0.14	0.38	0.16
Sodium channel protein type IX α subunit	13.91	7.84	9.21	4.90	5.22	2.51	0.40	0.22	0.45	0.23	0.52	0.24
Voltage-gated potassium channel subunit Kv1.5	3.79	1.81	2.72	1.26	1.77	0.78	0.12	0.06	0.15	0.07	0.20	0.09
Sphingosine 1-phosphate receptor Edg-1	8.62	5.49	5.92	3.59	3.59	1.86	0.25	0.15	0.30	0.16	0.37	0.16
Neuropeptide Y receptor type 5	7.31	3.73	5.10	2.41	3.19	1.36	0.24	0.13	0.28	0.14	0.36	0.16
Ghrelin receptor	5.12	4.49	3.55	2.82	2.23	1.40	0.16	0.12	0.18	0.13	0.24	0.12
Leukotriene A4 hydrolase	13.79	5.19	9.76	3.44	6.18	1.98	0.43	0.20	0.50	0.21	0.62	0.23
Serine/threonine-protein kinase Aurora-A	6.50	4.45	4.71	2.70	3.23	1.36	0.19	0.11	0.24	0.12	0.33	0.12
Neuropeptide Y receptor type 1	3.12	2.56	2.08	1.50	1.30	0.77	0.09	0.06	0.10	0.06	0.14	0.07
Orexin receptor 2	7.19	4.44	5.05	2.88	3.24	1.52	0.22	0.12	0.26	0.13	0.34	0.14

P2X purinoceptor 7	8.87	3.11	6.31	1.87	4.07	0.97	0.24	0.07	0.29	0.07	0.38	0.08
β-secretase 1	8.35	4.27	5.79	2.81	3.54	1.53	0.22	0.10	0.26	0.11	0.33	0.12
G protein-coupled receptor 44	8.80	4.05	6.73	2.71	4.63	1.52	0.29	0.12	0.37	0.13	0.50	0.14
Serine/threonine-protein kinase B-raf	7.35	3.29	5.38	2.20	3.63	1.30	0.23	0.08	0.28	0.10	0.38	0.11
Average	8.64	4.19	5.95	2.67	3.69	1.43	0.26	0.12	0.30	0.13	0.38	0.14

Table S5. Mean EFs, SRRs and Standard Deviations Obtained with the 3D Shape-based Approach for the Individual Targets.

Target Name	Enrichment factor						Scaffold recovery rate					
	top-3%	Stdev	top-5%	Stdev	top-10%	Stdev	top-3%	Stdev	top-5%	Stdev	top-10%	Stdev
Somatostatin receptor 5	13.61	9.05	8.85	5.58	4.95	2.84	0.35	0.21	0.38	0.21	0.44	0.22
Macrophage colony stimulating factor receptor	5.59	3.02	3.75	1.93	2.25	1.11	0.16	0.09	0.18	0.10	0.22	0.12
Tyrosine-protein kinase ABL	9.74	6.24	6.30	3.73	3.54	1.78	0.24	0.12	0.26	0.12	0.31	0.12
Vasopressin V1a receptor	5.16	2.76	3.67	1.95	2.33	1.20	0.16	0.09	0.18	0.10	0.24	0.13
Melatonin receptor 1B	16.05	5.54	10.49	3.32	5.89	1.57	0.45	0.13	0.49	0.13	0.55	0.12
Thymidylate synthase	7.52	5.79	4.93	3.58	2.91	1.77	0.21	0.11	0.24	0.13	0.28	0.14
Insulin-like growth factor I receptor	9.74	3.97	6.64	2.57	3.97	1.34	0.31	0.10	0.36	0.10	0.42	0.10
Tyrosine-protein kinase receptor FLT3	2.94	1.61	2.14	1.11	1.46	0.75	0.09	0.06	0.12	0.07	0.17	0.10
Serotonin 1d (5-HT1d) receptor	8.71	5.75	5.91	3.63	3.57	1.92	0.23	0.13	0.26	0.14	0.33	0.15
Inhibitor of nuclear factor κ B kinase β subunit	13.18	8.78	8.12	5.27	4.30	2.62	0.32	0.19	0.34	0.19	0.37	0.19
Glucocorticoid receptor	3.83	1.87	2.65	1.29	1.74	0.82	0.13	0.08	0.15	0.09	0.19	0.10
Oxytocin receptor	6.45	3.29	4.16	1.96	2.36	1.00	0.17	0.06	0.18	0.06	0.22	0.07
Progesterone receptor	5.40	3.79	3.81	2.41	2.46	1.33	0.16	0.11	0.19	0.12	0.25	0.13
β_2-adrenergic receptor	9.33	4.37	6.20	2.77	3.65	1.45	0.29	0.14	0.32	0.15	0.38	0.16
c-Jun N-terminal kinase I	7.36	4.09	4.74	2.54	2.67	1.27	0.23	0.13	0.25	0.13	0.28	0.13

Cyclooxygenase-2	5.43	5.26	3.67	3.29	2.25	1.75	0.17	0.15	0.19	0.16	0.24	0.18
Glycine transporter 1	5.75	2.38	3.81	1.37	2.35	0.79	0.17	0.06	0.19	0.06	0.23	0.07
Dopamine receptor D3	8.99	6.00	6.44	4.17	4.03	2.38	0.28	0.18	0.34	0.21	0.42	0.23
C5a anaphylatoxin chemotactic receptor	10.14	5.14	6.36	3.16	3.47	1.50	0.32	0.17	0.33	0.17	0.37	0.16
C-C chemokine receptor type 4	7.78	5.71	4.98	3.50	2.78	1.77	0.18	0.09	0.20	0.09	0.23	0.10
Interleukin-8 receptor B	7.58	2.93	4.98	1.77	2.85	0.90	0.23	0.09	0.25	0.09	0.29	0.09
Muscarinic acetylcholine receptor M3	6.34	4.72	4.39	2.97	2.68	1.59	0.21	0.16	0.25	0.16	0.29	0.17
Phosphodiesterase 4A	4.19	1.76	2.98	1.19	2.02	0.76	0.14	0.07	0.17	0.08	0.23	0.09
Liver glycogen phosphorylase	10.02	3.32	6.43	2.05	3.66	0.95	0.28	0.11	0.31	0.11	0.36	0.10
Tyrosine-protein kinase SRC	6.08	3.66	4.24	2.38	2.69	1.36	0.19	0.12	0.22	0.13	0.28	0.15
Renin	5.30	2.08	3.68	1.56	2.35	1.08	0.19	0.09	0.21	0.11	0.26	0.14
Phosphodiesterase 4D	3.45	2.75	2.66	1.96	1.96	1.25	0.11	0.06	0.14	0.07	0.21	0.10
Cathepsin S	3.48	1.90	2.50	1.34	1.66	0.83	0.12	0.06	0.14	0.08	0.19	0.10
Carbonic anhydrase XII	3.10	2.46	2.43	1.77	1.72	1.06	0.11	0.08	0.14	0.09	0.20	0.11
Cytochrome P450 2C9	1.46	1.29	1.11	0.83	0.83	0.50	0.04	0.03	0.05	0.03	0.08	0.04
Urotensin II receptor	5.92	2.93	3.94	2.04	2.30	1.19	0.18	0.08	0.20	0.10	0.24	0.11
Metabotropic glutamate receptor 1	9.51	4.59	6.09	2.85	3.59	1.39	0.28	0.10	0.31	0.11	0.38	0.10
Nicotinic acid receptor 1	12.21	4.71	7.57	2.86	3.99	1.39	0.38	0.16	0.38	0.16	0.41	0.15
Cathepsin L	3.69	2.50	2.57	1.67	1.64	1.00	0.12	0.08	0.14	0.09	0.19	0.11
C-C chemokine receptor type 2	6.90	4.31	4.77	2.80	3.00	1.61	0.20	0.11	0.24	0.12	0.30	0.14
Cathepsin B	4.44	2.38	3.05	1.59	1.86	0.91	0.15	0.08	0.17	0.09	0.21	0.11
Estradiol 17-β-dehydrogenase 3	9.21	3.65	5.90	2.15	3.26	1.17	0.27	0.11	0.30	0.11	0.35	0.11
Sodium channel protein type IX α subunit	13.71	9.18	8.57	5.58	4.54	2.81	0.38	0.26	0.40	0.26	0.44	0.26
Voltage-gated potassium channel subunit Kv1.5	3.60	1.85	2.55	1.29	1.70	0.82	0.11	0.06	0.14	0.07	0.18	0.09
Sphingosine 1-phosphate receptor Edg-1	9.89	6.14	6.87	4.09	4.16	2.28	0.32	0.19	0.37	0.21	0.44	0.23
Neuropeptide Y receptor type 5	7.59	4.20	5.32	2.94	3.26	1.75	0.25	0.16	0.29	0.18	0.35	0.19

Ghrelin receptor	4.07	3.36	2.70	2.10	1.64	1.14	0.13	0.09	0.14	0.10	0.18	0.11
Leukotriene A4 hydrolase	13.75	5.59	9.43	3.45	5.63	1.92	0.46	0.20	0.51	0.21	0.59	0.22
Serine/threonine-protein kinase Aurora-A	4.60	4.07	3.13	2.53	1.96	1.38	0.13	0.11	0.15	0.11	0.20	0.13
Neuropeptide Y receptor type 1	2.99	2.52	2.05	1.66	1.42	0.99	0.08	0.06	0.10	0.07	0.15	0.09
Orexin receptor 2	8.09	4.82	5.42	2.99	3.23	1.51	0.25	0.14	0.28	0.14	0.34	0.14
P2X purinoceptor 7	7.27	2.47	5.12	1.62	3.14	0.89	0.16	0.05	0.19	0.05	0.26	0.06
β-secretase 1	5.58	3.92	3.58	2.34	2.03	1.15	0.14	0.08	0.15	0.08	0.18	0.08
G protein-coupled receptor 44	8.70	4.41	6.11	2.92	3.78	1.60	0.29	0.16	0.34	0.17	0.41	0.18
Serine/threonine-protein kinase B-raf	4.54	2.12	3.18	1.40	2.04	0.85	0.13	0.06	0.16	0.07	0.21	0.09
Average	7.20	4.02	4.82	2.56	2.87	1.38	0.21	0.11	0.24	0.12	0.29	0.13

Table S6. Mean SRR1% Values and Their Standard Deviations Obtained with Different Methods for the Individual Targets.

Target name	2D single query[1]	Stdev	3D single query[1]	Stdev	2D five queries[1]	Stdev	3D five queries[1]	Stdev	Balanced, parallel selection[1]	Stdev	Integrated approach[2]	Stdev
Somatostatin receptor 5	0.32	0.16	0.30	0.20	0.65	0.11	0.64	0.09	0.49	0.18	0.76	0.06
Macrophage colony stimulating factor receptor	0.14	0.09	0.13	0.09	0.40	0.10	0.41	0.11	0.21	0.08	0.54	0.08
Tyrosine-protein kinase ABL	0.20	0.11	0.20	0.12	0.43	0.07	0.38	0.06	0.29	0.11	0.48	0.05
Vasopressin V1a receptor	0.11	0.05	0.11	0.07	0.29	0.05	0.25	0.06	0.17	0.05	0.39	0.04
Melatonin receptor 1B	0.41	0.16	0.37	0.13	0.68	0.06	0.59	0.04	0.54	0.15	0.81	0.05
Thymidylate synthase	0.18	0.10	0.16	0.09	0.49	0.07	0.40	0.08	0.28	0.10	0.62	0.09
Insulin-like growth factor I receptor	0.28	0.09	0.24	0.08	0.47	0.05	0.40	0.05	0.36	0.07	0.50	0.04
Tyrosine-protein kinase receptor FLT3	0.08	0.05	0.06	0.04	0.21	0.04	0.13	0.03	0.11	0.06	0.25	0.04
Serotonin 1d (5-HT1d) receptor	0.22	0.15	0.17	0.11	0.48	0.06	0.35	0.09	0.31	0.13	0.54	0.06
Inhibitor of nuclear factor κ B kinase β subunit	0.31	0.17	0.30	0.19	0.49	0.04	0.51	0.05	0.41	0.09	0.55	0.06
Glucocorticoid receptor	0.16	0.11	0.10	0.07	0.33	0.08	0.26	0.07	0.21	0.10	0.42	0.06

Oxytocin receptor	0.13	0.05	0.14	0.05	0.39	0.07	0.37	0.07	0.22	0.07	0.50	0.08
Progesterone receptor	0.13	0.09	0.12	0.09	0.29	0.11	0.24	0.10	0.18	0.10	0.36	0.11
β_2-adrenergic receptor	0.42	0.19	0.23	0.13	0.62	0.09	0.35	0.10	0.45	0.15	0.60	0.06
c-Jun N-terminal kinase I	0.21	0.11	0.20	0.13	0.43	0.08	0.40	0.07	0.30	0.13	0.49	0.09
Cyclooxygenase-2	0.17	0.13	0.13	0.13	0.29	0.09	0.30	0.08	0.21	0.11	0.36	0.05
Glycine transporter 1	0.17	0.07	0.15	0.06	0.49	0.09	0.44	0.08	0.27	0.08	0.65	0.09
Dopamine receptor D3	0.16	0.07	0.17	0.11	0.31	0.04	0.25	0.07	0.22	0.07	0.33	0.03
C5a anaphylatoxin chemotactic receptor	0.38	0.15	0.29	0.17	0.80	0.14	0.60	0.11	0.55	0.22	0.88	0.06
C-C chemokine receptor type 4	0.20	0.10	0.16	0.08	0.49	0.11	0.46	0.09	0.29	0.11	0.66	0.09
Interleukin-8 receptor B	0.17	0.07	0.19	0.08	0.46	0.09	0.47	0.10	0.28	0.08	0.62	0.11
Muscarinic acetylcholine receptor M3	0.14	0.11	0.17	0.15	0.32	0.10	0.33	0.11	0.23	0.13	0.44	0.08
Phosphodiesterase 4A	0.11	0.06	0.11	0.06	0.34	0.09	0.29	0.08	0.18	0.08	0.46	0.11
Liver glycogen phosphorylase	0.30	0.11	0.26	0.11	0.68	0.13	0.59	0.14	0.42	0.15	0.77	0.05
Tyrosine-protein kinase SRC	0.18	0.11	0.15	0.10	0.37	0.08	0.29	0.07	0.25	0.12	0.44	0.05
Renin	0.25	0.09	0.15	0.07	0.51	0.11	0.33	0.05	0.30	0.08	0.57	0.07
Phosphodiesterase 4D	0.09	0.06	0.07	0.05	0.26	0.08	0.19	0.08	0.13	0.07	0.34	0.10
Cathepsin S	0.13	0.06	0.08	0.04	0.29	0.05	0.18	0.04	0.16	0.06	0.35	0.05
Carbonic anhydrase XII	0.17	0.10	0.07	0.05	0.23	0.06	0.11	0.05	0.15	0.07	0.21	0.05
Cytochrome P450 2C9	0.03	0.02	0.02	0.02	0.07	0.02	0.06	0.02	0.04	0.02	0.09	0.02
Urotensin II receptor	0.23	0.11	0.15	0.07	0.53	0.10	0.42	0.09	0.30	0.10	0.64	0.06
Metabotropic glutamate receptor 1	0.23	0.09	0.25	0.10	0.57	0.10	0.61	0.09	0.38	0.13	0.70	0.06
Nicotinic acid receptor 1	0.33	0.13	0.36	0.16	0.74	0.14	0.79	0.12	0.49	0.16	0.88	0.08
Cathepsin L	0.16	0.08	0.09	0.06	0.35	0.06	0.21	0.06	0.18	0.08	0.39	0.06
C-C chemokine receptor type 2	0.28	0.13	0.15	0.10	0.50	0.07	0.34	0.09	0.31	0.11	0.52	0.06
Cathepsin B	0.18	0.09	0.11	0.06	0.40	0.08	0.29	0.08	0.22	0.09	0.46	0.06
Estradiol 17-β-dehydrogenase 3	0.26	0.09	0.24	0.10	0.71	0.12	0.61	0.13	0.45	0.14	0.79	0.02

Sodium channel protein type IX α subunit	0.28	0.16	0.28	0.19	0.42	0.03	0.42	0.02	0.30	0.11	0.42	0.02
Voltage-gated potassium channel subunit Kv1.5	0.09	0.05	0.09	0.05	0.29	0.08	0.28	0.08	0.15	0.07	0.42	0.10
Sphingosine 1-phosphate receptor Edg-1	0.17	0.10	0.23	0.14	0.34	0.08	0.30	0.15	0.27	0.11	0.40	0.09
Neuropeptide Y receptor type 5	0.19	0.12	0.20	0.14	0.50	0.11	0.49	0.12	0.29	0.13	0.64	0.10
Ghrelin receptor	0.12	0.11	0.10	0.08	0.39	0.10	0.26	0.07	0.18	0.10	0.45	0.10
Leukotriene A4 hydrolase	0.32	0.16	0.35	0.19	0.70	0.11	0.62	0.19	0.48	0.17	0.82	0.12
Serine/threonine-protein kinase Aurora-A	0.13	0.11	0.10	0.10	0.29	0.09	0.21	0.06	0.16	0.11	0.32	0.05
Neuropeptide Y receptor type 1	0.07	0.06	0.06	0.05	0.21	0.08	0.11	0.04	0.11	0.07	0.27	0.06
Orexin receptor 2	0.16	0.09	0.20	0.13	0.42	0.09	0.45	0.11	0.27	0.12	0.57	0.08
P2X purinoceptor 7	0.17	0.06	0.11	0.04	0.34	0.07	0.28	0.04	0.21	0.06	0.37	0.06
β-secretase 1	0.15	0.07	0.12	0.07	0.25	0.04	0.23	0.03	0.17	0.05	0.25	0.02
G protein-coupled receptor 44	0.17	0.08	0.20	0.11	0.30	0.05	0.36	0.08	0.24	0.08	0.40	0.06
Serine/threonine-protein kinase B-raf	0.15	0.07	0.09	0.05	0.30	0.07	0.21	0.05	0.19	0.08	0.34	0.05
Average	0.20	0.10	0.17	0.10	0.42	0.08	0.36	0.08	0.27	0.10	0.50	0.07

[1] Reported values are averages obtained from 30 screening runs with different (sets of) query molecules.

[2] Using sets of five query molecules in combination with balanced, parallel selection.

Table S7. Median EFs, SRRs and Standard Deviations Obtained by the Different Approaches for the Individual Targets.

Target Name	2D								3D							
	Enrichment factor				Scaffold recovery rate				Enrichment factor				Scaffold recovery rate			
	1%	3%	5%	10%	1%	3%	5%	10%	1%	3%	5%	10%	1%	3%	5%	10%
Somatostatin receptor 5	35.69	13.52	8.66	5.01	0.28	0.32	0.36	0.45	28.32	13.31	8.89	5.58	0.24	0.34	0.39	0.49
Macrophage colony stimulating factor receptor	10.73	4.68	3.64	2.67	0.09	0.15	0.20	0.29	15.11	6.57	4.31	2.48	0.14	0.19	0.19	0.22
Tyrosine-protein kinase ABL	19.87	9.32	6.80	4.95	0.25	0.31	0.35	0.44	20.31	9.32	6.42	3.80	0.20	0.26	0.29	0.36
Vasopressin V1a receptor	10.00	4.24	2.91	2.12	0.10	0.13	0.15	0.23	9.23	4.56	3.15	1.97	0.09	0.14	0.15	0.20
Melatonin receptor 1B	38.62	17.59	11.77	6.81	0.41	0.60	0.66	0.77	42.83	18.18	11.88	6.50	0.40	0.50	0.54	0.59
Thymidylate synthase	12.73	5.96	4.61	3.06	0.15	0.23	0.29	0.34	9.70	4.85	3.58	2.45	0.15	0.24	0.27	0.31
Insulin-like growth factor I receptor	26.53	12.94	9.21	5.78	0.29	0.38	0.45	0.57	22.82	9.43	6.40	3.77	0.25	0.31	0.36	0.43
Tyrosine-protein kinase receptor FLT3	7.03	4.30	3.25	2.37	0.07	0.14	0.18	0.26	6.06	3.22	2.35	1.37	0.07	0.09	0.11	0.14
Serotonin 1d (5-HT1d) receptor	13.80	9.48	7.05	4.60	0.15	0.28	0.35	0.43	17.59	7.69	5.52	3.76	0.16	0.21	0.25	0.34
Inhibitor of nuclear factor κ B kinase β subunit	58.07	19.73	12.08	6.30	0.42	0.46	0.48	0.54	56.95	19.26	11.68	5.94	0.42	0.43	0.45	0.47
Glucocorticoid receptor	14.15	7.14	4.98	3.22	0.21	0.28	0.31	0.36	9.87	4.03	2.66	1.68	0.09	0.10	0.12	0.17
Oxytocin receptor	16.02	6.40	4.47	3.10	0.15	0.18	0.21	0.27	18.01	6.57	4.23	2.58	0.15	0.19	0.21	0.23
Progesterone receptor	12.23	4.91	3.34	2.13	0.12	0.17	0.21	0.28	7.98	5.47	4.06	2.36	0.09	0.16	0.18	0.23
β_2-adrenergic receptor	41.00	18.49	12.54	7.63	0.41	0.58	0.64	0.72	22.44	9.61	6.40	3.70	0.21	0.29	0.32	0.40
c-Jun N-terminal kinase I	21.33	9.63	6.52	4.08	0.21	0.31	0.35	0.44	22.80	9.10	5.70	3.13	0.23	0.28	0.31	0.34
Cyclooxygenase-2	16.34	8.42	6.31	4.23	0.18	0.25	0.32	0.45	6.00	2.62	1.84	1.30	0.05	0.08	0.10	0.17
Glycine transporter 1	17.33	7.04	4.55	2.99	0.18	0.21	0.24	0.32	14.08	5.16	3.57	2.50	0.15	0.19	0.22	0.24
Dopamine receptor D3	17.36	7.93	6.04	4.20	0.18	0.25	0.33	0.46	20.30	9.96	7.09	4.52	0.19	0.32	0.38	0.47

C5a anaphylatoxin chemotactic receptor	42.60	15.32	9.46	4.93	0.43	0.47	0.48	0.55	36.52	13.07	8.11	4.32	0.34	0.35	0.38	0.42
C-C chemokine receptor type 4	13.59	6.82	5.20	3.88	0.23	0.27	0.29	0.34	12.80	4.92	3.58	2.72	0.17	0.21	0.23	0.24
Interleukin-8 receptor B	19.55	7.82	5.20	3.18	0.19	0.23	0.25	0.31	20.20	8.23	5.44	3.07	0.19	0.23	0.26	0.29
Muscarinic acetylcholine receptor M3	9.55	4.76	3.76	2.85	0.09	0.15	0.18	0.30	11.06	5.78	4.35	2.68	0.12	0.19	0.25	0.30
Phosphodiesterase 4A	9.49	4.43	3.09	1.96	0.12	0.17	0.20	0.26	10.31	4.25	2.90	1.98	0.12	0.14	0.18	0.25
Liver glycogen phosphorylase	33.75	13.19	8.83	5.34	0.32	0.34	0.39	0.49	31.10	11.31	7.42	4.10	0.30	0.34	0.36	0.40
Tyrosine-protein kinase SRC	18.46	8.67	6.09	4.20	0.17	0.27	0.33	0.44	16.35	7.36	4.93	2.91	0.18	0.23	0.26	0.30
Renin	21.17	9.28	6.58	4.59	0.26	0.35	0.41	0.53	11.19	5.48	3.80	2.29	0.16	0.20	0.21	0.26
Phosphodiesterase 4D	6.95	4.53	3.64	2.32	0.08	0.14	0.17	0.25	5.06	2.60	2.04	1.53	0.06	0.10	0.14	0.19
Cathepsin S	11.03	5.53	4.38	2.95	0.13	0.19	0.26	0.33	6.91	2.82	2.05	1.52	0.07	0.10	0.12	0.17
Carbonic anhydrase XII	14.37	9.53	7.49	5.13	0.17	0.32	0.40	0.56	3.06	1.85	1.54	1.14	0.04	0.07	0.11	0.16
Cytochrome P450 2C9	2.95	2.07	1.73	1.25	0.03	0.05	0.07	0.11	1.50	0.94	0.79	0.72	0.01	0.03	0.04	0.07
Urotensin II receptor	19.16	10.15	7.26	4.74	0.20	0.33	0.37	0.48	14.92	5.92	3.87	2.28	0.14	0.19	0.22	0.26
Metabotropic glutamate receptor 1	28.29	9.51	5.72	2.96	0.28	0.28	0.29	0.31	28.38	9.51	5.95	3.39	0.28	0.28	0.30	0.39
Nicotinic acid receptor 1	34.19	13.39	8.34	4.44	0.37	0.40	0.41	0.47	38.63	13.39	8.15	4.21	0.42	0.42	0.43	0.44
Cathepsin L	12.78	5.95	4.31	2.83	0.17	0.23	0.29	0.37	8.87	3.63	2.49	1.55	0.09	0.11	0.14	0.18
C-C chemokine receptor type 2	36.57	14.79	10.27	6.31	0.31	0.38	0.49	0.60	13.28	5.76	3.92	2.85	0.11	0.17	0.21	0.28
Cathepsin B	14.49	7.91	5.48	3.77	0.17	0.28	0.32	0.42	11.52	5.24	3.32	1.98	0.13	0.17	0.18	0.21
Estradiol 17-β-dehydrogenase 3	33.09	11.03	6.62	3.31	0.30	0.31	0.35	0.41	25.57	9.65	6.24	3.61	0.27	0.31	0.34	0.40
Sodium channel protein type IX α subunit	39.60	18.04	11.88	6.64	0.37	0.51	0.57	0.64	43.19	19.64	12.27	6.45	0.40	0.54	0.57	0.61
Voltage-gated potassium channel subunit Kv1.5	9.05	3.67	2.53	1.83	0.09	0.10	0.15	0.21	8.01	3.67	2.76	1.97	0.09	0.12	0.15	0.22
Sphingosine 1-phosphate receptor Edg-1	18.48	9.88	6.90	4.38	0.18	0.30	0.35	0.42	21.30	10.19	7.47	4.57	0.24	0.35	0.42	0.54
Neuropeptide Y receptor type 5	14.22	5.84	4.19	2.79	0.15	0.20	0.22	0.32	14.05	5.84	4.58	3.36	0.12	0.18	0.23	0.32
Ghrelin receptor	6.35	3.02	2.57	1.90	0.08	0.12	0.14	0.19	6.07	2.65	2.03	1.16	0.08	0.11	0.11	0.14
Leukotriene A4 hydrolase	27.19	12.83	10.57	6.74	0.30	0.44	0.55	0.71	31.76	15.80	10.78	6.13	0.42	0.54	0.58	0.66
Serine/threonine-protein kinase Aurora-A	6.34	4.44	3.84	3.00	0.09	0.16	0.22	0.33	4.33	2.41	1.88	1.60	0.05	0.09	0.13	0.19

Neuropeptide Y receptor type 1	6.46	2.58	2.10	1.37	0.04	0.06	0.09	0.14	5.91	2.65	1.96	1.29	0.04	0.07	0.09	0.13
Orexin receptor 2	14.83	6.09	4.51	3.61	0.16	0.26	0.28	0.38	24.14	11.22	7.13	3.95	0.25	0.33	0.36	0.40
P2X purinoceptor 7	18.35	9.49	6.53	3.85	0.16	0.24	0.28	0.39	16.36	7.89	5.64	3.39	0.10	0.15	0.19	0.25
β-secretase 1	17.03	9.94	6.79	3.96	0.18	0.23	0.27	0.33	9.70	4.01	2.64	1.66	0.11	0.14	0.16	0.21
G protein-coupled receptor 44	14.37	8.00	6.44	4.63	0.16	0.28	0.36	0.50	16.68	8.39	6.06	3.89	0.18	0.28	0.33	0.42
Serine/threonine-protein kinase B-raf	15.17	7.37	5.52	3.78	0.16	0.24	0.29	0.39	9.25	4.18	3.10	2.07	0.09	0.13	0.16	0.21
Average	19.57	8.75	6.13	3.89	0.20	0.27	0.32	0.40	17.37	7.38	4.98	2.99	0.17	0.22	0.25	0.31

Table S8. Overall AUC values, EFs, SRRs and Standard Deviations Obtained with Different Methods Using Query Sets of Different Size.

Methods	2D										3D									
Query set size	AUC	Stdev	EF1%	Stdev	EF10%	Stdev	SRR1%	Stdev	SRR10%	Stdev	AUC	Stdev	EF1%	Stdev	EF10%	Stdev	SRR1%	Stdev	SRR10%	Stdev
1	0.68	0.10	19.96	10.87	3.69	1.43	0.20	0.10	0.38	0.14	0.54	0.13	17.52	10.53	2.87	1.38	0.17	0.10	0.29	0.13
2	0.73	0.08	28.83	10.69	4.68	1.24	0.28	0.10	0.47	0.12	0.60	0.11	25.66	10.38	3.74	1.27	0.24	0.09	0.37	0.12
3	0.77	0.07	35.70	9.84	5.36	1.08	0.34	0.09	0.53	0.10	0.64	0.09	31.48	9.77	4.37	1.15	0.29	0.09	0.42	0.11
4	0.80	0.06	40.81	9.10	5.87	0.96	0.39	0.09	0.58	0.09	0.67	0.08	36.06	9.04	4.85	1.02	0.33	0.08	0.46	0.10
5	0.82	0.05	44.59	8.48	6.23	0.88	0.42	0.08	0.61	0.08	0.69	0.07	39.48	8.39	5.20	0.93	0.36	0.08	0.50	0.09

Table S9. Mean AUC Values and Standard Deviations Obtained by the 2D Fingerprint-based Approach for the Individual Targets Using Query Sets of Different Size.

Target Name	One query		Two queries		Three queries		Four queries		Five queries	
	AUC[1]	Stdev	AUC[1]	Stdev	AUC[1]	Stdev	AUC[1]	Stdev	AUC[1]	Stdev
Somatostatin receptor 5	0.77	0.11	0.85	0.07	0.89	0.07	0.92	0.05	0.92	0.05
Macrophage colony stimulating factor receptor	0.65	0.07	0.70	0.06	0.73	0.07	0.77	0.05	0.80	0.05
Tyrosine-protein kinase ABL	0.74	0.11	0.79	0.06	0.82	0.04	0.82	0.04	0.83	0.03
Vasopressin V1a receptor	0.62	0.07	0.66	0.06	0.70	0.07	0.73	0.07	0.75	0.06
Melatonin receptor 1B	0.87	0.08	0.93	0.05	0.95	0.02	0.96	0.01	0.97	0.01
Thymidylate synthase	0.65	0.15	0.71	0.13	0.78	0.10	0.82	0.06	0.85	0.05
Insulin-like growth factor I receptor	0.83	0.07	0.87	0.04	0.88	0.03	0.89	0.02	0.90	0.02
Tyrosine-protein kinase receptor FLT3	0.58	0.10	0.59	0.09	0.62	0.07	0.65	0.07	0.68	0.07
Serotonin 1d (5-HT1d) receptor	0.74	0.11	0.80	0.09	0.85	0.05	0.87	0.02	0.88	0.02
Inhibitor of nuclear factor κ B kinase β subunit	0.74	0.13	0.81	0.06	0.83	0.03	0.83	0.03	0.84	0.03
Glucocorticoid receptor	0.57	0.14	0.62	0.13	0.67	0.09	0.70	0.08	0.72	0.06
Oxytocin receptor	0.58	0.09	0.68	0.10	0.74	0.10	0.77	0.09	0.80	0.09
Progesterone receptor	0.57	0.12	0.61	0.12	0.66	0.11	0.69	0.10	0.71	0.09
β_2-adrenergic receptor	0.88	0.09	0.91	0.03	0.93	0.02	0.93	0.02	0.94	0.01
c-Jun N-terminal kinase I	0.71	0.07	0.77	0.05	0.80	0.05	0.83	0.05	0.85	0.06
Cyclooxygenase-2	0.63	0.16	0.69	0.11	0.71	0.09	0.73	0.05	0.75	0.04
Glycine transporter 1	0.64	0.07	0.71	0.07	0.78	0.05	0.82	0.06	0.83	0.06
Dopamine receptor D3	0.74	0.11	0.78	0.07	0.82	0.04	0.83	0.04	0.84	0.04

C5a anaphylatoxin chemotactic receptor	0.71	0.13	0.83	0.12	0.89	0.09	0.93	0.06	0.95	0.03
C-C chemokine receptor type 4	0.66	0.16	0.74	0.12	0.79	0.08	0.81	0.06	0.84	0.07
Interleukin-8 receptor B	0.62	0.11	0.71	0.09	0.77	0.07	0.81	0.06	0.83	0.06
Muscarinic acetylcholine receptor M3	0.65	0.10	0.69	0.09	0.71	0.07	0.74	0.05	0.76	0.04
Phosphodiesterase 4A	0.52	0.10	0.57	0.10	0.63	0.08	0.67	0.06	0.70	0.06
Liver glycogen phosphorylase	0.81	0.06	0.87	0.05	0.91	0.05	0.92	0.04	0.94	0.02
Tyrosine-protein kinase SRC	0.68	0.11	0.72	0.09	0.75	0.06	0.77	0.05	0.79	0.04
Renin	0.76	0.08	0.82	0.06	0.85	0.04	0.87	0.04	0.88	0.04
Phosphodiesterase 4D	0.57	0.09	0.60	0.08	0.64	0.08	0.66	0.07	0.71	0.07
Cathepsin S	0.68	0.07	0.73	0.07	0.75	0.07	0.78	0.05	0.81	0.05
Carbonic anhydrase XII	0.71	0.14	0.78	0.07	0.80	0.06	0.81	0.04	0.82	0.03
Cytochrome P450 2C9	0.48	0.08	0.49	0.07	0.50	0.06	0.51	0.05	0.52	0.05
Urotensin II receptor	0.75	0.07	0.79	0.04	0.81	0.03	0.84	0.03	0.85	0.03
Metabotropic glutamate receptor 1	0.62	0.13	0.69	0.12	0.74	0.13	0.81	0.11	0.82	0.09
Nicotinic acid receptor 1	0.60	0.09	0.74	0.15	0.79	0.15	0.85	0.13	0.90	0.11
Cathepsin L	0.64	0.09	0.71	0.06	0.75	0.04	0.78	0.03	0.80	0.04
C-C chemokine receptor type 2	0.79	0.13	0.87	0.05	0.89	0.03	0.90	0.03	0.91	0.02
Cathepsin B	0.67	0.12	0.75	0.07	0.77	0.06	0.78	0.06	0.81	0.04
Estradiol 17-β-dehydrogenase 3	0.63	0.12	0.70	0.10	0.77	0.12	0.84	0.11	0.89	0.07
Sodium channel protein type IX α subunit	0.75	0.19	0.84	0.05	0.87	0.03	0.87	0.02	0.88	0.02
Voltage-gated potassium channel subunit Kv1.5	0.52	0.09	0.56	0.07	0.60	0.09	0.65	0.09	0.67	0.08
Sphingosine 1-phosphate receptor Edg-1	0.67	0.11	0.72	0.10	0.77	0.06	0.79	0.04	0.82	0.04
Neuropeptide Y receptor type 5	0.63	0.12	0.71	0.10	0.76	0.10	0.81	0.11	0.83	0.10
Ghrelin receptor	0.60	0.09	0.65	0.07	0.68	0.07	0.71	0.09	0.75	0.07
Leukotriene A4 hydrolase	0.83	0.13	0.89	0.09	0.92	0.06	0.94	0.04	0.95	0.03
Serine/threonine-protein kinase Aurora-A	0.68	0.09	0.74	0.06	0.76	0.05	0.78	0.04	0.80	0.04

Neuropeptide Y receptor type 1	0.47	0.08	0.52	0.07	0.53	0.08	0.55	0.08	0.56	0.07
Orexin receptor 2	0.67	0.09	0.71	0.08	0.74	0.05	0.77	0.04	0.80	0.05
P2X purinoceptor 7	0.73	0.06	0.78	0.04	0.80	0.03	0.83	0.03	0.84	0.03
β-secretase 1	0.64	0.12	0.70	0.09	0.74	0.07	0.77	0.06	0.79	0.07
G protein-coupled receptor 44	0.79	0.08	0.84	0.05	0.86	0.04	0.88	0.03	0.89	0.03
Serine/threonine-protein kinase B-raf	0.72	0.09	0.78	0.08	0.81	0.06	0.84	0.04	0.86	0.03
Average	0.68	0.10	0.73	0.08	0.77	0.07	0.80	0.06	0.82	0.05

[1] Reported values are averages obtained from 30 screening runs with different (sets of) query molecules.

Table S10. Mean AUC Values and Standard Deviations Obtained by the 3D Shape-based Approach for the Individual Targets Using Query Sets of Different Size.

Target Name	One query		Two queries		Three queries		Four queries		Five queries	
	AUC[1]	Stdev	AUC[1]	Stdev	AUC[1]	Stdev	AUC[1]	Stdev	AUC[1]	Stdev
Somatostatin receptor 5	0.71	0.18	0.79	0.11	0.82	0.10	0.86	0.05	0.88	0.03
Macrophage colony stimulating factor receptor	0.48	0.15	0.52	0.14	0.57	0.10	0.62	0.09	0.66	0.10
Tyrosine-protein kinase ABL	0.57	0.12	0.66	0.07	0.71	0.07	0.73	0.06	0.74	0.06
Vasopressin V1a receptor	0.51	0.13	0.53	0.08	0.56	0.08	0.57	0.08	0.60	0.06
Melatonin receptor 1B	0.76	0.09	0.83	0.04	0.86	0.04	0.88	0.03	0.88	0.03
Thymidylate synthase	0.58	0.13	0.66	0.10	0.71	0.07	0.74	0.08	0.78	0.07
Insulin-like growth factor I receptor	0.65	0.12	0.68	0.09	0.69	0.09	0.71	0.08	0.72	0.08
Tyrosine-protein kinase receptor FLT3	0.43	0.14	0.42	0.11	0.46	0.08	0.47	0.08	0.48	0.07
Serotonin 1d (5-HT1d) receptor	0.61	0.18	0.68	0.13	0.69	0.11	0.71	0.12	0.76	0.07
Inhibitor of nuclear factor κ B kinase β subunit	0.59	0.21	0.72	0.15	0.76	0.10	0.80	0.03	0.81	0.03
Glucocorticoid receptor	0.44	0.12	0.47	0.11	0.51	0.10	0.53	0.07	0.57	0.07

Oxytocin receptor	0.50	0.09	0.57	0.09	0.63	0.10	0.68	0.11	0.71	0.10
Progesterone receptor	0.54	0.12	0.60	0.11	0.65	0.08	0.69	0.09	0.69	0.08
β_2-adrenergic receptor	0.59	0.13	0.61	0.12	0.64	0.10	0.67	0.08	0.70	0.07
c-Jun N-terminal kinase I	0.50	0.11	0.58	0.12	0.67	0.09	0.70	0.08	0.72	0.06
Cyclooxygenase-2	0.50	0.15	0.61	0.10	0.63	0.09	0.65	0.09	0.68	0.08
Glycine transporter 1	0.52	0.09	0.61	0.11	0.68	0.09	0.72	0.07	0.75	0.06
Dopamine receptor D3	0.64	0.22	0.71	0.15	0.73	0.12	0.73	0.10	0.75	0.09
C5a anaphylatoxin chemotactic receptor	0.51	0.15	0.52	0.13	0.58	0.11	0.63	0.11	0.66	0.09
C-C chemokine receptor type 4	0.51	0.12	0.62	0.10	0.67	0.09	0.71	0.09	0.76	0.07
Interleukin-8 receptor B	0.54	0.08	0.63	0.09	0.70	0.09	0.75	0.07	0.77	0.06
Muscarinic acetylcholine receptor M3	0.52	0.14	0.52	0.12	0.56	0.12	0.60	0.09	0.62	0.07
Phosphodiesterase 4A	0.51	0.08	0.57	0.05	0.61	0.07	0.63	0.05	0.65	0.06
Liver glycogen phosphorylase	0.62	0.11	0.70	0.12	0.77	0.12	0.82	0.10	0.84	0.10
Tyrosine-protein kinase SRC	0.55	0.14	0.58	0.14	0.62	0.10	0.63	0.09	0.64	0.06
Renin	0.51	0.16	0.53	0.09	0.57	0.08	0.61	0.08	0.63	0.06
Phosphodiesterase 4D	0.51	0.12	0.55	0.10	0.58	0.09	0.59	0.09	0.61	0.10
Cathepsin S	0.43	0.13	0.43	0.10	0.46	0.11	0.49	0.09	0.51	0.09
Carbonic anhydrase XII	0.44	0.10	0.47	0.09	0.51	0.10	0.53	0.08	0.55	0.07
Cytochrome P450 2C9	0.36	0.06	0.36	0.06	0.37	0.06	0.38	0.05	0.39	0.04
Urotensin II receptor	0.45	0.12	0.51	0.12	0.59	0.09	0.65	0.09	0.69	0.09
Metabotropic glutamate receptor 1	0.69	0.14	0.76	0.10	0.82	0.11	0.87	0.09	0.92	0.03
Nicotinic acid receptor 1	0.61	0.06	0.73	0.13	0.83	0.11	0.89	0.09	0.92	0.09
Cathepsin L	0.41	0.14	0.44	0.14	0.47	0.11	0.49	0.10	0.50	0.10
C-C chemokine receptor type 2	0.58	0.19	0.65	0.15	0.71	0.09	0.74	0.08	0.75	0.07
Cathepsin B	0.42	0.12	0.44	0.10	0.49	0.09	0.53	0.09	0.55	0.08
Estradiol 17-β-dehydrogenase 3	0.59	0.09	0.71	0.11	0.80	0.11	0.85	0.10	0.87	0.08

Sodium channel protein type IX α subunit	0.64	0.20	0.71	0.17	0.78	0.08	0.79	0.09	0.81	0.04
Voltage-gated potassium channel subunit Kv1.5	0.45	0.12	0.51	0.09	0.52	0.08	0.56	0.08	0.58	0.08
Sphingosine 1-phosphate receptor Edg-1	0.67	0.22	0.70	0.17	0.72	0.15	0.75	0.12	0.76	0.10
Neuropeptide Y receptor type 5	0.56	0.20	0.64	0.16	0.73	0.12	0.78	0.09	0.81	0.08
Ghrelin receptor	0.42	0.14	0.46	0.15	0.48	0.15	0.50	0.14	0.51	0.15
Leukotriene A4 hydrolase	0.78	0.15	0.83	0.09	0.85	0.08	0.88	0.05	0.90	0.04
Serine/threonine-protein kinase Aurora-A	0.47	0.15	0.50	0.12	0.51	0.10	0.55	0.07	0.56	0.08
Neuropeptide Y receptor type 1	0.43	0.16	0.41	0.15	0.41	0.12	0.43	0.10	0.45	0.08
Orexin receptor 2	0.64	0.10	0.71	0.05	0.74	0.03	0.75	0.03	0.76	0.03
P2X purinoceptor 7	0.62	0.05	0.67	0.05	0.72	0.04	0.75	0.03	0.77	0.03
β-secretase 1	0.42	0.07	0.48	0.06	0.51	0.06	0.54	0.05	0.56	0.05
G protein-coupled receptor 44	0.64	0.13	0.71	0.09	0.74	0.08	0.79	0.06	0.81	0.06
Serine/threonine-protein kinase B-raf	0.49	0.11	0.53	0.10	0.56	0.09	0.60	0.08	0.63	0.08
Average	0.54	0.13	0.60	0.11	0.64	0.09	0.67	0.08	0.69	0.07

[1] Reported values are averages obtained from 30 screening runs with different (sets of) query molecules.

Table S11. Mean EF1% values and Standard Deviations Obtained by the 2D Fingerprint-based Approach for the Individual Targets Using Query Sets of Different Size.

Target Name	One query		Two queries		Three queries		Four queries		Five queries	
	EF1%[1]	Stdev	EF1%[1]	Stdev	EF1%[1]	Stdev	EF1%[1]	Stdev	EF1%[1]	Stdev
Somatostatin receptor 5	36.86	21.21	50.04	19.33	63.90	18.84	71.39	14.77	74.01	13.49
Macrophage colony stimulating factor receptor	14.49	8.80	25.86	9.66	32.48	10.79	39.32	12.50	43.57	11.60
Tyrosine-protein kinase ABL	23.34	16.02	32.57	16.19	42.93	14.50	44.84	14.04	51.57	7.91
Vasopressin V1a receptor	10.57	4.66	16.01	4.60	20.08	4.72	25.02	5.89	29.50	6.00

Melatonin receptor 1B	36.60	16.42	47.23	11.37	52.60	8.61	58.33	6.41	62.94	5.22
Thymidylate synthase	20.57	16.38	31.55	17.18	41.15	14.51	49.28	11.00	53.93	9.51
Insulin-like growth factor I receptor	25.13	8.93	31.41	10.26	40.12	9.09	44.12	6.77	47.11	6.31
Tyrosine-protein kinase receptor FLT3	7.74	4.63	10.62	4.66	14.93	5.65	19.05	6.78	23.09	7.09
Serotonin 1d (5-HT1d) receptor	25.34	19.85	36.18	19.01	48.62	9.86	53.08	4.61	55.66	4.87
Inhibitor of nuclear factor κ B kinase β subunit	40.57	24.80	57.75	10.92	60.42	2.13	61.40	2.69	62.34	3.06
Glucocorticoid receptor	14.86	9.09	21.94	9.60	26.24	7.77	29.90	7.45	33.79	8.06
Oxytocin receptor	17.42	10.18	30.23	12.64	41.88	12.84	46.79	11.36	52.69	11.95
Progesterone receptor	11.83	7.16	17.48	9.62	24.23	10.41	28.13	10.17	30.71	10.29
β_2-adrenergic receptor	43.84	18.69	53.23	15.41	59.52	12.35	63.40	12.25	67.28	9.91
c-Jun N-terminal kinase I	19.54	9.80	29.87	8.49	36.23	8.16	41.98	9.23	44.68	9.33
Cyclooxygenase-2	14.98	11.73	17.97	9.53	21.05	9.79	22.17	7.99	25.52	7.37
Glycine transporter 1	16.51	7.15	28.45	8.95	37.38	8.79	44.10	10.03	48.28	10.25
Dopamine receptor D3	15.46	7.33	22.03	6.43	26.36	6.38	29.31	5.05	31.03	4.13
C5a anaphylatoxin chemotactic receptor	38.73	14.09	53.61	19.92	65.45	18.91	75.92	16.23	82.24	13.28
C-C chemokine receptor type 4	27.13	19.99	38.51	18.68	50.88	14.51	56.56	11.60	60.47	12.18
Interleukin-8 receptor B	17.20	7.20	27.65	8.54	36.57	8.86	40.54	9.30	44.48	8.89
Muscarinic acetylcholine receptor M3	12.38	9.28	18.49	8.53	23.28	8.91	27.50	8.54	30.79	8.82
Phosphodiesterase 4A	10.69	5.68	17.85	7.66	24.98	6.91	30.13	7.01	34.27	8.13
Liver glycogen phosphorylase	31.39	9.85	48.42	11.62	59.94	13.83	65.82	14.58	74.29	11.90
Tyrosine-protein kinase SRC	16.18	9.20	24.84	9.01	28.14	8.43	32.58	8.63	36.03	8.07
Renin	20.32	6.55	29.30	7.11	34.77	6.80	42.60	8.78	45.67	9.51
Phosphodiesterase 4D	9.53	7.39	13.39	7.74	17.76	9.10	21.72	8.27	27.28	10.25
Cathepsin S	12.29	5.29	17.68	5.84	20.97	5.80	24.94	5.33	29.49	5.85
Carbonic anhydrase XII	14.17	8.90	17.05	7.13	18.02	6.57	18.93	5.54	19.92	4.82
Cytochrome P450 2C9	3.86	3.17	5.79	3.70	7.42	4.09	8.92	4.41	9.89	4.42

Urotensin II receptor	22.19	11.59	32.76	11.76	38.65	12.67	47.57	11.90	53.42	10.44
Metabotropic glutamate receptor 1	25.07	12.69	38.29	15.14	49.43	16.19	59.35	14.58	62.08	12.78
Nicotinic acid receptor 1	31.13	10.99	47.34	14.49	56.59	13.14	63.60	12.86	70.37	13.51
Cathepsin L	13.62	8.09	19.12	8.14	25.46	7.08	30.77	6.84	33.46	7.03
C-C chemokine receptor type 2	28.75	15.67	42.13	14.41	47.37	12.19	51.32	7.77	54.25	6.42
Cathepsin B	16.62	8.24	24.11	6.65	30.00	7.69	33.80	7.46	37.48	7.27
Estradiol 17-β-dehydrogenase 3	28.53	11.70	43.42	13.70	58.73	17.81	72.31	17.35	79.70	14.30
Sodium channel protein type IX α subunit	29.39	17.82	37.93	11.21	44.39	5.79	47.49	3.14	47.92	2.93
Voltage-gated potassium channel subunit Kv1.5	8.85	4.64	14.14	5.93	20.18	6.62	26.08	6.72	30.04	6.95
Sphingosine 1-phosphate receptor Edg-1	17.17	11.37	20.63	9.54	25.84	8.70	31.26	8.70	34.97	8.28
Neuropeptide Y receptor type 5	17.64	10.34	28.42	12.51	36.96	12.50	43.43	10.72	46.98	11.15
Ghrelin receptor	12.22	11.85	18.63	12.74	26.58	12.86	33.72	13.72	40.20	10.94
Leukotriene A4 hydrolase	30.66	11.99	42.47	12.42	53.73	11.83	60.68	11.65	66.88	10.17
Serine/threonine-protein kinase Aurora-A	14.06	12.73	25.94	11.02	28.41	10.28	32.11	9.93	34.13	9.81
Neuropeptide Y receptor type 1	8.54	7.83	16.53	8.85	20.62	10.47	24.04	10.61	26.73	9.40
Orexin receptor 2	16.29	11.37	23.36	11.06	30.87	9.60	37.49	7.87	41.89	5.88
P2X purinoceptor 7	18.87	8.31	27.30	9.46	33.02	8.46	39.28	6.37	42.36	6.31
β-secretase 1	18.09	10.16	23.83	10.34	30.33	6.34	32.06	5.58	33.37	4.63
G protein-coupled receptor 44	15.57	8.34	21.22	7.79	25.56	6.11	28.07	5.87	29.93	5.38
Serine/threonine-protein kinase B-raf	15.46	8.24	20.70	8.09	23.77	7.62	28.24	7.98	30.86	7.93
Average	19.96	10.87	28.83	10.69	35.70	9.84	40.81	9.10	44.59	8.48

[1] Reported values are averages obtained from 30 screening runs with different (sets of) query molecules.

Table S12. Mean EF10% Values and Standard Deviations Obtained with the 2D Fingerprint-based Approach for the Individual Targets Using Query Sets of Different Size.

Target Name	One query		Two queries		Three queries		Four queries		Five queries	
	EF10%[1]	Stdev	EF10%[1]	Stdev	EF10%[1]	Stdev	EF10%[1]	Stdev	EF10%[1]	Stdev
Somatostatin receptor 5	5.28	2.21	6.67	1.53	7.70	1.41	8.20	0.96	8.32	0.99
Macrophage colony stimulating factor receptor	2.63	1.06	3.72	1.01	4.40	1.08	5.19	1.18	5.63	1.10
Tyrosine-protein kinase ABL	4.20	1.79	5.29	1.36	5.92	1.15	6.05	1.08	6.47	0.70
Vasopressin V1a receptor	2.33	0.89	2.89	0.89	3.44	1.00	4.02	1.03	4.46	0.87
Melatonin receptor 1B	6.57	1.97	8.03	1.09	8.51	0.58	8.89	0.42	9.08	0.29
Thymidylate synthase	3.45	1.92	4.52	1.84	5.58	1.58	6.36	1.02	6.78	0.90
Insulin-like growth factor I receptor	5.52	1.29	6.41	1.15	7.01	0.83	7.38	0.68	7.51	0.68
Tyrosine-protein kinase receptor FLT3	2.20	0.92	2.59	0.81	3.02	0.72	3.47	0.80	3.94	0.79
Serotonin 1d (5-HT1d) receptor	4.29	2.13	5.54	1.83	6.68	0.98	7.20	0.49	7.45	0.45
Inhibitor of nuclear factor κ B kinase β subunit	5.05	2.25	6.39	1.12	6.68	0.35	6.82	0.39	6.94	0.44
Glucocorticoid receptor	2.76	1.42	3.56	1.33	4.13	1.11	4.67	0.93	5.04	0.84
Oxytocin receptor	2.64	1.03	4.09	1.26	5.04	1.35	5.48	1.24	6.05	1.34
Progesterone receptor	2.36	1.17	3.01	1.36	3.74	1.30	4.19	1.23	4.59	1.21
β_2-adrenergic receptor	7.33	1.75	8.12	0.93	8.60	0.54	8.68	0.46	8.83	0.34
c-Jun N-terminal kinase I	3.67	1.27	4.80	0.93	5.32	1.07	5.92	1.17	6.30	1.16
Cyclooxygenase-2	3.30	2.02	3.96	1.44	4.46	1.29	4.77	0.92	5.14	0.78
Glycine transporter 1	2.84	0.82	4.05	1.13	5.18	0.98	5.77	1.10	6.14	1.17
Dopamine receptor D3	4.10	1.32	4.88	1.11	5.60	1.00	6.04	0.86	6.40	0.85
C5a anaphylatoxin chemotactic receptor	4.82	1.48	6.79	1.91	7.69	1.78	8.65	1.24	9.08	0.83
C-C chemokine receptor type 4	3.66	2.29	4.97	2.00	6.11	1.53	6.49	1.21	6.89	1.29
Interleukin-8 receptor B	3.12	1.26	4.33	1.17	5.34	1.04	5.71	0.91	6.06	0.95

Muscarinic acetylcholine receptor M3	2.79	1.36	3.59	1.16	4.11	1.13	4.49	1.03	4.89	1.01
Phosphodiesterase 4A	2.01	0.79	2.80	1.06	3.58	0.88	4.30	0.83	4.79	0.85
Liver glycogen phosphorylase	5.05	1.24	6.62	1.37	7.65	1.36	7.94	1.31	8.53	0.81
Tyrosine-protein kinase SRC	3.65	1.42	4.52	1.18	4.86	0.96	5.29	0.90	5.57	0.83
Renin	4.35	1.04	5.34	0.91	6.01	0.76	6.72	0.72	6.82	0.75
Phosphodiesterase 4D	2.26	0.99	2.77	1.18	3.30	1.18	3.70	1.04	4.42	1.19
Cathepsin S	3.22	0.92	3.88	1.00	4.33	0.90	4.76	0.68	5.26	0.78
Carbonic anhydrase XII	4.44	2.12	5.31	1.53	5.59	1.25	5.75	0.97	5.94	0.79
Cytochrome P450 2C9	1.23	0.63	1.43	0.59	1.57	0.64	1.81	0.57	1.92	0.55
Urotensin II receptor	4.19	1.21	5.01	0.86	5.48	0.77	6.07	0.68	6.44	0.64
Metabotropic glutamate receptor 1	3.14	1.28	4.35	1.57	5.45	1.76	6.48	1.51	6.73	1.30
Nicotinic acid receptor 1	3.93	1.25	5.82	1.78	6.73	1.68	7.50	1.48	8.16	1.34
Cathepsin L	3.25	1.38	4.24	1.08	5.10	0.85	5.62	0.69	5.96	0.76
C-C chemokine receptor type 2	5.63	2.21	6.85	1.35	7.17	0.93	7.46	0.80	7.77	0.67
Cathepsin B	3.74	1.41	4.72	1.04	5.18	1.04	5.57	1.13	6.09	0.99
Estradiol 17-β-dehydrogenase 3	3.49	1.32	4.71	1.32	6.13	1.76	7.44	1.71	8.17	1.38
Sodium channel protein type IX α subunit	5.22	2.51	6.59	1.05	7.06	0.42	7.25	0.33	7.33	0.31
Voltage-gated potassium channel subunit Kv1.5	1.77	0.78	2.45	0.75	3.04	0.93	3.72	0.94	4.08	0.89
Sphingosine 1-phosphate receptor Edg-1	3.59	1.86	4.47	1.48	5.17	1.15	5.69	0.99	6.14	0.92
Neuropeptide Y receptor type 5	3.19	1.36	4.37	1.18	5.40	1.39	6.26	1.39	6.67	1.32
Ghrelin receptor	2.23	1.40	2.93	1.46	3.73	1.49	4.40	1.60	5.06	1.28
Leukotriene A4 hydrolase	6.18	1.98	7.05	1.70	7.68	1.54	8.23	1.21	8.72	0.84
Serine/threonine-protein kinase Aurora-A	3.23	1.36	4.42	1.08	4.80	0.84	5.24	0.85	5.45	0.89
Neuropeptide Y receptor type 1	1.30	0.77	2.02	0.93	2.45	1.12	2.79	1.09	3.02	0.96
Orexin receptor 2	3.24	1.52	3.91	1.34	4.67	0.96	5.24	0.70	5.68	0.71
P2X purinoceptor 7	4.07	0.97	4.98	0.96	5.52	0.77	6.09	0.63	6.30	0.59

β-secretase 1	3.54	1.53	4.17	1.40	5.00	1.02	5.42	1.09	5.63	1.08
G protein-coupled receptor 44	4.63	1.52	5.51	1.26	6.14	1.00	6.46	0.79	6.73	0.74
Serine/threonine-protein kinase B-raf	3.63	1.30	4.64	1.30	5.13	1.09	5.78	0.79	6.07	0.72
Average	3.69	1.43	4.68	1.24	5.36	1.08	5.87	0.96	6.23	0.88

[1] Reported values are averages obtained from 30 screening runs with different (sets of) query molecules.

Table S13. Mean EF1% Values and Standard Deviations Obtained with the 3D Shape-based Approach for the Individual Targets Using Query Sets of Different Size.

Target Name	One query		Two queries		Three queries		Four queries		Five queries	
	EF1%[1]	Stdev	EF1%[1]	Stdev	EF1%[1]	Stdev	EF1%[1]	Stdev	EF1%[1]	Stdev
Somatostatin receptor 5	34.50	25.23	48.11	22.95	55.44	22.34	66.88	14.09	72.34	10.57
Macrophage colony stimulating factor receptor	14.47	8.65	23.04	12.31	32.22	12.55	39.96	11.49	45.30	12.06
Tyrosine-protein kinase ABL	25.15	18.19	36.08	17.03	45.57	11.24	49.11	9.83	51.42	6.21
Vasopressin V1a receptor	11.04	5.76	15.23	4.17	20.23	5.22	23.21	4.53	26.76	6.16
Melatonin receptor 1B	39.00	16.08	51.51	8.95	57.52	6.84	62.02	6.17	64.52	5.47
Thymidylate synthase	19.13	16.87	27.63	17.87	38.00	16.97	44.16	15.80	51.64	13.06
Insulin-like growth factor I receptor	22.53	9.35	28.33	8.93	33.16	8.54	36.03	6.97	37.83	7.17
Tyrosine-protein kinase receptor FLT3	6.41	3.98	9.52	4.91	13.12	5.05	15.07	5.50	17.14	5.57
Serotonin 1d (5-HT1d) receptor	20.05	15.01	28.98	13.28	32.49	12.02	35.59	13.70	42.95	12.22
Inhibitor of nuclear factor κ B kinase β subunit	38.04	26.29	53.91	16.71	59.10	10.46	62.49	4.10	64.21	4.72
Glucocorticoid receptor	9.13	4.34	13.30	5.37	19.17	4.99	22.75	5.09	26.71	5.69
Oxytocin receptor	17.29	9.96	31.67	9.47	40.33	11.88	47.69	13.47	52.12	12.94
Progesterone receptor	11.55	9.17	17.31	10.70	19.69	9.36	22.53	10.11	25.40	9.77
β_2-adrenergic receptor	22.13	11.34	25.97	11.99	30.14	13.12	33.28	9.80	36.86	9.17

c-Jun N-terminal kinase I	19.43	11.99	29.44	12.24	37.58	8.09	41.22	7.52	42.42	6.57
Cyclooxygenase-2	13.15	13.94	22.40	11.94	24.87	11.18	29.63	10.76	33.20	9.65
Glycine transporter 1	15.58	7.64	26.72	9.95	36.75	9.83	41.91	9.26	46.47	7.72
Dopamine receptor D3	16.83	11.23	20.92	9.14	23.80	7.95	24.63	7.66	26.35	7.10
C5a anaphylatoxin chemotactic receptor	28.31	15.31	36.79	15.53	45.39	12.47	51.85	11.07	55.46	9.78
C-C chemokine receptor type 4	21.00	15.80	37.23	14.96	43.04	12.89	48.06	13.61	55.52	10.66
Interleukin-8 receptor B	19.12	8.73	32.14	11.27	40.35	11.21	46.44	11.91	50.22	11.46
Muscarinic acetylcholine receptor M3	14.58	12.48	18.75	12.49	21.66	12.05	26.31	11.54	29.10	9.99
Phosphodiesterase 4A	9.80	4.21	15.70	3.73	21.56	4.54	25.03	5.64	28.94	7.46
Liver glycogen phosphorylase	26.67	9.01	42.43	13.10	52.85	14.80	60.92	13.37	65.31	14.71
Tyrosine-protein kinase SRC	14.02	8.92	19.53	11.19	24.22	9.44	26.57	8.46	28.95	6.96
Renin	12.24	4.86	19.57	7.02	24.01	5.87	27.57	6.51	30.36	5.47
Phosphodiesterase 4D	7.30	6.72	12.32	8.48	16.36	10.54	18.23	10.40	22.22	11.06
Cathepsin S	7.41	3.95	10.19	4.04	13.60	4.62	16.49	4.55	18.67	3.90
Carbonic anhydrase XII	5.17	4.20	6.18	3.62	7.74	4.33	8.75	4.12	9.50	4.15
Cytochrome P450 2C9	3.08	3.36	5.85	4.08	7.15	4.55	8.85	4.25	9.67	4.05
Urotensin II receptor	14.70	6.98	22.43	7.13	29.75	8.10	37.65	8.35	42.47	9.01
Metabotropic glutamate receptor 1	26.38	12.84	41.22	15.88	51.59	16.70	59.61	15.85	68.24	11.35
Nicotinic acid receptor 1	34.37	13.30	52.40	13.31	63.54	13.17	70.72	12.78	76.58	12.02
Cathepsin L	8.37	5.85	12.32	6.39	15.91	5.44	18.16	5.41	20.40	5.76
C-C chemokine receptor type 2	15.79	10.80	22.96	11.66	30.56	9.14	33.26	8.62	35.10	7.95
Cathepsin B	10.61	6.14	17.07	8.20	21.88	7.72	25.87	7.18	28.07	7.82
Estradiol 17-β-dehydrogenase 3	25.67	11.17	42.58	13.92	55.70	15.50	66.55	14.56	70.17	12.06
Sodium channel protein type IX α subunit	30.55	20.98	40.48	16.79	47.39	7.61	48.39	6.95	49.75	1.66
Voltage-gated potassium channel subunit Kv1.5	8.68	4.69	16.12	4.69	19.78	5.93	24.72	6.67	28.68	7.10
Sphingosine 1-phosphate receptor Edg-1	20.52	13.34	22.23	12.73	24.81	12.93	28.32	13.82	29.83	13.88

Neuropeptide Y receptor type 5	17.42	9.71	25.73	9.90	33.74	10.51	41.88	8.91	47.58	9.00
Ghrelin receptor	10.14	8.93	15.33	8.22	20.09	8.05	24.35	8.21	27.51	8.55
Leukotriene A4 hydrolase	31.24	15.95	42.17	16.45	50.69	18.55	56.92	18.60	60.37	17.65
Serine/threonine-protein kinase Aurora-A	11.43	11.54	17.22	11.46	19.45	10.14	25.01	6.74	26.61	6.94
Neuropeptide Y receptor type 1	7.28	6.79	8.96	6.73	11.25	7.08	14.06	6.43	16.19	6.76
Orexin receptor 2	19.99	13.17	31.14	10.07	37.08	8.99	41.57	7.99	44.86	8.11
P2X purinoceptor 7	15.63	6.11	22.97	5.27	29.55	6.03	35.57	5.88	39.31	4.75
β-secretase 1	14.82	11.26	22.72	10.69	27.23	9.55	31.81	5.83	32.60	5.50
G protein-coupled receptor 44	17.92	9.30	25.46	6.58	28.79	6.60	32.98	6.40	36.61	6.60
Serine/threonine-protein kinase B-raf	10.28	5.31	14.89	5.68	17.96	5.72	22.34	5.41	25.62	5.79
Average	17.52	10.53	25.66	10.38	31.48	9.77	36.06	9.04	39.48	8.39

[1] Reported values are averages obtained from 30 screening runs with different (sets of) query molecules.

Table S14. Mean EF10% Values and Standard Deviations Obtained with the 3D Shape-based Approach for the Individual Targets Using Query Sets of Different Size.

Target Name	One query		Two queries		Three queries		Four queries		Five queries	
	EF10%[1]	Stdev	EF10%[1]	Stdev	EF10%[1]	Stdev	EF10%[1]	Stdev	EF10%[1]	Stdev
Somatostatin receptor 5	4.95	2.84	6.27	2.11	6.86	1.86	7.65	1.10	8.01	0.66
Macrophage colony stimulating factor receptor	2.25	1.11	2.99	1.33	3.81	1.19	4.59	1.16	5.04	1.28
Tyrosine-protein kinase ABL	3.54	1.78	4.87	1.37	5.82	0.72	6.05	0.70	6.21	0.56
Vasopressin V1a receptor	2.33	1.20	2.81	0.91	3.40	0.94	3.66	0.85	4.04	0.76
Melatonin receptor 1B	5.89	1.57	7.22	0.67	7.73	0.46	8.04	0.46	8.20	0.44
Thymidylate synthase	2.91	1.77	3.95	1.62	4.93	1.47	5.48	1.48	6.12	1.25
Insulin-like growth factor I receptor	3.97	1.34	4.53	1.08	4.94	1.08	5.34	0.96	5.56	0.96

Tyrosine-protein kinase receptor FLT3	1.46	0.75	1.58	0.70	2.04	0.61	2.25	0.63	2.47	0.65
Serotonin 1d (5-HT1d) receptor	3.57	1.92	4.56	1.60	4.70	1.36	5.02	1.49	5.86	1.15
Inhibitor of nuclear factor κ B kinase β subunit	4.30	2.62	5.93	1.72	6.47	1.13	6.87	0.41	7.02	0.44
Glucocorticoid receptor	1.74	0.82	2.25	0.93	2.89	0.82	3.11	0.65	3.54	0.65
Oxytocin receptor	2.36	1.00	3.74	0.97	4.66	1.28	5.46	1.42	5.91	1.37
Progesterone receptor	2.46	1.33	3.19	1.49	3.70	1.17	4.16	1.24	4.38	1.24
β_2-adrenergic receptor	3.65	1.45	4.07	1.49	4.44	1.35	4.80	1.02	5.22	0.92
c-Jun N-terminal kinase I	2.67	1.27	3.77	1.44	4.89	0.92	5.28	0.90	5.43	0.77
Cyclooxygenase-2	2.25	1.75	3.61	1.45	3.85	1.37	4.43	1.19	4.85	1.03
Glycine transporter 1	2.35	0.79	3.45	1.08	4.66	1.04	5.15	0.90	5.59	0.77
Dopamine receptor D3	4.03	2.38	4.57	1.81	4.82	1.61	4.72	1.45	4.97	1.35
C5a anaphylatoxin chemotactic receptor	3.47	1.50	4.25	1.52	5.06	1.28	5.66	1.14	6.03	1.01
C-C chemokine receptor type 4	2.78	1.77	4.46	1.67	5.12	1.39	5.61	1.43	6.39	1.06
Interleukin-8 receptor B	2.85	0.90	4.31	1.25	5.41	1.18	6.05	1.03	6.44	1.00
Muscarinic acetylcholine receptor M3	2.68	1.59	2.99	1.55	3.48	1.51	3.96	1.20	4.27	0.95
Phosphodiesterase 4A	2.02	0.76	2.70	0.57	3.31	0.68	3.78	0.67	4.16	0.75
Liver glycogen phosphorylase	3.66	0.95	5.45	1.36	6.45	1.66	7.23	1.24	7.65	1.25
Tyrosine-protein kinase SRC	2.69	1.36	3.23	1.47	3.74	1.13	4.02	1.06	4.29	0.84
Renin	2.35	1.08	2.96	0.85	3.44	0.82	3.86	0.88	4.08	0.70
Phosphodiesterase 4D	1.96	1.25	2.51	1.20	2.76	1.27	2.94	1.20	3.38	1.30
Cathepsin S	1.66	0.83	1.91	0.70	2.39	0.82	2.77	0.80	3.05	0.77
Carbonic anhydrase XII	1.72	1.06	1.99	0.97	2.40	1.05	2.64	0.93	2.79	0.90
Cytochrome P450 2C9	0.83	0.50	1.09	0.53	1.21	0.57	1.40	0.52	1.53	0.47
Urotensin II receptor	2.30	1.19	3.00	1.10	3.85	1.04	4.59	0.99	5.12	0.99
Metabotropic glutamate receptor 1	3.59	1.39	5.03	1.83	6.06	1.94	6.94	1.67	7.86	0.87
Nicotinic acid receptor 1	3.99	1.39	6.02	1.80	7.37	1.65	8.15	1.45	8.66	1.32

Cathepsin L	1.64	1.00	2.10	1.05	2.60	0.86	2.84	0.78	3.09	0.80
C-C chemokine receptor type 2	3.00	1.61	3.75	1.41	4.66	1.13	5.12	1.00	5.37	1.02
Cathepsin B	1.86	0.91	2.46	0.93	3.12	0.88	3.61	0.87	3.83	0.90
Estradiol 17-β-dehydrogenase 3	3.26	1.17	5.00	1.49	6.37	1.69	7.33	1.58	7.67	1.27
Sodium channel protein type IX α subunit	4.54	2.81	5.69	2.28	6.62	1.07	6.73	1.04	7.04	0.38
Voltage-gated potassium channel subunit Kv1.5	1.70	0.82	2.44	0.63	2.80	0.75	3.36	0.80	3.73	0.90
Sphingosine 1-phosphate receptor Edg-1	4.16	2.28	4.42	2.05	4.61	2.00	5.09	1.90	5.19	1.85
Neuropeptide Y receptor type 5	3.26	1.75	4.34	1.66	5.24	1.45	6.13	1.17	6.68	1.13
Ghrelin receptor	1.64	1.14	2.21	1.16	2.64	1.08	3.06	1.09	3.33	1.09
Leukotriene A4 hydrolase	5.63	1.92	6.33	1.57	6.76	1.51	7.18	1.31	7.54	1.21
Serine/threonine-protein kinase Aurora-A	1.96	1.38	2.48	1.33	2.77	1.13	3.48	0.74	3.70	0.83
Neuropeptide Y receptor type 1	1.42	0.99	1.53	0.90	1.73	0.89	2.01	0.79	2.23	0.79
Orexin receptor 2	3.23	1.51	4.49	0.96	5.09	0.77	5.46	0.68	5.78	0.69
P2X purinoceptor 7	3.14	0.89	4.22	0.81	5.15	0.88	5.85	0.56	6.15	0.50
β-secretase 1	2.03	1.15	3.04	1.11	3.58	1.15	4.23	0.84	4.35	0.80
G protein-coupled receptor 44	3.78	1.60	4.79	1.12	5.28	1.06	6.03	0.86	6.51	0.91
Serine/threonine-protein kinase B-raf	2.04	0.85	2.66	0.74	3.01	0.78	3.56	0.78	3.92	0.82
Average	2.87	1.38	3.74	1.27	4.37	1.15	4.85	1.02	5.20	0.93

[1] Reported values are averages obtained from 30 screening runs with different (sets of) query molecules.

Table S15. Mean SRR1% values and Standard Deviations Obtained with the 2D Fingerprint-based Approach for the Individual Targets Using Query Sets of Different Size.

Target Name	One query		Two queries		Three queries		Four queries		Five queries	
	SRR1%[1]	Stdev	SRR1%[1]	Stdev	SRR1%[1]	Stdev	SRR1%[1]	Stdev	SRR1%[1]	Stdev

Somatostatin receptor 5	0.32	0.16	0.44	0.16	0.55	0.16	0.62	0.13	0.65	0.11
Macrophage colony stimulating factor receptor	0.14	0.09	0.24	0.11	0.30	0.11	0.36	0.11	0.40	0.10
Tyrosine-protein kinase ABL	0.20	0.11	0.28	0.12	0.36	0.12	0.38	0.11	0.43	0.07
Vasopressin V1a receptor	0.11	0.05	0.17	0.05	0.21	0.05	0.25	0.05	0.29	0.05
Melatonin receptor 1B	0.41	0.16	0.52	0.12	0.58	0.10	0.64	0.07	0.68	0.06
Thymidylate synthase	0.18	0.10	0.27	0.11	0.35	0.09	0.45	0.06	0.49	0.07
Insulin-like growth factor I receptor	0.28	0.09	0.36	0.09	0.42	0.07	0.45	0.05	0.47	0.05
Tyrosine-protein kinase receptor FLT3	0.08	0.05	0.11	0.04	0.15	0.05	0.18	0.04	0.21	0.04
Serotonin 1d (5-HT1d) receptor	0.22	0.15	0.31	0.14	0.42	0.08	0.46	0.06	0.48	0.06
Inhibitor of nuclear factor κ B kinase β subunit	0.31	0.17	0.44	0.08	0.46	0.03	0.48	0.03	0.49	0.04
Glucocorticoid receptor	0.16	0.11	0.22	0.11	0.26	0.08	0.30	0.08	0.33	0.08
Oxytocin receptor	0.13	0.05	0.22	0.08	0.31	0.08	0.34	0.07	0.39	0.07
Progesterone receptor	0.13	0.09	0.19	0.12	0.24	0.12	0.27	0.12	0.29	0.11
β_2-adrenergic receptor	0.42	0.19	0.51	0.15	0.55	0.11	0.58	0.11	0.62	0.09
c-Jun N-terminal kinase I	0.21	0.11	0.31	0.09	0.36	0.08	0.41	0.08	0.43	0.08
Cyclooxygenase-2	0.17	0.13	0.20	0.10	0.24	0.11	0.26	0.09	0.29	0.09
Glycine transporter 1	0.17	0.07	0.29	0.10	0.38	0.09	0.44	0.10	0.49	0.09
Dopamine receptor D3	0.16	0.07	0.22	0.06	0.27	0.06	0.30	0.04	0.31	0.04
C5a anaphylatoxin chemotactic receptor	0.38	0.15	0.53	0.20	0.64	0.18	0.74	0.15	0.80	0.14
C-C chemokine receptor type 4	0.20	0.10	0.30	0.11	0.39	0.11	0.44	0.10	0.49	0.11
Interleukin-8 receptor B	0.17	0.07	0.28	0.08	0.37	0.08	0.41	0.09	0.46	0.09
Muscarinic acetylcholine receptor M3	0.14	0.11	0.20	0.11	0.25	0.10	0.30	0.10	0.32	0.10
Phosphodiesterase 4A	0.11	0.06	0.19	0.09	0.25	0.08	0.30	0.08	0.34	0.09
Liver glycogen phosphorylase	0.30	0.11	0.47	0.12	0.56	0.14	0.61	0.15	0.68	0.13
Tyrosine-protein kinase SRC	0.18	0.11	0.26	0.10	0.29	0.10	0.34	0.09	0.37	0.08
Renin	0.25	0.09	0.34	0.09	0.40	0.07	0.48	0.10	0.51	0.11

Phosphodiesterase 4D	0.09	0.06	0.13	0.07	0.18	0.07	0.21	0.07	0.26	0.08
Cathepsin S	0.13	0.06	0.18	0.06	0.22	0.05	0.26	0.04	0.29	0.05
Carbonic anhydrase XII	0.17	0.10	0.20	0.09	0.21	0.08	0.22	0.06	0.23	0.06
Cytochrome P450 2C9	0.03	0.02	0.04	0.02	0.05	0.02	0.06	0.02	0.07	0.02
Urotensin II receptor	0.23	0.11	0.33	0.11	0.38	0.11	0.47	0.11	0.53	0.10
Metabotropic glutamate receptor 1	0.23	0.09	0.36	0.12	0.45	0.13	0.54	0.11	0.57	0.10
Nicotinic acid receptor 1	0.33	0.13	0.51	0.18	0.61	0.15	0.68	0.14	0.74	0.14
Cathepsin L	0.16	0.08	0.21	0.08	0.28	0.07	0.33	0.06	0.35	0.06
C-C chemokine receptor type 2	0.28	0.13	0.39	0.13	0.43	0.10	0.47	0.08	0.50	0.07
Cathepsin B	0.18	0.09	0.26	0.07	0.32	0.08	0.36	0.08	0.40	0.08
Estradiol 17-β-dehydrogenase 3	0.26	0.09	0.45	0.15	0.57	0.15	0.66	0.14	0.71	0.12
Sodium channel protein type IX α subunit	0.28	0.16	0.35	0.10	0.40	0.05	0.42	0.03	0.42	0.03
Voltage-gated potassium channel subunit Kv1.5	0.09	0.05	0.14	0.06	0.19	0.08	0.25	0.08	0.29	0.08
Sphingosine 1-phosphate receptor Edg-1	0.17	0.10	0.22	0.09	0.26	0.08	0.31	0.08	0.34	0.08
Neuropeptide Y receptor type 5	0.19	0.12	0.30	0.13	0.39	0.14	0.46	0.11	0.50	0.11
Ghrelin receptor	0.12	0.11	0.18	0.11	0.26	0.11	0.33	0.12	0.39	0.10
Leukotriene A4 hydrolase	0.32	0.16	0.45	0.15	0.57	0.15	0.63	0.14	0.70	0.11
Serine/threonine-protein kinase Aurora-A	0.13	0.11	0.23	0.09	0.24	0.09	0.27	0.09	0.29	0.09
Neuropeptide Y receptor type 1	0.07	0.06	0.13	0.07	0.16	0.09	0.19	0.09	0.21	0.08
Orexin receptor 2	0.16	0.09	0.24	0.10	0.31	0.09	0.37	0.09	0.42	0.09
P2X purinoceptor 7	0.17	0.06	0.23	0.07	0.26	0.05	0.32	0.07	0.34	0.07
β-secretase 1	0.15	0.07	0.20	0.07	0.23	0.04	0.25	0.04	0.25	0.04
G protein-coupled receptor 44	0.17	0.08	0.23	0.08	0.27	0.06	0.29	0.06	0.30	0.05
Serine/threonine-protein kinase B-raf	0.15	0.07	0.21	0.07	0.23	0.08	0.27	0.08	0.30	0.07
Average	0.20	0.10	0.28	0.10	0.34	0.09	0.39	0.09	0.42	0.08

[1] Reported values are averages obtained from 30 screening runs with different (sets of) query molecules.

Table S16. Mean SRR10% values and Standard Deviations Obtained with the 2D Fingerprint-based Approach for the Individual Targets Using Query Sets of Different Size.

Target Name	One query		Two queries		Three queries		Four queries		Five queries	
	SRR10%[1]	Stdev	SRR10%[1]	Stdev	SRR10%[1]	Stdev	SRR10%[1]	Stdev	SRR10%[1]	Stdev
Somatostatin receptor 5	0.48	0.15	0.61	0.15	0.70	0.14	0.75	0.11	0.76	0.11
Macrophage colony stimulating factor receptor	0.28	0.12	0.39	0.10	0.44	0.10	0.51	0.10	0.55	0.09
Tyrosine-protein kinase ABL	0.39	0.15	0.49	0.11	0.54	0.09	0.55	0.09	0.58	0.07
Vasopressin V1a receptor	0.26	0.10	0.32	0.09	0.37	0.10	0.42	0.10	0.46	0.08
Melatonin receptor 1B	0.71	0.17	0.84	0.09	0.88	0.05	0.91	0.04	0.93	0.02
Thymidylate synthase	0.35	0.14	0.43	0.11	0.52	0.11	0.59	0.06	0.63	0.08
Insulin-like growth factor I receptor	0.58	0.13	0.68	0.10	0.71	0.07	0.74	0.07	0.75	0.06
Tyrosine-protein kinase receptor FLT3	0.25	0.11	0.30	0.09	0.33	0.08	0.37	0.06	0.41	0.06
Serotonin 1d (5-HT1d) receptor	0.42	0.17	0.52	0.14	0.62	0.08	0.66	0.06	0.69	0.06
Inhibitor of nuclear factor κ B kinase β subunit	0.45	0.16	0.55	0.09	0.58	0.05	0.59	0.05	0.60	0.05
Glucocorticoid receptor	0.31	0.17	0.38	0.15	0.43	0.12	0.48	0.10	0.52	0.09
Oxytocin receptor	0.26	0.08	0.36	0.09	0.42	0.09	0.46	0.08	0.51	0.09
Progesterone receptor	0.28	0.13	0.34	0.16	0.41	0.15	0.45	0.15	0.48	0.14
β_2-adrenergic receptor	0.70	0.16	0.77	0.08	0.81	0.05	0.82	0.04	0.83	0.03
c-Jun N-terminal kinase I	0.39	0.14	0.51	0.10	0.56	0.11	0.61	0.11	0.64	0.11
Cyclooxygenase-2	0.36	0.18	0.42	0.14	0.46	0.11	0.49	0.07	0.52	0.06
Glycine transporter 1	0.31	0.08	0.43	0.11	0.53	0.10	0.58	0.10	0.62	0.11
Dopamine receptor D3	0.44	0.14	0.52	0.12	0.59	0.10	0.63	0.08	0.66	0.08
C5a anaphylatoxin chemotactic receptor	0.49	0.16	0.67	0.20	0.77	0.18	0.86	0.12	0.90	0.09

C-C chemokine receptor type 4	0.32	0.15	0.42	0.14	0.49	0.13	0.52	0.12	0.57	0.13
Interleukin-8 receptor B	0.32	0.12	0.44	0.10	0.54	0.09	0.58	0.08	0.62	0.09
Muscarinic acetylcholine receptor M3	0.29	0.15	0.38	0.13	0.43	0.13	0.46	0.11	0.50	0.11
Phosphodiesterase 4A	0.24	0.10	0.32	0.12	0.38	0.10	0.45	0.09	0.49	0.09
Liver glycogen phosphorylase	0.48	0.13	0.62	0.12	0.69	0.12	0.72	0.12	0.77	0.08
Tyrosine-protein kinase SRC	0.39	0.16	0.48	0.13	0.51	0.10	0.55	0.09	0.58	0.07
Renin	0.51	0.12	0.60	0.09	0.68	0.08	0.75	0.07	0.76	0.07
Phosphodiesterase 4D	0.25	0.11	0.30	0.11	0.34	0.11	0.37	0.10	0.43	0.10
Cathepsin S	0.36	0.10	0.42	0.10	0.47	0.09	0.51	0.06	0.55	0.07
Carbonic anhydrase XII	0.47	0.21	0.55	0.16	0.58	0.12	0.59	0.09	0.61	0.07
Cytochrome P450 2C9	0.12	0.05	0.13	0.05	0.14	0.05	0.15	0.04	0.16	0.04
Urotensin II receptor	0.45	0.12	0.52	0.08	0.56	0.08	0.63	0.08	0.66	0.07
Metabotropic glutamate receptor 1	0.33	0.10	0.43	0.12	0.51	0.13	0.60	0.11	0.63	0.10
Nicotinic acid receptor 1	0.43	0.15	0.62	0.19	0.72	0.18	0.79	0.14	0.85	0.13
Cathepsin L	0.38	0.15	0.49	0.12	0.58	0.09	0.63	0.08	0.67	0.07
C-C chemokine receptor type 2	0.56	0.20	0.67	0.12	0.69	0.08	0.71	0.08	0.75	0.07
Cathepsin B	0.41	0.14	0.52	0.11	0.56	0.10	0.59	0.10	0.65	0.09
Estradiol 17-β-dehydrogenase 3	0.38	0.16	0.52	0.16	0.62	0.16	0.70	0.14	0.75	0.11
Sodium channel protein type IX α subunit	0.52	0.24	0.64	0.10	0.68	0.05	0.70	0.03	0.71	0.03
Voltage-gated potassium channel subunit Kv1.5	0.20	0.09	0.27	0.08	0.32	0.10	0.38	0.10	0.41	0.10
Sphingosine 1-phosphate receptor Edg-1	0.37	0.16	0.45	0.14	0.52	0.10	0.57	0.09	0.61	0.09
Neuropeptide Y receptor type 5	0.36	0.16	0.48	0.14	0.57	0.15	0.65	0.14	0.70	0.13
Ghrelin receptor	0.24	0.12	0.30	0.13	0.38	0.13	0.45	0.14	0.51	0.11
Leukotriene A4 hydrolase	0.62	0.23	0.72	0.19	0.78	0.17	0.83	0.14	0.89	0.08
Serine/threonine-protein kinase Aurora-A	0.33	0.12	0.43	0.09	0.45	0.08	0.49	0.09	0.51	0.09
Neuropeptide Y receptor type 1	0.14	0.07	0.19	0.08	0.22	0.10	0.25	0.10	0.27	0.09

Orexin receptor 2	0.34	0.14	0.42	0.13	0.49	0.10	0.55	0.09	0.59	0.11
P2X purinoceptor 7	0.38	0.08	0.43	0.06	0.46	0.06	0.50	0.08	0.52	0.07
β-secretase 1	0.33	0.12	0.39	0.12	0.45	0.09	0.48	0.10	0.50	0.11
G protein-coupled receptor 44	0.50	0.14	0.58	0.10	0.64	0.09	0.66	0.07	0.68	0.07
Serine/threonine-protein kinase B-raf	0.38	0.11	0.47	0.11	0.52	0.10	0.58	0.07	0.60	0.07
Average	0.38	0.14	0.47	0.12	0.53	0.10	0.58	0.09	0.61	0.08

[1] Reported values are averages obtained from 30 screening runs with different (sets of) query molecules.

Table S17. Mean SRR1% values and Standard Deviations Obtained with the 3D Shape-based Approach for the Individual Targets Using Query Sets of Different Size.

Target Name	One query		Two queries		Three queries		Four queries		Five queries	
	SRR1%[1]	Stdev	SRR1%[1]	Stdev	SRR1%[1]	Stdev	SRR1%[1]	Stdev	SRR1%[1]	Stdev
Somatostatin receptor 5	0.30	0.20	0.41	0.19	0.48	0.19	0.58	0.12	0.64	0.09
Macrophage colony stimulating factor receptor	0.13	0.09	0.20	0.12	0.29	0.12	0.36	0.11	0.41	0.11
Tyrosine-protein kinase ABL	0.20	0.12	0.27	0.11	0.34	0.08	0.36	0.08	0.38	0.06
Vasopressin V1a receptor	0.11	0.07	0.15	0.04	0.19	0.05	0.22	0.04	0.25	0.06
Melatonin receptor 1B	0.37	0.13	0.48	0.08	0.53	0.06	0.57	0.05	0.59	0.04
Thymidylate synthase	0.16	0.09	0.22	0.09	0.29	0.08	0.35	0.08	0.40	0.08
Insulin-like growth factor I receptor	0.24	0.08	0.30	0.07	0.35	0.06	0.38	0.06	0.40	0.05
Tyrosine-protein kinase receptor FLT3	0.06	0.04	0.08	0.04	0.10	0.04	0.12	0.04	0.13	0.03
Serotonin 1d (5-HT1d) receptor	0.17	0.11	0.24	0.10	0.27	0.09	0.30	0.10	0.35	0.09
Inhibitor of nuclear factor κ B kinase β subunit	0.30	0.19	0.42	0.12	0.46	0.08	0.49	0.05	0.51	0.05
Glucocorticoid receptor	0.10	0.07	0.13	0.06	0.19	0.07	0.22	0.06	0.26	0.07
Oxytocin receptor	0.14	0.05	0.23	0.06	0.29	0.07	0.35	0.07	0.37	0.07

Progesterone receptor	0.12	0.09	0.17	0.11	0.19	0.09	0.22	0.10	0.24	0.10
β_2-adrenergic receptor	0.23	0.13	0.26	0.12	0.30	0.14	0.32	0.11	0.35	0.10
c-Jun N-terminal kinase I	0.20	0.13	0.29	0.13	0.36	0.09	0.39	0.07	0.40	0.07
Cyclooxygenase-2	0.13	0.13	0.21	0.11	0.23	0.10	0.27	0.09	0.30	0.08
Glycine transporter 1	0.15	0.06	0.25	0.09	0.35	0.10	0.39	0.09	0.44	0.08
Dopamine receptor D3	0.17	0.11	0.21	0.09	0.23	0.08	0.24	0.07	0.25	0.07
C5a anaphylatoxin chemotactic receptor	0.29	0.17	0.40	0.18	0.49	0.15	0.56	0.13	0.60	0.11
C-C chemokine receptor type 4	0.16	0.08	0.26	0.06	0.33	0.06	0.40	0.07	0.46	0.09
Interleukin-8 receptor B	0.19	0.08	0.31	0.10	0.38	0.10	0.43	0.09	0.47	0.10
Muscarinic acetylcholine receptor M3	0.17	0.15	0.22	0.15	0.25	0.14	0.30	0.13	0.33	0.11
Phosphodiesterase 4A	0.11	0.06	0.17	0.05	0.23	0.06	0.26	0.07	0.29	0.08
Liver glycogen phosphorylase	0.26	0.11	0.40	0.14	0.50	0.15	0.56	0.13	0.59	0.14
Tyrosine-protein kinase SRC	0.15	0.10	0.20	0.12	0.25	0.09	0.27	0.08	0.29	0.07
Renin	0.15	0.07	0.22	0.07	0.27	0.07	0.31	0.08	0.33	0.05
Phosphodiesterase 4D	0.07	0.05	0.11	0.06	0.14	0.07	0.16	0.07	0.19	0.08
Cathepsin S	0.08	0.04	0.10	0.04	0.14	0.05	0.16	0.04	0.18	0.04
Carbonic anhydrase XII	0.07	0.05	0.08	0.04	0.09	0.05	0.10	0.05	0.11	0.05
Cytochrome P450 2C9	0.02	0.02	0.04	0.02	0.05	0.03	0.06	0.02	0.06	0.02
Urotensin II receptor	0.15	0.07	0.22	0.07	0.30	0.08	0.37	0.08	0.42	0.09
Metabotropic glutamate receptor 1	0.25	0.10	0.38	0.12	0.47	0.13	0.54	0.13	0.61	0.09
Nicotinic acid receptor 1	0.36	0.16	0.55	0.15	0.66	0.15	0.73	0.13	0.79	0.12
Cathepsin L	0.09	0.06	0.13	0.06	0.16	0.06	0.19	0.06	0.21	0.06
C-C chemokine receptor type 2	0.15	0.10	0.22	0.10	0.29	0.08	0.32	0.08	0.34	0.09
Cathepsin B	0.11	0.06	0.17	0.08	0.23	0.08	0.26	0.08	0.29	0.08
Estradiol 17-β-dehydrogenase 3	0.24	0.10	0.40	0.14	0.50	0.16	0.59	0.13	0.61	0.13
Sodium channel protein type IX α subunit	0.28	0.19	0.35	0.15	0.40	0.07	0.41	0.06	0.42	0.02

Voltage-gated potassium channel subunit Kv1.5	0.09	0.05	0.15	0.05	0.19	0.06	0.24	0.07	0.28	0.08
Sphingosine 1-phosphate receptor Edg-1	0.23	0.14	0.23	0.13	0.25	0.15	0.29	0.15	0.30	0.15
Neuropeptide Y receptor type 5	0.20	0.14	0.28	0.14	0.36	0.14	0.44	0.12	0.49	0.12
Ghrelin receptor	0.10	0.08	0.15	0.08	0.19	0.07	0.23	0.07	0.26	0.07
Leukotriene A4 hydrolase	0.35	0.19	0.45	0.19	0.53	0.20	0.59	0.21	0.62	0.19
Serine/threonine-protein kinase Aurora-A	0.10	0.10	0.14	0.10	0.16	0.09	0.20	0.06	0.21	0.06
Neuropeptide Y receptor type 1	0.06	0.05	0.07	0.05	0.08	0.05	0.10	0.05	0.11	0.04
Orexin receptor 2	0.20	0.13	0.32	0.10	0.37	0.11	0.42	0.10	0.45	0.11
P2X purinoceptor 7	0.11	0.04	0.16	0.04	0.21	0.05	0.26	0.04	0.28	0.04
β-secretase 1	0.12	0.07	0.17	0.06	0.20	0.06	0.23	0.04	0.23	0.03
G protein-coupled receptor 44	0.20	0.11	0.27	0.08	0.29	0.09	0.32	0.08	0.36	0.08
Serine/threonine-protein kinase B-raf	0.09	0.05	0.13	0.05	0.15	0.05	0.19	0.05	0.21	0.05
Average	0.17	0.10	0.24	0.09	0.29	0.09	0.33	0.08	0.36	0.08

[1] Reported values are averages obtained from 30 screening runs with different (sets of) query molecules.

Table S18. Mean SRR10% Values and Standard Deviations Obtained by the 3D Shape-based Approach for the Individual Targets Using Query Sets of Different Size.

Target Name	One query		Two queries		Three queries		Four queries		Five queries	
	SRR10%[1]	Stdev	SRR10%[1]	Stdev	SRR10%[1]	Stdev	SRR10%[1]	Stdev	SRR10%[1]	Stdev
Somatostatin receptor 5	0.44	0.22	0.54	0.17	0.60	0.16	0.67	0.09	0.71	0.06
Macrophage colony stimulating factor receptor	0.22	0.12	0.28	0.13	0.35	0.11	0.42	0.11	0.46	0.11
Tyrosine-protein kinase ABL	0.31	0.12	0.40	0.09	0.45	0.08	0.47	0.07	0.49	0.07
Vasopressin V1a receptor	0.24	0.13	0.28	0.08	0.32	0.09	0.34	0.08	0.38	0.06
Melatonin receptor 1B	0.55	0.12	0.67	0.07	0.71	0.05	0.74	0.05	0.75	0.05

Thymidylate synthase	0.28	0.14	0.36	0.11	0.43	0.09	0.49	0.09	0.53	0.09
Insulin-like growth factor I receptor	0.42	0.10	0.48	0.08	0.52	0.08	0.56	0.07	0.58	0.07
Tyrosine-protein kinase receptor FLT3	0.17	0.10	0.16	0.08	0.19	0.07	0.21	0.06	0.22	0.05
Serotonin 1d (5-HT1d) receptor	0.33	0.15	0.40	0.13	0.41	0.11	0.45	0.12	0.51	0.09
Inhibitor of nuclear factor κ B kinase β subunit	0.37	0.19	0.50	0.13	0.55	0.09	0.58	0.05	0.60	0.05
Glucocorticoid receptor	0.19	0.10	0.23	0.10	0.30	0.09	0.32	0.07	0.36	0.07
Oxytocin receptor	0.22	0.07	0.31	0.07	0.38	0.08	0.43	0.09	0.47	0.09
Progesterone receptor	0.25	0.13	0.31	0.14	0.36	0.11	0.40	0.12	0.41	0.11
β_2-adrenergic receptor	0.38	0.16	0.41	0.15	0.43	0.14	0.46	0.11	0.50	0.09
c-Jun N-terminal kinase I	0.28	0.13	0.38	0.15	0.48	0.09	0.51	0.09	0.52	0.08
Cyclooxygenase-2	0.24	0.18	0.36	0.14	0.38	0.13	0.42	0.12	0.45	0.10
Glycine transporter 1	0.23	0.07	0.33	0.10	0.44	0.10	0.49	0.08	0.54	0.07
Dopamine receptor D3	0.42	0.23	0.47	0.18	0.49	0.16	0.48	0.15	0.50	0.14
C5a anaphylatoxin chemotactic receptor	0.37	0.16	0.46	0.17	0.55	0.15	0.62	0.13	0.65	0.11
C-C chemokine receptor type 4	0.23	0.10	0.34	0.09	0.40	0.08	0.47	0.08	0.53	0.10
Interleukin-8 receptor B	0.29	0.09	0.43	0.12	0.53	0.10	0.59	0.09	0.63	0.09
Muscarinic acetylcholine receptor M3	0.29	0.17	0.33	0.17	0.38	0.16	0.43	0.12	0.46	0.09
Phosphodiesterase 4A	0.23	0.09	0.30	0.07	0.37	0.08	0.42	0.08	0.45	0.08
Liver glycogen phosphorylase	0.36	0.10	0.50	0.13	0.59	0.14	0.65	0.11	0.68	0.10
Tyrosine-protein kinase SRC	0.28	0.15	0.33	0.16	0.38	0.12	0.40	0.10	0.42	0.08
Renin	0.26	0.14	0.33	0.08	0.38	0.08	0.42	0.08	0.45	0.07
Phosphodiesterase 4D	0.21	0.10	0.26	0.09	0.28	0.09	0.30	0.09	0.33	0.09
Cathepsin S	0.19	0.10	0.20	0.07	0.25	0.09	0.28	0.08	0.31	0.08
Carbonic anhydrase XII	0.20	0.11	0.23	0.10	0.27	0.11	0.30	0.09	0.31	0.09
Cytochrome P450 2C9	0.08	0.04	0.10	0.04	0.10	0.04	0.11	0.04	0.12	0.03
Urotensin II receptor	0.24	0.11	0.30	0.11	0.39	0.10	0.46	0.10	0.52	0.09

Metabotropic glutamate receptor 1	0.38	0.10	0.51	0.15	0.61	0.16	0.69	0.15	0.77	0.08
Nicotinic acid receptor 1	0.41	0.15	0.61	0.18	0.74	0.16	0.82	0.14	0.87	0.13
Cathepsin L	0.19	0.11	0.23	0.11	0.28	0.10	0.30	0.09	0.33	0.09
C-C chemokine receptor type 2	0.30	0.14	0.37	0.13	0.46	0.10	0.50	0.09	0.52	0.11
Cathepsin B	0.21	0.11	0.26	0.09	0.32	0.09	0.37	0.09	0.40	0.09
Estradiol 17-β-dehydrogenase 3	0.35	0.11	0.52	0.13	0.62	0.15	0.70	0.13	0.71	0.12
Sodium channel protein type IX α subunit	0.44	0.26	0.54	0.22	0.62	0.11	0.63	0.10	0.66	0.03
Voltage-gated potassium channel subunit Kv1.5	0.18	0.09	0.25	0.07	0.29	0.07	0.34	0.08	0.37	0.09
Sphingosine 1-phosphate receptor Edg-1	0.44	0.23	0.46	0.20	0.47	0.20	0.52	0.19	0.53	0.18
Neuropeptide Y receptor type 5	0.35	0.19	0.45	0.19	0.55	0.16	0.63	0.14	0.68	0.13
Ghrelin receptor	0.18	0.11	0.23	0.12	0.27	0.10	0.30	0.10	0.33	0.10
Leukotriene A4 hydrolase	0.59	0.22	0.65	0.17	0.69	0.17	0.75	0.14	0.78	0.13
Serine/threonine-protein kinase Aurora-A	0.20	0.13	0.23	0.12	0.24	0.10	0.30	0.07	0.31	0.08
Neuropeptide Y receptor type 1	0.15	0.09	0.15	0.08	0.16	0.07	0.18	0.07	0.19	0.06
Orexin receptor 2	0.34	0.14	0.46	0.10	0.52	0.10	0.56	0.10	0.60	0.10
P2X purinoceptor 7	0.26	0.06	0.33	0.06	0.39	0.06	0.44	0.04	0.47	0.05
β-secretase 1	0.18	0.08	0.26	0.08	0.30	0.08	0.35	0.06	0.36	0.06
G protein-coupled receptor 44	0.41	0.18	0.51	0.13	0.55	0.12	0.63	0.09	0.67	0.09
Serine/threonine-protein kinase B-raf	0.21	0.09	0.26	0.08	0.29	0.08	0.34	0.07	0.37	0.08
Average	0.29	0.13	0.37	0.12	0.42	0.11	0.46	0.10	0.50	0.09

[1] Reported values are averages obtained from 30 screening runs with different (sets of) query molecules.

Table S19. Overall EFs and SRRs Obtained with the Parallel Selection Approach at Different Ratios of Hits Selected from the Hit Lists.

Proportion of hits selected from the hit lists obtained with the 2D approach	EF1%	EF10%	SRR1%	SRR10%
0.0	17.52	2.87	0.17	0.29
0.1	24.25	4.04	0.23	0.40
0.2	26.27	4.21	0.25	0.42
0.3	27.26	4.32	0.26	0.43
0.4	27.85	4.40	0.27	0.44
0.5	28.16	4.44	0.27	0.45
0.6	28.18	4.51	0.27	0.45
0.7	27.94	4.53	0.27	0.46
0.8	27.29	4.54	0.26	0.46
0.9	25.76	4.50	0.25	0.45
1.0	19.96	3.69	0.20	0.38

Table S20. Overall AUC Values, EFs and SRRs Obtained with the Integrated Approach.

Query set size	AUC	Stdev	Enrichment factor				Scaffold recovery rate			
			1%	Stdev	10%	Stdev	1%	Stdev	10%	Stdev
1	0.70	0.10	28.16	11.14	4.44	1.35	0.27	0.10	0.45	0.13
2	0.76	0.07	38.49	9.95	5.51	1.10	0.37	0.09	0.54	0.10
3	0.80	0.06	45.48	8.51	6.20	0.89	0.43	0.08	0.60	0.08
4	0.82	0.05	50.38	7.31	6.65	0.76	0.47	0.07	0.64	0.07
5	0.84	0.04	53.82	6.57	6.97	0.69	0.50	0.07	0.67	0.07

Table S21. Mean AUC Values and Standard Deviations Obtained by the Integrated Approach for the Individual Targets Using Query Sets of Different Size.

Target Name	One query		Two queries		Three queries		Four queries		Five queries	
	AUC[1]	Stdev	AUC[1]	Stdev	AUC[1]	Stdev	AUC[1]	Stdev	AUC[1]	Stdev
Somatostatin receptor 5	0.84	0.09	0.90	0.05	0.91	0.05	0.93	0.04	0.94	0.04
Macrophage colony stimulating factor receptor	0.66	0.09	0.73	0.08	0.77	0.07	0.82	0.05	0.85	0.06
Tyrosine-protein kinase ABL	0.76	0.09	0.82	0.06	0.83	0.03	0.84	0.03	0.85	0.02
Vasopressin V1a receptor	0.64	0.09	0.68	0.07	0.75	0.07	0.77	0.06	0.78	0.05
Melatonin receptor 1B	0.92	0.06	0.96	0.03	0.97	0.02	0.98	0.01	0.99	0.01
Thymidylate synthase	0.70	0.11	0.78	0.07	0.83	0.07	0.87	0.05	0.89	0.04
Insulin-like growth factor I receptor	0.84	0.05	0.87	0.04	0.88	0.03	0.89	0.03	0.89	0.03
Tyrosine-protein kinase receptor FLT3	0.57	0.09	0.58	0.08	0.63	0.08	0.67	0.08	0.70	0.07
Serotonin 1d (5-HT1d) receptor	0.78	0.08	0.83	0.05	0.86	0.03	0.88	0.02	0.89	0.02
Inhibitor of nuclear factor κ B kinase β subunit	0.79	0.08	0.82	0.03	0.83	0.03	0.84	0.03	0.85	0.03
Glucocorticoid receptor	0.59	0.13	0.66	0.11	0.71	0.08	0.74	0.04	0.77	0.05
Oxytocin receptor	0.65	0.10	0.75	0.10	0.81	0.09	0.85	0.07	0.87	0.06
Progesterone receptor	0.62	0.11	0.68	0.10	0.73	0.08	0.77	0.08	0.78	0.09
β_2-adrenergic receptor	0.88	0.08	0.91	0.03	0.92	0.02	0.93	0.01	0.93	0.01
c-Jun N-terminal kinase I	0.70	0.09	0.77	0.08	0.82	0.05	0.84	0.05	0.85	0.06
Cyclooxygenase-2	0.66	0.13	0.73	0.07	0.75	0.05	0.76	0.04	0.78	0.03
Glycine transporter 1	0.66	0.06	0.76	0.07	0.84	0.05	0.88	0.04	0.90	0.04
Dopamine receptor D3	0.79	0.08	0.81	0.06	0.84	0.04	0.85	0.03	0.85	0.03
C5a anaphylatoxin chemotactic receptor	0.78	0.18	0.87	0.12	0.93	0.08	0.95	0.06	0.97	0.02
C-C chemokine receptor type 4	0.68	0.14	0.77	0.10	0.81	0.07	0.85	0.05	0.88	0.04
Interleukin-8 receptor B	0.67	0.09	0.77	0.07	0.83	0.06	0.86	0.05	0.87	0.06

Muscarinic acetylcholine receptor M3	0.67	0.08	0.70	0.06	0.74	0.06	0.77	0.04	0.80	0.03
Phosphodiesterase 4A	0.58	0.09	0.64	0.08	0.71	0.06	0.75	0.06	0.76	0.07
Liver glycogen phosphorylase	0.82	0.06	0.88	0.05	0.91	0.04	0.93	0.03	0.94	0.02
Tyrosine-protein kinase SRC	0.71	0.12	0.76	0.07	0.79	0.04	0.81	0.04	0.82	0.04
Renin	0.76	0.07	0.82	0.05	0.85	0.04	0.87	0.03	0.88	0.04
Phosphodiesterase 4D	0.61	0.09	0.65	0.07	0.69	0.07	0.71	0.07	0.75	0.06
Cathepsin S	0.65	0.08	0.70	0.08	0.73	0.08	0.76	0.06	0.80	0.06
Carbonic anhydrase XII	0.68	0.14	0.75	0.08	0.78	0.06	0.79	0.04	0.80	0.04
Cytochrome P450 2C9	0.45	0.07	0.46	0.06	0.47	0.06	0.49	0.05	0.50	0.04
Urotensin II receptor	0.72	0.07	0.78	0.05	0.82	0.05	0.85	0.04	0.86	0.03
Metabotropic glutamate receptor 1	0.74	0.13	0.82	0.11	0.88	0.08	0.91	0.04	0.92	0.02
Nicotinic acid receptor 1	0.69	0.11	0.85	0.14	0.92	0.11	0.95	0.08	0.97	0.06
Cathepsin L	0.62	0.11	0.69	0.06	0.75	0.04	0.78	0.04	0.80	0.05
C-C chemokine receptor type 2	0.79	0.12	0.87	0.04	0.89	0.02	0.90	0.02	0.91	0.02
Cathepsin B	0.65	0.12	0.72	0.07	0.75	0.06	0.78	0.05	0.80	0.05
Estradiol 17-β-dehydrogenase 3	0.72	0.11	0.81	0.12	0.89	0.08	0.93	0.03	0.94	0.01
Sodium channel protein type IX α subunit	0.81	0.11	0.84	0.05	0.87	0.03	0.87	0.02	0.88	0.02
Voltage-gated potassium channel subunit Kv1.5	0.55	0.11	0.63	0.07	0.68	0.08	0.72	0.08	0.74	0.09
Sphingosine 1-phosphate receptor Edg-1	0.75	0.12	0.79	0.08	0.81	0.07	0.84	0.06	0.86	0.04
Neuropeptide Y receptor type 5	0.69	0.13	0.79	0.08	0.84	0.07	0.89	0.07	0.90	0.06
Ghrelin receptor	0.58	0.11	0.65	0.11	0.69	0.10	0.72	0.10	0.75	0.09
Leukotriene A4 hydrolase	0.89	0.09	0.93	0.04	0.95	0.04	0.96	0.03	0.97	0.03
Serine/threonine-protein kinase Aurora-A	0.66	0.11	0.72	0.06	0.74	0.05	0.77	0.04	0.79	0.04
Neuropeptide Y receptor type 1	0.50	0.11	0.53	0.11	0.56	0.09	0.58	0.08	0.60	0.07
Orexin receptor 2	0.70	0.08	0.77	0.05	0.80	0.03	0.82	0.03	0.84	0.03
P2X purinoceptor 7	0.75	0.05	0.79	0.04	0.82	0.03	0.84	0.02	0.85	0.02

β-secretase 1	0.62	0.08	0.67	0.07	0.72	0.06	0.74	0.06	0.76	0.06
G protein-coupled receptor 44	0.80	0.08	0.85	0.05	0.88	0.04	0.90	0.03	0.92	0.02
Serine/threonine-protein kinase B-raf	0.71	0.08	0.77	0.07	0.80	0.05	0.83	0.04	0.85	0.04
Average	0.70	0.10	0.76	0.07	0.80	0.06	0.82	0.05	0.84	0.04

[1] Reported values are averages obtained from 30 screening runs with different (sets of) query molecules.

Table S22. Mean EF1% Values and Standard Deviations Obtained with the Integrated Approach for the Individual Targets Using Query Sets of Different Size.

Target Name	One query		Two queries		Three queries		Four queries		Five queries	
	EF1%[1]	Stdev	EF1%[1]	Stdev	EF1%[1]	Stdev	EF1%[1]	Stdev	EF1%[1]	Stdev
Somatostatin receptor 5	56.52	22.98	69.77	13.20	77.40	10.22	81.30	3.96	83.61	3.94
Macrophage colony stimulating factor receptor	23.33	8.01	38.07	12.83	48.44	9.74	56.23	7.76	61.15	9.37
Tyrosine-protein kinase ABL	35.86	16.36	46.03	14.41	53.42	10.34	56.39	7.78	60.09	4.42
Vasopressin V1a receptor	17.11	5.06	25.11	4.67	32.77	5.84	37.09	5.18	41.71	5.02
Melatonin receptor 1B	52.21	15.74	64.85	9.15	70.33	8.60	76.06	6.75	79.67	4.22
Thymidylate synthase	32.08	17.72	44.72	17.37	54.53	14.50	62.67	11.15	70.44	9.82
Insulin-like growth factor I receptor	33.87	9.57	40.41	8.50	45.23	5.94	48.22	4.08	49.54	4.16
Tyrosine-protein kinase receptor FLT3	11.09	5.77	15.50	6.24	22.08	6.85	26.51	7.01	31.03	6.45
Serotonin 1d (5-HT1d) receptor	35.09	17.13	45.36	14.98	54.19	8.84	57.91	6.75	61.63	5.82
Inhibitor of nuclear factor κ B kinase β subunit	52.96	13.92	61.42	3.59	63.65	3.97	65.40	4.54	67.35	5.04
Glucocorticoid receptor	19.56	8.55	28.66	8.00	34.59	5.90	39.78	4.89	43.98	5.39
Oxytocin receptor	29.68	13.55	47.07	12.63	56.66	12.90	63.89	8.86	67.23	9.04
Progesterone receptor	18.38	8.70	25.78	12.14	31.88	11.31	36.27	11.32	39.19	11.32
β_2-adrenergic receptor	46.18	14.73	54.76	12.23	60.76	8.34	62.98	7.55	66.39	6.90

c-Jun N-terminal kinase I	29.63	11.87	39.59	11.64	46.18	8.12	50.80	8.71	52.15	8.95
Cyclooxygenase-2	19.76	11.69	27.32	8.58	31.66	7.52	34.14	6.97	38.06	6.64
Glycine transporter 1	27.14	7.80	43.67	10.30	54.52	8.76	61.89	7.63	66.68	7.79
Dopamine receptor D3	21.56	7.79	26.65	6.31	30.07	5.13	31.71	3.57	32.69	3.33
C5a anaphylatoxin chemotactic receptor	55.14	21.96	67.45	17.11	79.01	15.00	85.29	12.25	89.90	6.33
C-C chemokine receptor type 4	37.85	17.32	52.46	13.90	61.55	11.85	70.04	8.95	74.51	8.87
Interleukin-8 receptor B	28.31	7.99	43.61	9.05	54.78	10.41	60.85	9.95	63.29	10.74
Muscarinic acetylcholine receptor M3	20.36	10.67	26.98	9.95	32.76	8.69	38.17	8.03	42.06	7.72
Phosphodiesterase 4A	17.28	6.65	27.16	8.82	36.57	8.89	41.63	9.20	45.50	10.43
Liver glycogen phosphorylase	44.03	13.62	64.03	10.87	73.52	10.47	79.34	11.01	85.95	5.06
Tyrosine-protein kinase SRC	23.76	11.17	32.93	9.55	37.54	7.19	41.48	7.46	44.72	6.76
Renin	25.45	6.25	37.91	6.63	43.24	5.37	49.18	5.74	52.77	6.57
Phosphodiesterase 4D	14.08	9.69	21.34	9.26	28.77	11.00	32.67	11.05	39.15	10.58
Cathepsin S	15.22	6.02	22.22	6.98	26.64	7.67	31.09	5.79	35.67	5.04
Carbonic anhydrase XII	12.07	5.82	14.42	4.28	16.22	4.49	17.47	3.94	18.46	3.61
Cytochrome P450 2C9	5.53	3.47	8.94	3.44	11.06	3.75	13.20	3.59	13.94	3.12
Urotensin II receptor	29.76	10.85	42.99	9.80	51.40	9.06	59.37	8.43	64.88	6.47
Metabotropic glutamate receptor 1	40.65	16.72	55.21	15.81	66.91	12.47	73.48	8.91	76.04	5.35
Nicotinic acid receptor 1	46.81	14.60	63.76	12.67	74.24	11.18	81.36	10.80	86.18	8.91
Cathepsin L	16.73	7.04	23.16	6.85	30.15	6.16	35.27	5.76	38.29	6.05
C-C chemokine receptor type 2	32.09	13.30	44.89	10.90	50.00	7.18	53.30	5.46	55.98	5.51
Cathepsin B	20.05	8.33	29.65	8.35	35.98	7.67	40.85	6.74	44.54	6.17
Estradiol 17-β-dehydrogenase 3	48.35	14.52	65.60	17.29	78.77	12.07	85.89	5.61	88.41	1.69
Sodium channel protein type IX α subunit	33.24	13.24	42.35	9.48	48.37	3.14	49.56	2.59	50.22	0.60
Voltage-gated potassium channel subunit Kv1.5	15.51	6.42	25.76	7.90	32.94	8.58	39.39	9.37	43.36	8.71
Sphingosine 1-phosphate receptor Edg-1	25.45	11.62	29.85	9.91	35.20	9.54	38.91	10.26	41.79	9.03

Neuropeptide Y receptor type 5	27.57	11.17	41.26	10.17	51.49	8.75	59.83	8.53	63.76	8.64
Ghrelin receptor	18.42	11.15	28.29	11.94	35.56	10.31	41.76	10.34	46.57	10.54
Leukotriene A4 hydrolase	45.28	15.51	58.17	14.38	69.49	15.45	77.35	12.82	79.75	12.59
Serine/threonine-protein kinase Aurora-A	18.02	12.63	29.73	8.24	31.95	8.20	36.23	5.43	38.99	5.14
Neuropeptide Y receptor type 1	13.35	8.68	21.82	8.70	26.94	8.16	31.17	8.15	34.54	8.19
Orexin receptor 2	27.16	12.42	37.96	9.46	45.01	8.04	50.75	6.71	55.07	4.93
P2X purinoceptor 7	25.54	8.74	35.28	7.45	41.48	5.40	46.80	4.61	49.22	5.18
β-secretase 1	20.98	6.77	27.65	6.74	32.46	4.45	34.52	2.87	35.06	2.32
G protein-coupled receptor 44	22.55	7.87	31.36	6.95	35.85	5.20	39.03	4.74	41.52	4.80
Serine/threonine-protein kinase B-raf	19.52	8.02	25.46	7.78	29.57	6.80	34.51	6.08	38.12	5.13
Average	28.16	11.14	38.49	9.95	45.48	8.51	50.38	7.31	53.82	6.57

[1] Reported values are averages obtained from 30 screening runs with different (sets of) query molecules.

Table S23. Mean EF10% Values and Standard Deviations Obtained with the Integrated Approach for the Individual Targets Using Query Sets of Different Size.

Target Name	One query		Two queries		Three queries		Four queries		Five queries	
	EF10%[1]	Stdev	EF10%[1]	Stdev	EF10%[1]	Stdev	EF10%[1]	Stdev	EF10%[1]	Stdev
Somatostatin receptor 5	6.94	1.68	8.13	0.79	8.45	0.67	8.65	0.49	8.79	0.48
Macrophage colony stimulating factor receptor	3.22	0.66	4.78	1.28	5.77	0.95	6.58	0.84	7.06	0.98
Tyrosine-protein kinase ABL	5.39	1.70	6.37	1.16	6.87	0.69	7.06	0.54	7.26	0.40
Vasopressin V1a receptor	3.05	0.94	4.01	0.82	4.83	0.88	5.29	0.80	5.62	0.67
Melatonin receptor 1B	7.59	1.42	9.01	0.53	9.36	0.39	9.57	0.30	9.68	0.18
Thymidylate synthase	4.82	1.73	5.67	1.68	6.57	1.32	7.26	1.02	7.93	0.88
Insulin-like growth factor I receptor	6.34	1.24	6.80	0.96	7.40	0.67	7.68	0.58	7.75	0.60

Tyrosine-protein kinase receptor FLT3	2.58	0.77	2.78	0.73	3.53	0.80	4.00	0.80	4.53	0.71
Serotonin 1d (5-HT1d) receptor	5.63	1.53	6.50	1.01	7.13	0.64	7.55	0.47	7.78	0.46
Inhibitor of nuclear factor κ B kinase β subunit	6.36	1.00	6.81	0.38	6.99	0.43	7.18	0.45	7.36	0.48
Glucocorticoid receptor	3.02	1.19	4.20	1.05	4.99	0.84	5.48	0.57	5.89	0.58
Oxytocin receptor	4.14	1.35	5.54	1.31	6.34	1.34	7.15	0.97	7.54	0.99
Progesterone receptor	3.32	1.40	4.19	1.51	5.02	1.28	5.54	1.26	5.86	1.40
β_2-adrenergic receptor	7.55	1.39	8.13	0.93	8.49	0.54	8.62	0.38	8.77	0.27
c-Jun N-terminal kinase I	4.59	1.41	5.36	1.26	6.10	0.88	6.62	0.96	6.76	1.01
Cyclooxygenase-2	3.81	1.76	4.92	1.04	5.43	0.67	5.63	0.59	5.96	0.52
Glycine transporter 1	3.64	0.71	5.42	1.19	6.82	0.74	7.33	0.68	7.76	0.70
Dopamine receptor D3	4.84	1.51	5.74	1.11	6.34	0.71	6.57	0.58	6.72	0.56
C5a anaphylatoxin chemotactic receptor	5.46	2.40	7.84	1.58	8.67	1.30	9.12	1.02	9.46	0.43
C-C chemokine receptor type 4	4.73	2.01	6.20	1.31	6.92	1.06	7.63	0.76	8.07	0.68
Interleukin-8 receptor B	4.30	1.03	5.74	0.99	6.87	1.01	7.29	0.83	7.56	0.93
Muscarinic acetylcholine receptor M3	3.50	1.37	4.34	1.03	5.02	0.90	5.53	0.64	5.95	0.60
Phosphodiesterase 4A	2.64	0.76	3.94	1.00	5.05	0.88	5.66	0.93	6.01	1.03
Liver glycogen phosphorylase	5.87	1.42	7.53	1.21	8.30	0.92	8.71	0.68	9.02	0.28
Tyrosine-protein kinase SRC	4.11	1.70	5.13	1.17	5.57	0.77	5.94	0.80	6.18	0.75
Renin	4.61	0.88	5.74	0.76	6.27	0.67	6.89	0.66	7.02	0.67
Phosphodiesterase 4D	2.49	1.08	3.61	1.15	4.30	1.23	4.74	1.13	5.54	1.09
Cathepsin S	3.49	1.10	4.09	1.05	4.68	1.11	5.17	0.87	5.75	0.83
Carbonic anhydrase XII	3.65	2.11	4.87	1.35	5.14	1.18	5.32	0.95	5.50	0.83
Cytochrome P450 2C9	1.38	0.60	1.64	0.50	1.86	0.53	2.14	0.46	2.24	0.41
Urotensin II receptor	4.56	1.21	5.48	1.13	6.21	0.94	6.69	0.75	7.16	0.55
Metabotropic glutamate receptor 1	4.60	2.17	6.34	1.73	7.38	1.26	7.97	0.83	8.18	0.44
Nicotinic acid receptor 1	5.69	1.65	7.55	1.73	8.57	1.30	9.12	1.06	9.45	0.78

Cathepsin L	3.72	1.10	4.40	0.97	5.36	0.66	5.91	0.70	6.21	0.77
C-C chemokine receptor type 2	5.51	1.92	6.82	1.13	7.34	0.65	7.63	0.58	7.96	0.49
Cathepsin B	3.85	1.43	4.77	1.03	5.43	0.92	5.94	0.89	6.39	0.77
Estradiol 17-β-dehydrogenase 3	5.34	1.54	6.93	1.78	8.23	1.19	8.87	0.50	9.05	0.11
Sodium channel protein type IX α subunit	6.79	0.69	7.00	0.84	7.35	0.35	7.45	0.36	7.57	0.33
Voltage-gated potassium channel subunit Kv1.5	2.62	0.84	3.61	0.80	4.38	1.05	5.01	1.07	5.43	1.08
Sphingosine 1-phosphate receptor Edg-1	4.94	2.04	5.72	1.41	6.16	1.36	6.63	1.21	6.96	0.98
Neuropeptide Y receptor type 5	4.10	1.63	5.98	1.28	6.97	0.94	7.71	0.96	8.04	0.90
Ghrelin receptor	2.75	1.48	3.84	1.50	4.58	1.27	5.25	1.30	5.71	1.26
Leukotriene A4 hydrolase	7.22	1.74	8.02	1.27	8.52	1.28	8.99	1.04	9.14	0.95
Serine/threonine-protein kinase Aurora-A	3.88	1.19	4.52	0.84	4.81	0.84	5.31	0.68	5.69	0.59
Neuropeptide Y receptor type 1	1.77	1.05	2.71	1.00	3.22	0.90	3.65	0.88	4.03	0.83
Orexin receptor 2	4.23	1.34	5.23	0.96	5.95	0.62	6.36	0.67	6.78	0.57
P2X purinoceptor 7	4.80	0.81	5.79	0.70	6.43	0.51	6.88	0.48	7.08	0.48
β-secretase 1	3.72	1.25	4.62	0.95	5.35	0.68	5.79	0.77	6.00	0.72
G protein-coupled receptor 44	5.28	1.20	6.46	1.12	7.04	0.90	7.53	0.68	7.82	0.71
Serine/threonine-protein kinase B-raf	3.79	1.29	4.89	1.06	5.39	0.87	5.93	0.79	6.33	0.66
Average	4.44	1.35	5.51	1.10	6.20	0.89	6.65	0.76	6.97	0.69

[1] Reported values are averages obtained from 30 screening runs with different (sets of) query molecules.

Table S24. Mean SRR1% Values and Standard Deviations Obtained with the Integrated Approach for the Individual Targets Using Query Sets of Different Size.

Target Name	One query		Two queries		Three queries		Four queries		Five queries	
	SRR1%[1]	Stdev	SRR1%[1]	Stdev	SRR1%[1]	Stdev	SRR1%[1]	Stdev	SRR1%[1]	Stdev
Somatostatin receptor 5	0.49	0.18	0.61	0.11	0.69	0.09	0.73	0.05	0.76	0.06
Macrophage colony stimulating factor receptor	0.21	0.08	0.33	0.13	0.42	0.10	0.49	0.07	0.54	0.08
Tyrosine-protein kinase ABL	0.29	0.11	0.37	0.11	0.42	0.09	0.45	0.07	0.48	0.05
Vasopressin V1a receptor	0.17	0.05	0.25	0.04	0.32	0.05	0.35	0.04	0.39	0.04
Melatonin receptor 1B	0.54	0.15	0.66	0.11	0.71	0.10	0.77	0.07	0.81	0.05
Thymidylate synthase	0.28	0.10	0.38	0.12	0.47	0.10	0.56	0.09	0.62	0.09
Insulin-like growth factor I receptor	0.36	0.07	0.43	0.06	0.46	0.04	0.49	0.04	0.50	0.04
Tyrosine-protein kinase receptor FLT3	0.11	0.06	0.14	0.05	0.19	0.05	0.22	0.04	0.25	0.04
Serotonin 1d (5-HT1d) receptor	0.31	0.13	0.39	0.12	0.46	0.08	0.50	0.07	0.54	0.06
Inhibitor of nuclear factor κ B kinase β subunit	0.41	0.09	0.48	0.04	0.50	0.04	0.52	0.05	0.55	0.06
Glucocorticoid receptor	0.21	0.10	0.28	0.09	0.34	0.06	0.38	0.05	0.42	0.06
Oxytocin receptor	0.22	0.07	0.34	0.07	0.42	0.07	0.47	0.06	0.50	0.08
Progesterone receptor	0.18	0.10	0.26	0.13	0.31	0.12	0.34	0.12	0.36	0.11
β_2-adrenergic receptor	0.45	0.15	0.52	0.13	0.57	0.09	0.58	0.07	0.60	0.06
c-Jun N-terminal kinase I	0.30	0.13	0.38	0.12	0.44	0.08	0.48	0.08	0.49	0.09
Cyclooxygenase-2	0.21	0.11	0.27	0.08	0.32	0.06	0.33	0.05	0.36	0.05
Glycine transporter 1	0.27	0.08	0.44	0.10	0.55	0.09	0.61	0.09	0.65	0.09
Dopamine receptor D3	0.22	0.07	0.27	0.06	0.30	0.04	0.32	0.03	0.33	0.03
C5a anaphylatoxin chemotactic receptor	0.55	0.22	0.68	0.16	0.79	0.13	0.85	0.10	0.88	0.06
C-C chemokine receptor type 4	0.29	0.11	0.42	0.10	0.51	0.11	0.60	0.10	0.66	0.09

Interleukin-8 receptor B	0.28	0.08	0.43	0.08	0.53	0.10	0.59	0.10	0.62	0.11
Muscarinic acetylcholine receptor M3	0.23	0.13	0.30	0.12	0.36	0.10	0.41	0.09	0.44	0.08
Phosphodiesterase 4A	0.18	0.08	0.29	0.11	0.38	0.10	0.43	0.10	0.46	0.11
Liver glycogen phosphorylase	0.42	0.15	0.60	0.12	0.67	0.11	0.72	0.11	0.77	0.05
Tyrosine-protein kinase SRC	0.25	0.12	0.34	0.10	0.38	0.07	0.41	0.06	0.44	0.05
Renin	0.30	0.08	0.42	0.10	0.48	0.07	0.53	0.07	0.57	0.07
Phosphodiesterase 4D	0.13	0.07	0.20	0.08	0.26	0.08	0.29	0.09	0.34	0.10
Cathepsin S	0.16	0.06	0.22	0.07	0.27	0.07	0.31	0.04	0.35	0.05
Carbonic anhydrase XII	0.15	0.07	0.17	0.05	0.19	0.06	0.20	0.05	0.21	0.05
Cytochrome P450 2C9	0.04	0.02	0.06	0.02	0.07	0.02	0.08	0.02	0.09	0.02
Urotensin II receptor	0.30	0.10	0.43	0.10	0.51	0.09	0.59	0.08	0.64	0.06
Metabotropic glutamate receptor 1	0.38	0.13	0.51	0.13	0.61	0.10	0.67	0.09	0.70	0.06
Nicotinic acid receptor 1	0.49	0.16	0.67	0.14	0.77	0.11	0.83	0.10	0.88	0.08
Cathepsin L	0.18	0.08	0.25	0.06	0.32	0.05	0.36	0.05	0.39	0.06
C-C chemokine receptor type 2	0.31	0.11	0.41	0.10	0.46	0.06	0.49	0.06	0.52	0.06
Cathepsin B	0.22	0.09	0.31	0.08	0.38	0.08	0.42	0.07	0.46	0.06
Estradiol 17-β-dehydrogenase 3	0.45	0.14	0.64	0.13	0.73	0.06	0.77	0.04	0.79	0.02
Sodium channel protein type IX α subunit	0.30	0.11	0.37	0.08	0.40	0.03	0.41	0.03	0.42	0.02
Voltage-gated potassium channel subunit Kv1.5	0.15	0.07	0.25	0.09	0.31	0.09	0.38	0.11	0.42	0.10
Sphingosine 1-phosphate receptor Edg-1	0.27	0.11	0.31	0.10	0.34	0.11	0.37	0.11	0.40	0.09
Neuropeptide Y receptor type 5	0.29	0.13	0.43	0.12	0.53	0.10	0.61	0.09	0.64	0.10
Ghrelin receptor	0.18	0.10	0.28	0.11	0.35	0.09	0.41	0.09	0.45	0.10
Leukotriene A4 hydrolase	0.48	0.17	0.61	0.16	0.72	0.15	0.80	0.12	0.82	0.12
Serine/threonine-protein kinase Aurora-A	0.16	0.11	0.25	0.07	0.26	0.07	0.30	0.06	0.32	0.05
Neuropeptide Y receptor type 1	0.11	0.07	0.17	0.07	0.21	0.07	0.24	0.07	0.27	0.06
Orexin receptor 2	0.27	0.12	0.39	0.10	0.46	0.09	0.52	0.09	0.57	0.08

P2X purinoceptor 7	0.21	0.06	0.28	0.06	0.33	0.06	0.36	0.05	0.37	0.06
β-secretase 1	0.17	0.05	0.22	0.05	0.24	0.03	0.25	0.02	0.25	0.02
G protein-coupled receptor 44	0.24	0.08	0.32	0.07	0.36	0.06	0.38	0.06	0.40	0.06
Serine/threonine-protein kinase B-raf	0.19	0.08	0.24	0.07	0.27	0.06	0.31	0.06	0.34	0.05
Average	0.27	0.10	0.37	0.09	0.43	0.08	0.47	0.07	0.50	0.07

[1] Reported values are averages obtained from 30 screening runs with different (sets of) query molecules.

Table S25. Mean SRR10% Values and Standard Deviations Values Obtained with the Integrated Approach for the Individual Targets Using Query Sets of Different Size.

Target Name	One query		Two queries		Three queries		Four queries		Five queries	
	SRR10%[1]	Stdev	SRR10%[1]	Stdev	SRR10%[1]	Stdev	SRR10%[1]	Stdev	SRR10%[1]	Stdev
Somatostatin receptor 5	0.61	0.12	0.73	0.09	0.77	0.09	0.79	0.07	0.81	0.07
Macrophage colony stimulating factor receptor	0.32	0.06	0.45	0.11	0.53	0.08	0.61	0.07	0.65	0.09
Tyrosine-protein kinase ABL	0.49	0.13	0.56	0.10	0.59	0.07	0.60	0.06	0.62	0.05
Vasopressin V1a receptor	0.32	0.09	0.41	0.08	0.48	0.08	0.52	0.07	0.54	0.06
Melatonin receptor 1B	0.76	0.14	0.91	0.06	0.94	0.04	0.96	0.03	0.97	0.02
Thymidylate synthase	0.45	0.13	0.53	0.11	0.61	0.10	0.69	0.09	0.74	0.08
Insulin-like growth factor I receptor	0.66	0.09	0.71	0.07	0.74	0.06	0.76	0.05	0.77	0.05
Tyrosine-protein kinase receptor FLT3	0.30	0.10	0.30	0.08	0.35	0.07	0.39	0.06	0.43	0.06
Serotonin 1d (5-HT1d) receptor	0.52	0.12	0.60	0.09	0.66	0.07	0.70	0.06	0.73	0.05
Inhibitor of nuclear factor κ B kinase β subunit	0.55	0.08	0.59	0.05	0.62	0.05	0.63	0.05	0.65	0.05
Glucocorticoid receptor	0.33	0.15	0.43	0.11	0.51	0.09	0.55	0.06	0.59	0.05
Oxytocin receptor	0.35	0.08	0.46	0.08	0.51	0.08	0.58	0.07	0.62	0.09
Progesterone receptor	0.35	0.14	0.43	0.15	0.50	0.13	0.54	0.12	0.56	0.12

β_2-adrenergic receptor	0.73	0.14	0.77	0.09	0.80	0.06	0.81	0.04	0.82	0.03
c-Jun N-terminal kinase I	0.49	0.14	0.55	0.13	0.61	0.09	0.64	0.09	0.65	0.10
Cyclooxygenase-2	0.40	0.17	0.48	0.10	0.53	0.05	0.54	0.04	0.56	0.04
Glycine transporter 1	0.37	0.08	0.54	0.13	0.67	0.07	0.72	0.08	0.76	0.07
Dopamine receptor D3	0.51	0.15	0.59	0.11	0.65	0.07	0.67	0.06	0.68	0.06
C5a anaphylatoxin chemotactic receptor	0.56	0.25	0.79	0.14	0.87	0.10	0.91	0.08	0.93	0.04
C-C chemokine receptor type 4	0.39	0.15	0.51	0.13	0.58	0.12	0.66	0.11	0.72	0.09
Interleukin-8 receptor B	0.44	0.10	0.58	0.09	0.68	0.10	0.71	0.09	0.74	0.10
Muscarinic acetylcholine receptor M3	0.38	0.14	0.46	0.10	0.53	0.09	0.57	0.06	0.61	0.05
Phosphodiesterase 4A	0.30	0.11	0.43	0.12	0.53	0.10	0.59	0.10	0.61	0.10
Liver glycogen phosphorylase	0.55	0.11	0.68	0.11	0.75	0.08	0.79	0.06	0.81	0.04
Tyrosine-protein kinase SRC	0.44	0.17	0.53	0.12	0.57	0.07	0.60	0.06	0.62	0.06
Renin	0.52	0.10	0.64	0.10	0.70	0.08	0.75	0.07	0.77	0.07
Phosphodiesterase 4D	0.28	0.09	0.36	0.09	0.42	0.09	0.45	0.09	0.51	0.09
Cathepsin S	0.38	0.12	0.43	0.10	0.49	0.10	0.53	0.07	0.58	0.07
Carbonic anhydrase XII	0.39	0.21	0.52	0.14	0.54	0.12	0.56	0.09	0.58	0.08
Cytochrome P450 2C9	0.13	0.05	0.14	0.04	0.15	0.04	0.17	0.03	0.17	0.03
Urotensin II receptor	0.47	0.11	0.56	0.11	0.63	0.09	0.67	0.08	0.72	0.05
Metabotropic glutamate receptor 1	0.47	0.18	0.62	0.14	0.71	0.11	0.77	0.09	0.78	0.06
Nicotinic acid receptor 1	0.58	0.17	0.77	0.18	0.87	0.12	0.92	0.09	0.95	0.07
Cathepsin L	0.42	0.13	0.49	0.10	0.59	0.07	0.64	0.07	0.67	0.06
C-C chemokine receptor type 2	0.55	0.17	0.66	0.10	0.71	0.07	0.74	0.07	0.78	0.06
Cathepsin B	0.43	0.14	0.51	0.09	0.57	0.09	0.62	0.08	0.67	0.08
Estradiol 17-β-dehydrogenase 3	0.57	0.15	0.69	0.14	0.78	0.06	0.81	0.04	0.82	0.02
Sodium channel protein type IX α subunit	0.66	0.07	0.68	0.08	0.71	0.03	0.72	0.03	0.72	0.03
Voltage-gated potassium channel subunit Kv1.5	0.28	0.09	0.37	0.09	0.44	0.12	0.50	0.12	0.55	0.12

Sphingosine 1-phosphate receptor Edg-1	0.51	0.19	0.57	0.13	0.61	0.14	0.66	0.12	0.69	0.10
Neuropeptide Y receptor type 5	0.44	0.19	0.63	0.14	0.71	0.10	0.79	0.09	0.82	0.09
Ghrelin receptor	0.29	0.14	0.39	0.14	0.46	0.12	0.53	0.12	0.57	0.12
Leukotriene A4 hydrolase	0.75	0.20	0.82	0.13	0.87	0.12	0.92	0.10	0.94	0.08
Serine/threonine-protein kinase Aurora-A	0.38	0.11	0.42	0.09	0.44	0.08	0.48	0.08	0.52	0.07
Neuropeptide Y receptor type 1	0.17	0.08	0.25	0.09	0.28	0.09	0.32	0.09	0.35	0.07
Orexin receptor 2	0.44	0.13	0.55	0.09	0.62	0.08	0.67	0.09	0.71	0.09
P2X purinoceptor 7	0.40	0.05	0.47	0.06	0.52	0.07	0.56	0.07	0.58	0.07
β-secretase 1	0.34	0.10	0.41	0.09	0.47	0.08	0.51	0.09	0.52	0.08
G protein-coupled receptor 44	0.57	0.12	0.67	0.10	0.72	0.08	0.76	0.07	0.79	0.07
Serine/threonine-protein kinase B-raf	0.39	0.11	0.48	0.09	0.53	0.07	0.58	0.07	0.61	0.06
Average	0.45	0.13	0.54	0.10	0.60	0.08	0.64	0.07	0.67	0.07

[1] Reported values are averages obtained from 30 screening runs with different (sets of) query molecules.
